# Polyamine sequestration of 2**′**3**′**-cGAMP constrains intercellular transmission and STING engagement to subvert antitumor immunity

**DOI:** 10.1172/JCI201460

**Published:** 2026-06-01

**Authors:** Yunjin Ma, Chunyuan Zhao, Jiacheng Guo, Yue Fu, Wei Wang, Jiangong Zhang, Kun Zhao, Xiangbo Meng, Zhongshang Yuan, Chengjiang Gao, Mutian Jia, Ying Qin, Hui Song, Wei Zhao

**Affiliations:** 1 Key Laboratory of Infection and Immunity of Shandong Province, and Key Laboratory for Experimental Teratology of the Chinese Ministry of Education, School of Basic Medical Science, Cheeloo College of Medicine, Shandong University, Jinan, Shandong, China.; 2 State Key Laboratory for Innovation and Transformation of Luobing Theory, Key Laboratory of Cardiovascular Remodeling and Function Research of MOE, NHC, CAMS and Shandong Province, Department of Cardiology, Qilu Hospital of Shandong University, Jinan, Shandong, China.; 3 School of Pharmaceutical Sciences, Cheeloo College of Medicine, Shandong University, Jinan, Shandong, China.; 4 Advanced Medical Research Institute, Meili Lake Translational Research Park, Cheeloo College of Medicine, and; 5 Department of Biostatistics, School of Public Health, Cheeloo College of Medicine, Shandong University, Jinan, Shandong, China.

**Keywords:** Immunology, Metabolism, Cancer immunotherapy, Cellular immune response, Innate immunity

## Abstract

The cyclic dinucleotide 2′3′–cyclic guanosine monophosphate–adenosine monophosphate (2′3′-cGAMP) serves as a central immunotransmitter that propagates stimulator of interferon gene–dependent (STING-dependent) innate immunity across tissues; however, how microenvironmental metabolites regulate its spatiotemporal dynamics remains unknown. Here, we identified polyamines (spermine and spermidine) as critical rheostats controlling 2′3′-cGAMP functionality. Mechanistically, polyamines sequestered 2′3′-cGAMP into polymer-like aggregates, blocking intercellular propagation and suppressing intracellular STING activation by reducing ligand-receptor binding affinity. Deficiency of spermidine and spermine *N*^1^-acetyltransferase 1 (SAT1), the rate-limiting enzyme in polyamine catabolism, elevated polyamine levels to entrap extracellular 2′3′-cGAMP and inhibit STING activation. Synergistic administration of endogenous 2′3′-cGAMP with SAT1 stabilizer *N*^1^,*N*^11^-diethylnorspermine restored 2′3′-cGAMP bioavailability and STING signaling, facilitated type I interferon responses to reprogram immunologically suppressive tumors into immunologically active states and enhanced tumor clearance. Our study established polyamine–cGAMP interactions as a critical spatiotemporal regulatory mechanism for tissue-level immunity, providing a unified model for metabolite-mediated cyclic GMP-AMP synthase–STING (cGAS-STING) regulation across diseases.

## Abstract 2

Polyamines trap the immune messenger cGAMP, blocking its spread and activity to inhibit anti-tumor immunity.Correcting dysregulated polyamine metabolism can unleash cGAMP to boost immunotherapy.

## Graphical abstract

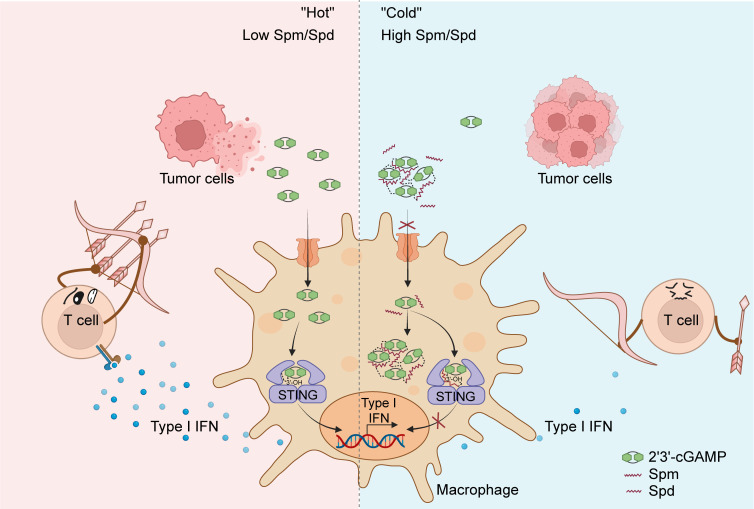

## Introduction

2′3′-cyclic GMP-AMP (cGAMP) is a cyclic dinucleotide (CDN) second messenger that serves as an endogenous ligand of stimulator of interferon genes (STING), bridging cytosolic DNA sensing via cyclic GMP-AMP synthase (cGAS) to orchestrate innate immunity (1–4). cGAS detects pathogen- or self-DNA emerging in the cytoplasm and then synthesizes 2′3′-cGAMP, which diffuses throughout the cell and directly binds to STING. 2′3′-cGAMP engagement induces STING oligomerization and trafficking from the ER to Golgi bodies, triggering TBK1-IRF3 signaling, and inducing type I interferon (IFN-α/β) production and NF-κB–mediated inflammatory cytokines and chemokines (e.g., CXCL10) (1). Moreover, 2′3′-cGAMP acts as a central immunotransmitter that spreads through gap junctions, transporters, extracellular vesicles, and viral particles to amplify STING activation and coordinate immune responses across cell types. Although essential for coordinating immunity against pathogens and tumors, dysregulated 2′3′-cGAMP propagation and activity drive autoimmunity, chronic inflammation, and cancer progression (2, 3).

2′3′-cGAMP production is initiated when cytosolic DNA induces phase separation with cGAS, where DNA length and divalent cations (e.g., Mn²^+^, Zn²^+^) critically enhance catalytic efficiency (5, 6). Intercellular propagation occurs through dedicated transport systems: SLC19A1, SLC46A2, LRRC8, and P2X7R import 2′3′-cGAMP across plasma membranes, whereas ABCC1/LRRC8 channels mediate its export (3). Gap junctions further enable spatial propagation to neighboring cells (7). Certain viruses (e.g., cytomegalovirus, lentivirus, herpesvirus) incorporate 2′3′-cGAMP into nascent viral particles to facilitate its spatial distribution (8, 9). Ectonucleotide pyrophosphatase phosphodiesterase 1 and sphingomyelin phosphodiesterase acid-like 3A degrade extracellular 2′3′-cGAMP to limit its paracrine signaling (10, 11). Functional activation requires 2′3′-cGAMP binding to STING dimers, with its unique 2′–5′ and 3′–5′ phosphodiester linkages in 2′3′-cGAMP enabling its high binding affinity with STING. This structural feature allows vertebrates to detect low concentrations of immunostimulatory DNA with exceptional sensitivity (12). Therefore, the 2′3′-cGAMP life cycle represents a finely tuned immunological rheostat, shaping immune outcomes across physiological and pathological contexts. However, how microenvironmental metabolites interact with these regulatory nodes to dictate immune fate decisions in diseased tissues remains unknown.

In this study, we demonstrate that endogenous polyamines (namely, spermine and spermidine) directly bind 2′3′-cGAMP to form polymer-like aggregates, blocking its intercellular propagation. Simultaneously, polyamine binding to the 3′-OH group of 2′3′-cGAMP reduces STING-binding affinity. This selectively suppresses intracellular 2′3′-cGAMP activity, attenuating STING activation and antitumor immunity. Deficiency of spermidine and spermine *N*^1^-acetyltransferase 1 (SAT1), the rate-limiting enzyme of polyamine catabolism, attenuates STING signaling and, consequently, suppresses antitumor immunity. Notably, synergistic administration of 2′3′-cGAMP and SAT1 stabilizer *N*^1^,*N*^11^-diethylnorspermine (DENSpm) depletes immunosuppressive polyamines, restores 2′3′-cGAMP bioavailability and STING signaling, and promotes tumor clearance through enhanced CD8^+^ T cell infiltration and activation. Polyamine–cGAMP interactions constitute a metabolic rheostat that spatiotemporally regulates 2′3′-cGAMP propagation and activity. This mechanism prevents aberrant STING hyperactivation while enabling optimal immune responses, highlighting polyamine homeostasis as a gatekeeper of tissue-level immunity.

## Results

### Spermine and spermidine selectively inhibit 2′3′-cGAMP propagation.

**Figure 1:**
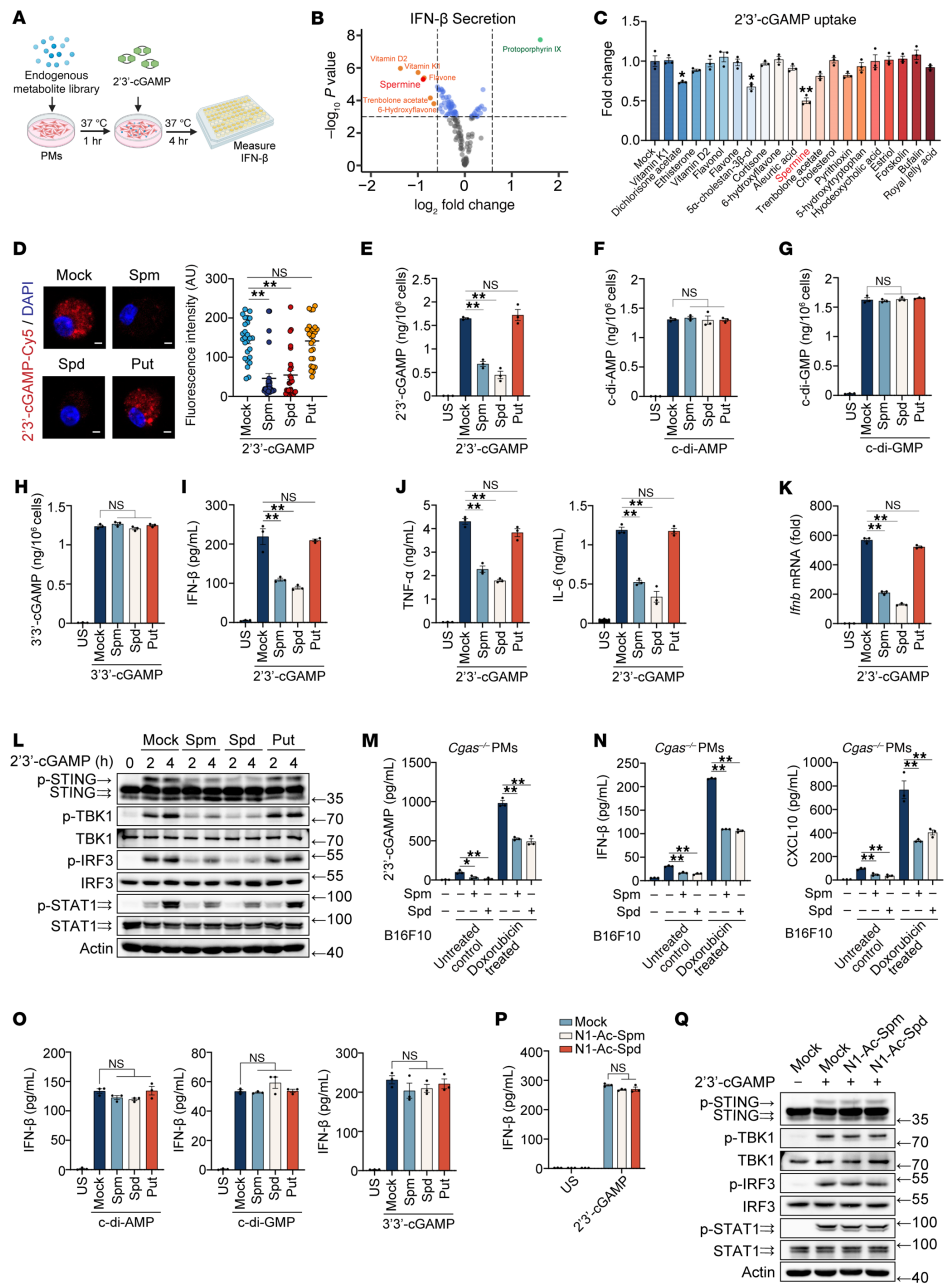
Spermine and spermidine selectively inhibit 2′3′-cGAMP propagation. (**A**) In vitro extracellular 2′3′-cGAMP–induced STING activation assay method. Images were created with BioRender. (**B**) In vitro STING activation assay measured as the amount of IFN-β secretion from PMs pretreated with a 10 μM endogenous metabolite library for 1 hour, followed by 2′3′-cGAMP stimulation as in (**A**). Fold changes were calculated as IFN-β production in metabolite-pretreated samples versus untreated controls upon 2′3′-cGAMP stimulation. The R package *Enhanced Volcano* was used to visualize endogenous metabolites regulating STING activation. (**C**) In vitro 2′3′-cGAMP uptake assay measuring intracellular 2′3′-cGAMP in PMs pretreated with the top 20 IFN-β secretion-inhibiting metabolites from (**B**), followed by 2′3′-cGAMP stimulation. Fold changes were calculated as 2′3′-cGAMP entrance in metabolite-pretreated samples versus untreated controls upon 2′3′-cGAMP stimulation. (**D**) Images and quantified fluorescence intensity of PMs pretreated with 10 μM polyamines for 1 hour and subsequently stimulated with 2′3′-cGAMP-Cy5 for 4 hours. Scale bars: 2 μm. (**E**–**L**) ELISA analysis of CDNs entry (**E**–**H**), cytokine expression (**I** and **J**), qPCR analysis of cytokines (**K**), or IB analysis of indicated antibodies (**L**) in PMs pretreated with polyamines, stimulated with CDNs. (**M** and **N**) ELISA analysis of 2′3′-cGAMP entry and cytokines expression in *Cgas*^–/–^ mouse PMs incubated with lysates from doxorubicin-treated B16F10 cells for 24 hours. (**O**–**Q**) ELISA analysis of IFN-β secretion (**O** and **P**) or IB analysis (**Q**) in mouse PMs pretreated with polyamines or *N*^1^-acetylpolyamines, stimulated with CDNs. Statistical significance was determined using unpaired 2-sided *t* test, and adjustments were made for multiple comparisons in **C**–**K** and **M**–**P**. The data are shown as the mean ± SEM. *P <0.05, **P <0.01. Similar results were obtained from 3 independent experiments. Put, putrescine; Spm, spermine; Spd, spermidine; US, unstimulated.

To identify metabolites that modulate extracellular 2′3′-cGAMP propagation and activity, we conducted a screening assay using an endogenous metabolite library. We pretreated mouse peritoneal macrophages (PMs) with individual metabolites and subsequently incubated them with 2′3′-cGAMP to model metabolite-mediated regulation of tissue microenvironment cGAMP-induced STING activation (Figure 1A). This screen identified several metabolites that suppressed extracellular 2′3′-cGAMP-triggered IFN-β secretion (Figure 1B and Supplemental Table 1; supplemental material available online with this article; https://doi.org/10.1172/JCI201460DS1). Among these, spermine, a polyamine, significantly impaired cellular uptake of extracellular 2′3′-cGAMP (Figure 1C). Given that mammalian cells produce 3 major polyamines (putrescine, spermidine, and spermine), none of which affected cellular pH (Supplemental Figure 1A), we systematically evaluated their impact on 2′3′-cGAMP uptake. Immunofluorescence and ELISA analyses revealed that spermine and spermidine, but not putrescine, specifically blocked 2′3′-cGAMP entry into cells (Figure 1, D and E). Notably, polyamines did not inhibit the cellular uptake of other CDNs, including cyclic di-AMP (c-di-AMP), cyclic di-GMP (c-di-GMP), and 3′3′-cGAMP (Figure 1, F–H), demonstrating selectivity for 2′3′-cGAMP.

Next, we assessed the functional consequences of polyamine-mediated 2′3′-cGAMP uptake inhibition on STING signaling. As a diffusible second messenger, 2′3′-cGAMP binds to STING on the ER membrane, triggering its dimerization, oligomerization, and Golgi translocation (1). This cascade activates TBK1-mediated phosphorylation of IRF3 and STAT1, culminating in type I IFN (IFN-I) and proinflammatory cytokine production (1). Consistent with impaired 2′3′-cGAMP entry, spermine and spermidine (but not putrescine) attenuated 2′3′-cGAMP–induced expression of IFN-β, TNF-α, and IL-6, and suppressed the phosphorylation of STING, TBK1, IRF3, and STAT1 (Figure 1, I–L, and Supplemental Figure 1B). To enhance the physiological relevance, we treated mouse B16F10 melanoma with doxorubicin, which cause mitochondrial DNA leakage, thereby activating cGAS and synthesizing 2′3′-cGAMP (13) (Supplemental Figure 1C). Then, as the endogenous cGAMP, B16F10 cell lysates were added to polyamines-pretreated *Cgas*-deficient mouse PMs. Consistently, spermine and spermidine inhibited the endogenous 2′3′-cGAMP entry into cells, and downstream IFN-β and CXCL10 secretion (Figure 1, M and N), indicated that spermine and spermidine also inhibit the uptake and activity of endogenous 2′3′-cGAMP. We also examined the effect of polyamines on tumor cells. Consistent with the findings in macrophages, spermine and spermidine inhibited 2′3′-cGAMP–induced IFN-β expression and the phosphorylation TBK1 and IRF3 in mouse MC38 colon cancer cells and HeLa cells (Supplemental Figure 1, D–G). Polyamines exerted no inhibitory effects on signaling activated by other CDNs (Figure 1O and Supplemental Figure 1, H–J).

*N*^1^-acetylspermine and *N*^1^-acetylspermidine are byproducts of polyamine catabolism mediated by SAT1. Neither of them affects cGAMP-induced IFN-β expression or and the phosphorylation of STING, TBK1, IRF3, and STAT1(Figure 1, P and Q, and Supplemental Figure 1K). Taken together, these results indicated that spermine and spermidine selectively restrict exogenous and endogenous 2′3′-cGAMP transmission to attenuate STING activation in immune and tumor cells.

### Spermine and spermidine selectively inhibit intracellular 2′3′-cGAMP–induced STING activation.

**Figure 2:**
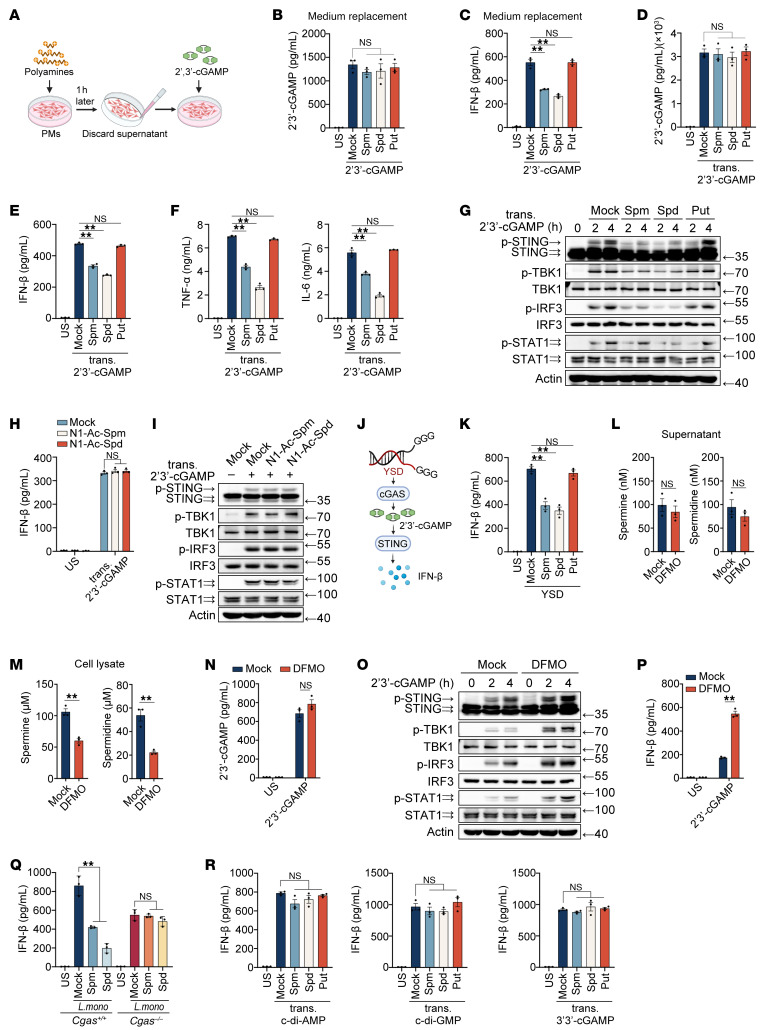
Spermine and spermidine selectively inhibit intracellular 2′3′-cGAMP-induced STING activation. (**A**) In vitro polyamines discard assay for assessing cGAMP channel dependence. Images were created with BioRender. (**B** and **C**) ELISA analysis of 2′3′-cGAMP entry (**B**) or IFN-β secretion (**C**) in mouse PMs pretreated with polyamines, followed by 2′3′-cGAMP stimulation as in (**A**). (**D**–**I**) ELISA analysis of 2′3′-cGAMP entry (**D**), cytokines secretion (**E**, **F**, and **H**), or IB analysis of indicated antibodies (**G** and **I**) in mouse PMs pretreated with polyamines (**D**–**G**) or *N*^1^-acetylpolyamines (**H** and **I**), transfected with 2′3′-cGAMP. (**J**) Schematic of YSD-mediated activation of the cGAS-STING pathway. Images were created with BioRender. (**K**) ELISA of IFN-β secretion in mouse PMs pretreated with polyamines, followed by 200 nM YSD for 4 hours. (**L** and **M**) Mass spectrometry quantification of spermine and spermidine in supernatant (**L**) and cell lysate (**M**) after 24 hours of 500 μM DFMO treatment. (**N**) ELISA analysis of 2′3′-cGAMP entrance in mouse PMs pretreated with DFMO, followed by stimulation with 2′3′-cGAMP. (**O** and **P**) IB analysis of indicated antibodies (**O**) or ELISA analysis of IFN-β secretion (**P**) in mouse PMs pretreated with DFMO, followed by stimulation with 2′3′-cGAMP. (**Q**) ELISA analysis of IFN-β secretion in *Cgas^+/+^* or *Cgas^–/–^* mouse PMs pretreated with polyamines, followed by infection with *Listeria* *monocytogenes*. (**R**) ELISA analysis of IFN-β secretion in mouse PMs pretreated with polyamines, followed by transfection with CDNs. Statistical significance was determined using an unpaired 2-sided *t* test, and adjustments were made for multiple comparisons in **B**–**F**, **H**, **K**–**N**, and **P**–**R**. The data are expressed as the mean ± SEM. *P <0.05, **P <0.01. Similar results were obtained from 3 independent experiments. Put, putrescine; Spm, spermine; Spd, spermidine; trans., transfection; US, unstimulated.

2′3′-cGAMP is internalized via membrane-spanning importer channels, enabling its intercellular propagation (3). To determine whether polyamines modulate 2′3′-cGAMP propagation by targeting these channels, we pretreated mouse PMs with polyamines for 1 hour, replaced the medium to remove free polyamines, and stimulated cells with 2′3′-cGAMP (Figure 2A). Notably, polyamines failed to suppress 2′3′-cGAMP cellular uptake under these conditions (Figure 2B), indicating that their inhibitory effects require direct interaction with extracellular 2′3′-cGAMP rather than modulation of membrane transporters.

Intriguingly, although medium replacement abolished polyamine-mediated suppression of cGAMP internalization, spermine and spermidine still attenuated 2′3′-cGAMP-induced IFN-β expression (Figure 2C). This suggested an additional intracellular mechanism targeting cytosolic cGAMP activity. To test this hypothesis, we transfected 2′3′-cGAMP into PMs via liposome delivery, ensuring unchanged intracellular cGAMP levels (Figure 2D). Spermine and spermidine, but not putrescine, *N*^1^-acetylspermine, or *N*^1^-acetylspermidine, markedly inhibited liposome-transfected 2′3′-cGAMP–induced expression of IFN-β, TNF-α, and IL-6, as well as the phosphorylation of STING, TBK1, IRF3, and STAT1 (Figure 2, E–I and Supplemental Figure 2, A and B), confirming their intracellular suppression of cGAMP-STING signaling. Also, spermine and spermidine inhibited liposome-transfected 2′3′-cGAMP–induced expression of IFN-β and the phosphorylation of TBK1 and IRF3 in MC38 and HeLa cells (Supplemental Figure 2, C–G).

Y-form DNA (YSD), a unique guanosine-flanked Y-form DNA, activates cGAS to generate 2′3′-cGAMP and trigger STING signaling (Figure 2J) (14). Although polyamines did not alter YSD-induced cGAMP production (15), they suppressed subsequent IFN-β secretion (Figure 2K). This contrasts with their known role in inhibiting cGAS via B-to-Z DNA transition (15). Critically, even when the B-to-Z transition was blocked by chloroquine (16) (eliminating polyamine effects on cGAS activation), spermine and spermidine still inhibited IFN-β production induced by chloroquine-pretreated polynucleotide poly(deoxyguanylic-deoxycytidylic) acid [poly(dG:dC)] (Supplemental Figure 2H), further supporting a 2′3′-cGAMP–specific intracellular mechanism. Difluoromethylornithine (DFMO), an ornithine decarboxylase inhibitor (17) (Supplemental Figure 2I), depleted intracellular polyamines without altering extracellular polyamine levels or 2′3′-cGAMP uptake (Figure 2, L–N). DFMO treatment attenuated STING activation triggered by both extracellular stimulation and intracellular transfection of 2′3′-cGAMP in PMs and MC38 (Figure 2, O and P, and Supplemental Figure 2, J–N), demonstrating that endogenous polyamines are crucial for intracellular cGAMP activity suppression.

Next, we examined whether polyamines could affect other CDN-induced STING activation. *Listeria monocytogenes* infection activates STING via both cGAS-dependent DNA sensing and direct c-di-AMP production (18). Spermine and spermidine suppressed infection-induced IFN-β in WT PMs rather than in *Cgas*-deficient cells (Figure 2Q), indicating no effect on c-di-AMP signaling. Consistently, spermine and spermidine did not inhibit cytokine expression or phosphorylation cascades induced by transfected c-di-AMP, c-di-GMP, or 3′3′-cGAMP (Figure 2R and Supplemental Figure 3, A–G). Additionally, DFMO treatment did not impact STING activation by these CDNs (Supplemental Figure 3, H–J). Overall, these results showed that spermine and spermidine specifically inhibited 2′3′-cGAMP–induced STING activation.

### Spermine and spermidine directly bind to 2′3′-cGAMP and promote its aggregation.

**Figure 3:**
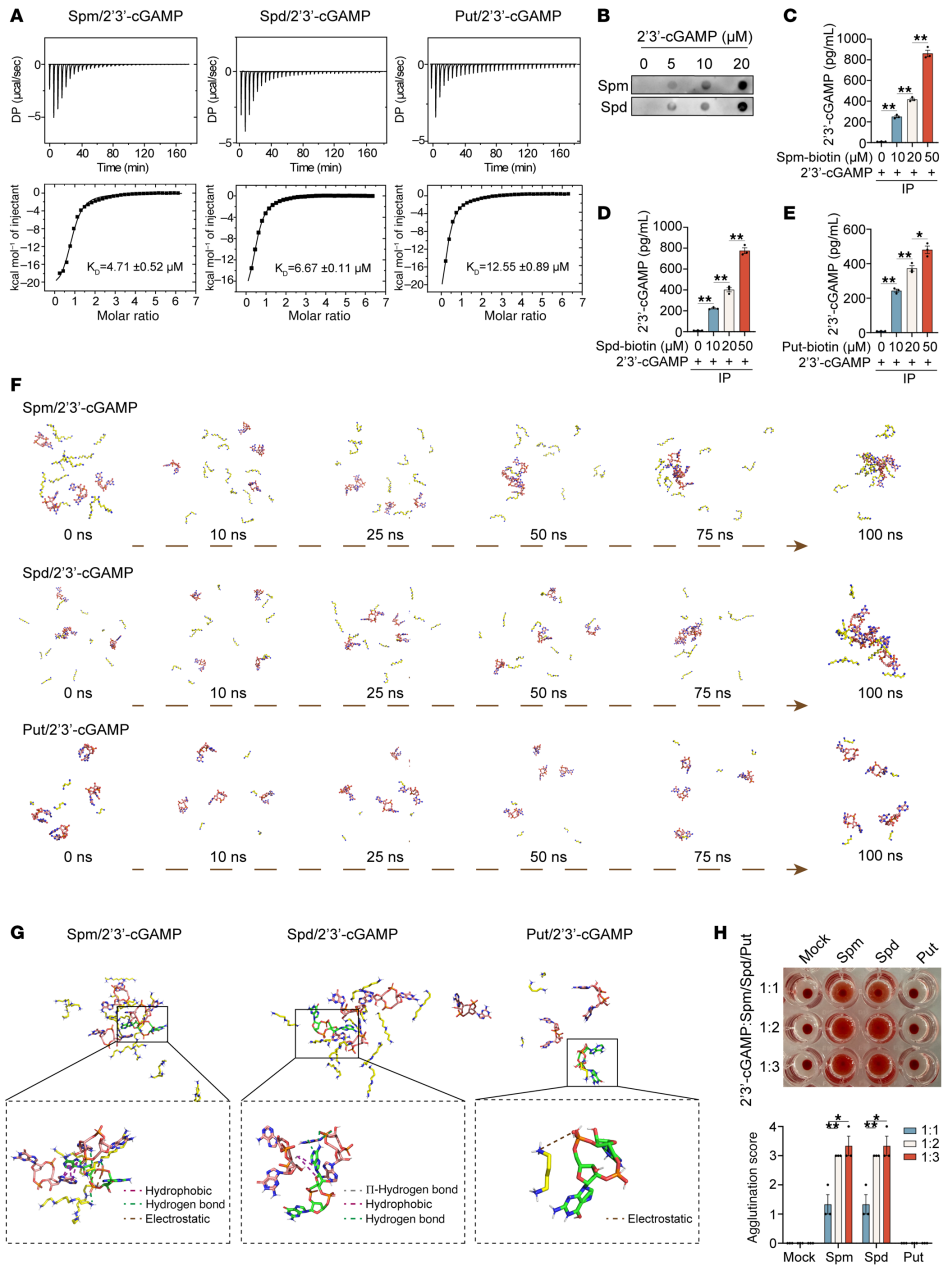
Spermine and spermidine directly bind to 2′3′-cGAMP and promote its aggregation. (**A**) The original titration traces (bar charts) and integrated data (line graphs) of ITC experiments, in which spermine (Spm), spermidine (Spd), or putrescine (Put) was titrated into a solution of 2′3′-cGAMP. DP, differential power. (**B**) Dot-blot analysis of spermine or spermidine binding to concentration-gradient 2′3′-cGAMP. (**C**–**E**) Spermine-, spermidine-, or putrescine-biotin incubated with an excess of 2′3′-cGAMP, followed by IP. ELISA analysis of bound 2′3′-cGAMP. (**F**) MD simulation snapshots of multivalent complex assembly between 2′3′-cGAMP and polyamines. (**G**) Binding mode analysis of 2′3′-cGAMP–polyamine interactions from MD simulations. (**H**) Images of indirect hemagglutination assay plates for detecting aggregates of 2′3′-cGAMP, and bar charts of polyamines and agglutination levels, scored based on passive hemagglutination assay. Statistical significance was determined using an unpaired 2-sided *t* test, and adjustments were made for multiple comparisons in **C**–**E** and **H**. The data are shown as the mean ± SEM. *P <0.05, **P <0.01. Similar results were obtained from 3 independent experiments.

To elucidate the molecular mechanism underlying polyamine-mediated suppression of 2′3′-cGAMP intercellular transfer and intracellular activity, we leveraged the polycationic nature of polyamines, which enables electrostatic interactions with negatively charged molecules, including CDNs and other second messengers (e.g., cAMP, cGMP, inositol-1,4,5-trisphosphate [IP3]). Isothermal titration calorimetry assays revealed that spermine, spermidine, and putrescine bound to 2′3′-cGAMP with dissociation constants in the micromolar range (Figure 3A), whereas no binding was observed with STING through co-immunoprecipitation (co-IP) assays (Supplemental Figure 4A). The specificity of spermine and spermidine–cGAMP interactions was further confirmed through dot blot and co-IP assays (Figure 3, B–E), indicating direct targeting of 2′3′-cGAMP by polyamines to control STING activation. Notably, spermine and spermidine also bound cAMP, cGMP, and IP3 (Supplemental Figure 4, B–D), suggesting broader roles in regulating second messenger–mediated signaling pathways.

Molecular dynamics (MD) simulations (GROMACS) revealed distinct binding modes between polyamines and 2′3′-cGAMP. Spermine and spermidine induced rapid formation of small cGAMP-polyamine clusters at 25 ns, which progressively coalesced into stable aggregates by 100 ns (Figure 3F). Structural analysis revealed multivalent interactions (hydrogen bonding and hydrophobic forces) anchoring cGAMP within polyamine-rich aggregates (Figure 3G). Molecular docking analysis showed that spermine and spermidine formed multivalent complexes via multiple binding sites, driving polymer-like aggregation, whereas putrescine engaged only a single binding site and so did not induce large-scale aggregation (Figure 3, F and G, and Supplemental Figure 4E). We used a passive hemagglutination assay crosslinking erythrocytes with 2′3′-cGAMP to validate aggregation in vitro. Spermine and spermidine (but not putrescine) triggered robust erythrocyte agglutination (Figure 3H and Supplemental Figure 4F), confirming the formation of polymer-like aggregate that may impede 2′3′-cGAMP propagation.

### Spermine and spermidine selectively suppress 2′3′-cGAMP binding to STING.

**Figure 4:**
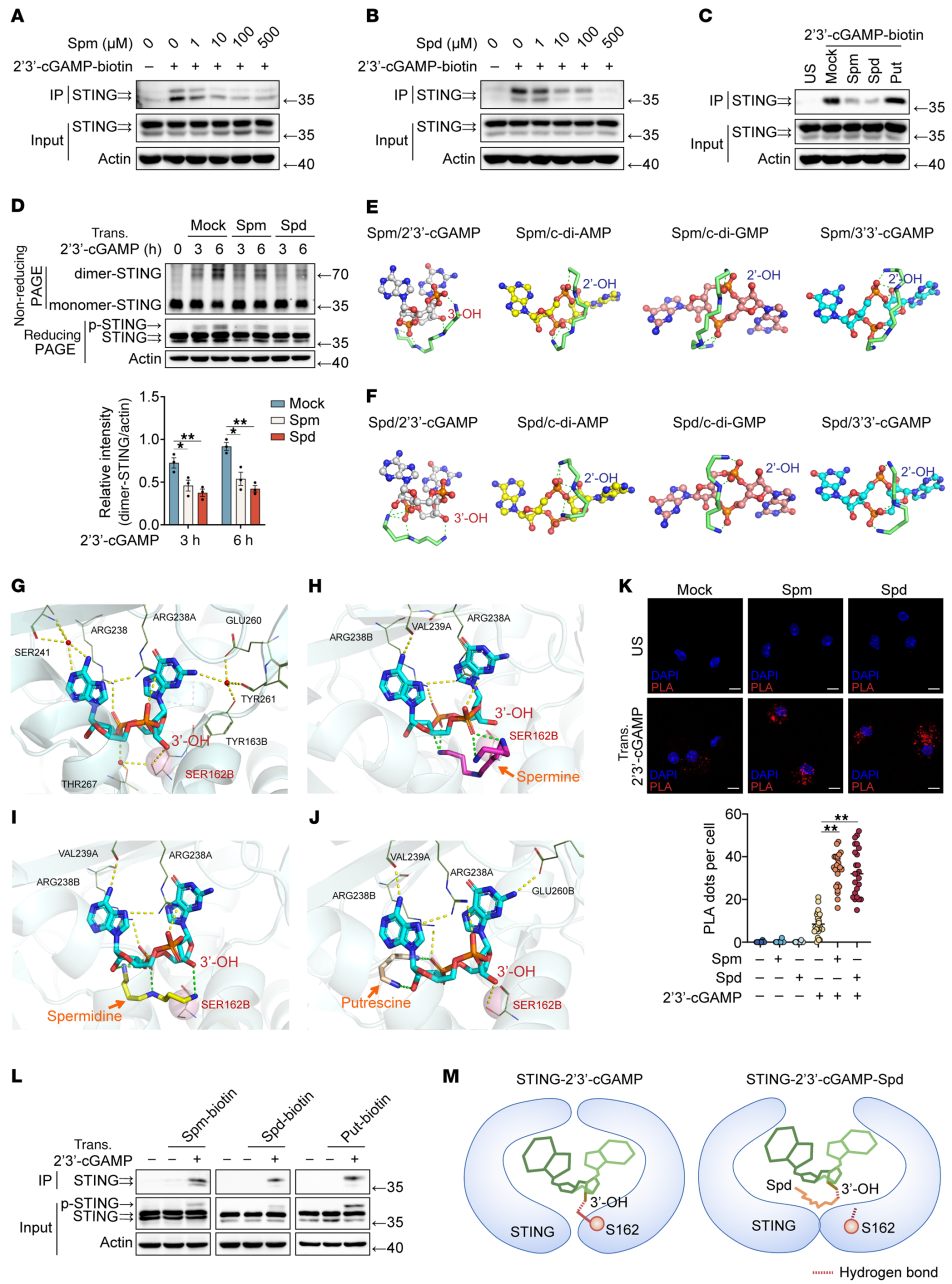
Spermine and spermidine selectively suppress 2′3′-cGAMP binding to STING. (**A**–**C**) IB analysis of lysates from mouse PMs were pretreated with different doses of spermine (**A**), spermidine (**B**), or 10 μM polyamines (**C**) for 1 hour, then incubated with 2′3′-cGAMP-biotin for 4 hours, followed by IP with streptavidin. (**D**) IB analysis of STING dimerization in mouse PMs pretreated with spermine or spermidine, followed by transfection with 2′3′-cGAMP. STING dimerization levels were quantitated by measuring dimer-STING band intensities using ImageJ software; the values were normalized to actin (bar chart). (**E** and **F**) Molecular docking analysis of spermine (**E**) or spermidine (**F**) interactions with 2′3′-cGAMP, c-di-AMP, c-di-GMP, or 3′3′-cGAMP. (**G**–**J**) Molecular docking of STING protein with 2′3′-cGAMP (**G**) supplemented with spermine (**H**), spermidine (**I**), and putrescine (**J**). (**K**) Representative confocal images of PLA between spermine or spermidine and STING in PMs pretreated with spermine or spermidine and transfected with 2′3′-cGAMP for 1 hour. The assembly of STING with polyamines was quantitated by the fluorescence intensity using ImageJ software (chart). Scale bars, 10 μm. (**L**) Lysates from mouse PMs pretreated with spermine-, spermidine-, or putrescine-biotin and transfected with 2′3′-cGAMP for 2 hours, followed by IP with streptavidin. IB analysis of STING expression. (**M**) Schematic of spermine and spermidine affecting the binding affinity between 2′3′-cGAMP and STING. Images were created with BioRender. Similar results were obtained from 3 independent experiments. Put, putrescine; Spm, spermine; Spd, spermidine; Trans., transfection; US, unstimulated.

Next, we explored the mechanism by which spermine and spermidine suppress intracellular 2′3′-cGAMP–induced STING activation. First, we confirmed that spermine and spermidine colocalized with 2′3′-cGAMP in mouse PMs (Supplemental Figure 5A). Spermine and spermidine dose-dependently inhibited 2′3′-cGAMP binding to STING (Figure 4, A–C, and Supplemental Figure 5B), consequently suppressing STING dimerization and phosphorylation (Figure 4D). Notably, although polyamines interacted with other CDNs, including c-di-AMP, c-di-GMP, and 3′3′-cGAMP (Supplemental Figure 5, C and D), they did not disrupt their binding to STING (Supplemental Figure 5, E–G).

The unique phosphodiester linkage architecture of 2′3′-cGAMP — comprising 1 bond between the 2′-OH of GMP and 5′-phosphate of AMP, and another between the 3′-OH of AMP and 5′-phosphate of GMP — exposes a free 3′-OH group on GMP, which is a distinctive structural signature absent in other CDNs (Supplemental Figure 6). Comparative structural analysis revealed fundamental differences in STING engagement (19, 20). c-di-AMP, c-di-GMP, and 3′3′-cGAMP primarily form hydrogen bonds via their ribose 2′-OH groups with Thr262 in mouse STING and Thr263 in human STING (19, 20). 2′3′-cGAMP establishes hydrogen bonds through its guanine base with Glu260, Thr263, and Val239 of STING, and its exposed 3′-OH group forms a critical hydrogen bond with Ser162, a key determinant of its higher binding affinity than other CDNs (12).

Molecular docking studies demonstrated that spermine and spermidine specifically engage the 3′-OH moiety of 2′3′-cGAMP via hydrogen bonding (Figure 4, E and F). Conversely, these polyamines only primarily form hydrogen bonds with phosphate groups of c-di-AMP, c-di-GMP, and 3′3′-cGAMP, with no direct hydrogen bonding observed to the 2′-hydroxyl groups (Figure 4, E and F). MD simulations further elucidated the allosteric inhibition mechanism. When spermine and spermidine bound to the 3′-OH group of 2′3′-cGAMP, they sterically obstructed the hydrogen-bond formation between 3′-OH and STING Ser162 (Figure 4, G–J). This disruption induced conformational destabilization of the STING-cGAMP complex, reducing binding stability. Putrescine, lacking multivalent binding capacity, did not perturb this interaction.

Functional validation via proximity ligation assay (PLA) confirmed that polyamines co-assembled with STING only upon 2′3′-cGAMP transfection, with no binding observed in the latter’s absence (Figure 4K). Co-IP assays corroborated the formation of a ternary STING-cGAMP-polyamine complex (Figure 4L). Collectively, these results establish a model wherein spermine and spermidine allosterically inhibit STING activation by competitively occupying the 3′-OH site of 2′3′-cGAMP, thereby weakening its binding affinity to STING and suppressing downstream signaling (Figure 4M).

### Spermine and spermidine effectively alleviate lupus-like phenotypes in Trex1^–/–^ mice.

*Trex1*-deficient mice develop severe systemic spontaneous inflammation, resulting in multiorgan dysfunctions, which can be ameliorated with the suppression of the cGAS-STING pathway and downstream related factors (21, 22). Spermine and spermidine reduced the expression of *Ifnb* and of chemokines *Ccl5* and *Cxcl10* in *Trex1*-deficient PMs (Supplemental Figure 7, A and B). To determine the physiological roles of polyamines in vivo, mice were orally administered spermine and spermidine for 4 weeks. Spermine and spermidine treatment significantly inhibited the serum levels of IFN-β, CCL5, and CXCL10, as well as the expression of *Ccl5, Isg15,* and *Ifit2* in the heart, brain, and kidney of *Trex1^–/–^* mice (Supplemental Figure 7, C–G). Moreover, *Trex1^–/–^* mice accumulated endogenous ssDNA and antinuclear antigens, which were substantially alleviated by spermine and spermidine treatment (Supplemental Figure 7, H and I). Long-term oral administration of spermine and spermidine also markedly prolonged the lifespan of *Trex1^–/–^* mice (Supplemental Figure 7J). These data demonstrate that polyamines inhibit STING activation in vivo and mitigate STING-driven autoimmune pathology.

### SAT1 enhances 2′3′-cGAMP–induced STING activation.

**Figure 5:**
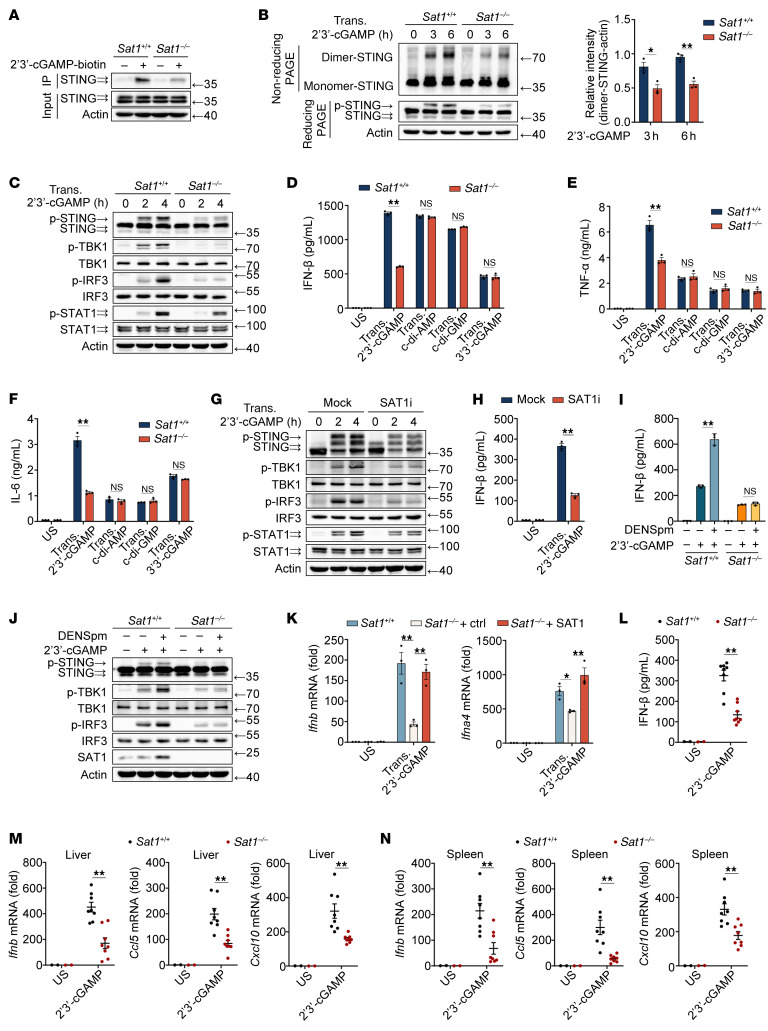
SAT1 enhances 2′3′-cGAMP–induced STING activation. (**A**) IB analysis of lysates from *Sat1^+/+^* or *Sat1^–/–^* mouse PMs incubated with 2′3′-cGAMP–biotin for 4 hours, followed by IP with streptavidin. (**B**) IB analysis of STING dimerization in *Sat1^+/+^* or *Sat1^–/–^* PMs stimulated by 2′3′-cGAMP. STING dimerization levels were quantitated by measuring dimer-STING band intensities using ImageJ software, and the values were normalized to actin (bar chart). (**C**) IB analysis of indicated proteins in *Sat1^+/+^* or *Sat1^–/–^* mouse PMs, followed by 2′3′-cGAMP transfection. (**D**–**F**) ELISA analysis of cytokines secretion in *Sat1^+/+^* or *Sat1^–/–^* PMs followed by CDN transfection (Trans.). (**G** and **H**) IB analysis of indicated proteins (**G**) or ELISA analysis of IFN-β secretion (**H**) in mouse PMs pretreated with 20 μM pentamidine for 24 hours, followed by 2′3′-cGAMP transfection. (**I** and **J**) ELISA analysis of IFN-β secretion (**I**) or IB analysis (**J**) in *Sat1^+/+^* or *Sat1^–/–^* PMs pretreated with 10 μM DENSpm for 24 hours, followed by 2′3′-cGAMP stimulation. (**K**) qPCR analysis of interferon expression from *Sat1^+/+^* MEFs transfected with an empty vector (*Sat1^+/+^*), *Sat1^–/–^* MEFs transfected with an empty vector (*Sat1^–/–^* +control [Ctrl]) or SAT1 plasmid (*Sat1^–/–^* +SAT1), followed by 2′3′-cGAMP transfection. (**L**–**N**) *Sat1^+/+^* or *Sat1^–/–^* mice were injected i.p. with 2′3′-cGAMP (50 μg per mouse). The serum cytokines were analyzed using ELISA after 2′3′-cGAMP injection for 2 hours (**L**). Gene expression in liver and spleen were analyzed using qPCR after 2′3′-cGAMP injection for 8 hours (**M** and **N**) (US, unstimulated; *n* = 2; 2′3′-cGAMP, *n* = 8 per condition). Statistical significance was determined using an unpaired 2-sided *t* test, and adjustments were made for multiple comparisons in **B**, **D**–**F**, **H**, **I**, and **K**–**N**. The data are shown as the mean ± SEM. *P <0.05, **P <0.01. Similar results were obtained from 3 independent experiments.

SAT1, the rate-limiting enzyme in polyamine catabolism, catalyzes the conversion of spermine and spermidine to putrescine, thereby depleting cellular pools of longer-chain polyamines (23). To define SAT1’s role in STING signaling, we analyzed PMs derived from *Sat1^–/–^* mice. SAT1 deficiency did not alter extracellular polyamine levels or impair 2′3′-cGAMP cellular internalization (Supplemental Figure 8, A and B). However, due to the elevated intracellular polyamine levels, it selectively suppressed 2′3′-cGAMP (but not c-di-AMP, c-di-GMP, or 3′3′-cGAMP) binding to STING (Figure 5A and Supplemental Figure 8, C–F). Consequently, SAT1 deficiency selectively attenuated 2′3′-cGAMP–induced expression of IFN-β and proinflammatory cytokines, STING dimerization and phosphorylation, and downstream phosphorylation of TBK1, IRF3, and STAT1 (Figure 5, B–F, and Supplemental Figure 8, G–N). Consistent with genetic ablation, the SAT1 inhibitor pentamidine or siRNA-mediated SAT1 knockdown similarly inhibited 2′3′-cGAMP–induced STING activation (Figure 5, G and H, and Supplemental Figure 9, A–E). Conversely, DENSpm (a potent SAT1 inducer that activates polyamine catabolism) enhanced 2′3′-cGAMP–driven STING signaling and cytokine production in WT PMs, rather than in *Sat1^–/–^* cells (Figure 5, I and J, and Supplemental Figure 9, F–I). Rescue experiments in *Sat1^–/–^* mouse embryonic fibroblasts (MEFs) transfected with SAT1-encoding plasmids restored 2′3′-cGAMP–induced expression of type I IFNs, TNF-α, and IL-6 (Figure 5K and Supplemental Figure 9, J and K). In vivo validation revealed that *Sat1^–/–^* mice had reduced serum levels of IFN-β, TNF-α, and IL-6 after 2′3′-cGAMP challenge (Figure 5L and Supplemental Figure 9L), and decreased *Ifnb, Ccl5,* and *Cxcl10* mRNA expression in the liver and spleen (Figure 5, M and N). Collectively, these data establish SAT1 as a selective enhancer of 2′3′-cGAMP-STING activation.

### Sat1 deficiency attenuates STING-driven antitumor immunity.

**Figure 6:**
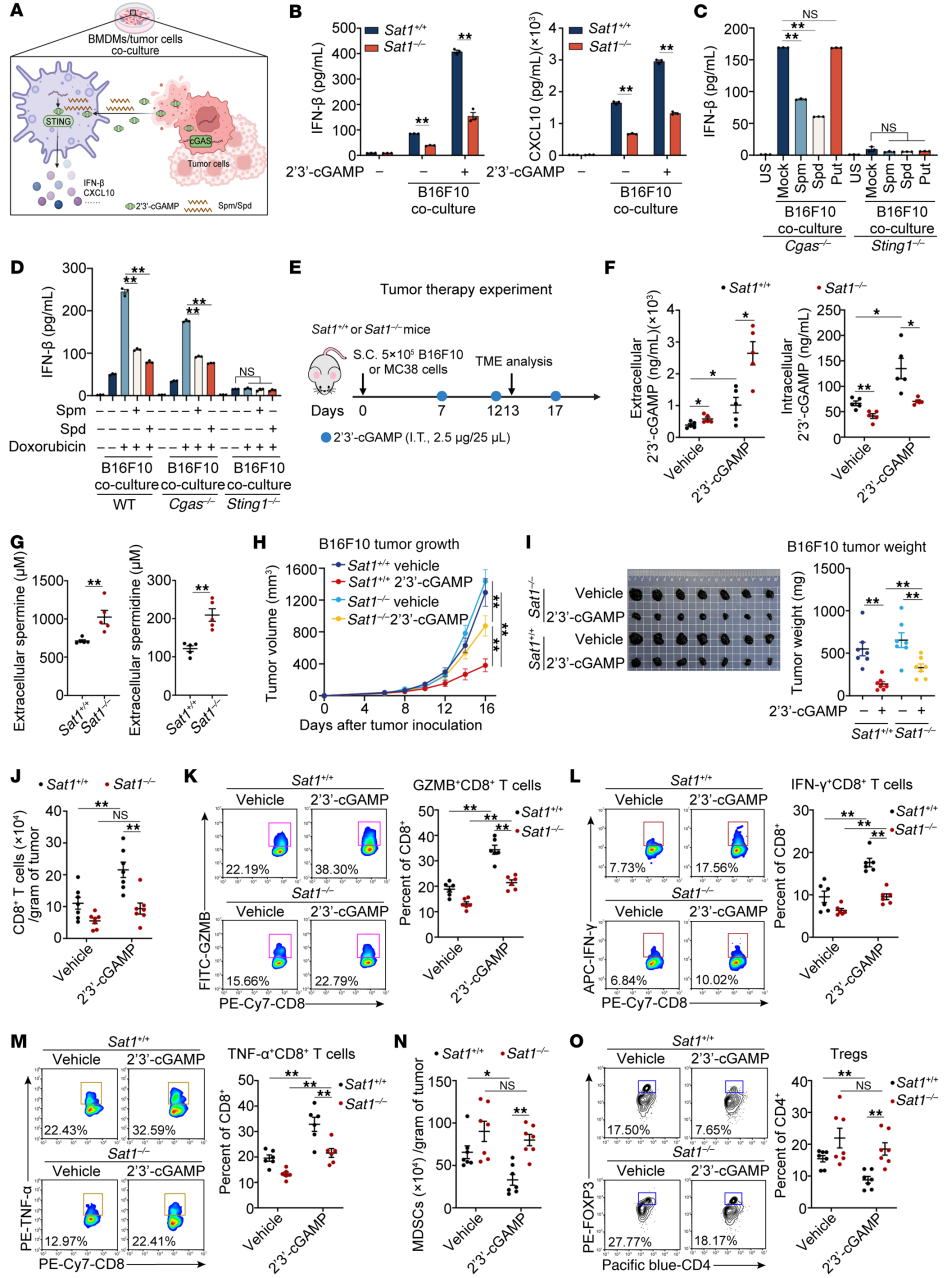
*Sat1* deficiency attenuates STING-driven antitumor immunity. (**A**) Schematic of the BMDM–tumor cell coculture system. Images were created with BioRender. (**B**) ELISA analysis of IFN-β and CXCL10 secretion in *Sat1^+/+^* or *Sat1^–/–^* BMDM coculture with B16F10, followed by 2′3′-cGAMP stimulation. (**C** and **D**) ELISA analysis in *Cgas^–/–^* or *Sting1^–/–^* BMDMs coculture with B16F10 pretreated with polyamines, then treated with doxorubicin or not. (**E**) Experimental scheme for *Sat1^+/+^* or *Sat1^–/–^* mice bearing subcutaneous tumors. (**F** and **G**) LC‑MS/MS, quantitative liquid chromatography‑tandem mass spectrometry quantification of extracellular 2′3′-cGAMP (**F**) or spermine and spermidine (**G**) in *Sat1^+/+^* or *Sat1^–/–^* mice at day 13 after inoculation with B16F10 (*n* = 5 per condition). (**H** and **I**) Tumor volume curve (**H**; *n* = 8 per condition) or tumor weight (**I**; *n* = 7 per condition) of *Sat1^+/+^* or *Sat1^–/–^* mice after B16F10 inoculation treated with 2′3′-cGAMP. (**L**–**O**) *Sat1^+/+^* or *Sat1^–/–^* mice at day 13 after inoculation with B16F10. Quantification of CD8^+^ T cell (**J**) or MDSC (**N**) populations per gram of tumor from tumor-bearing mice (*n* = 7 per condition). Flow cytometry analysis showing the percentage of intratumoral CD8^+^ T cells expressing GZMB (**K**), IFN-γ (**L**), and TNF-α (**M**) (*n* = 6 per condition) or CD4^+^FOXP3^+^ Tregs (**O**) (*n* = 7 per condition) in B16F10 tumor. Statistical significance was determined using an unpaired 2-sided *t* test, and adjustments were made for multiple comparisons in **B**–**D**, **F**, **G**, and **I**–**O** or 2-way ANOVA in **H**. The data are shown as the mean ± SEM. *P <0.05, **P <0.01. Similar results were obtained from 3 independent experiments. Put, putrescine; Spm, spermine; Spd, spermidine; I.T., intratumoral injection; APC, allophycocyanin; PE, phycoerythrin; FITC, fluorescein isothiocyanate.

Altered polyamine metabolism, characterized by elevated spermine and spermidine levels in malignant tumors, establishes an immunosuppressive tumor microenvironment (TME) (23). To assess the immunological relevance of SAT1, we analyzed the skin cutaneous melanoma (SKCM) cohort in The Cancer Genome Atlas (TCGA) dataset. CIBERSORT deconvolution revealed significantly enhanced infiltration of CD8^+^ T cells and M1 macrophages in tumors with high SAT1 expression (Supplemental Figure 10A). Consistently, high-SAT1 tumors exhibited enriched type I IFN signatures and elevated cytotoxic T lymphocyte (CTL) activity scores (Supplemental Figure 10B). To investigate the underlying mechanism, we established tumor cell–macrophage coculture systems using B16F10 melanoma or MC38 colon cancer with bone marrow–derived macrophages (BMDMs) (Figure 6A). Coculture of tumor cells/*Sat1*-deficient BMDMs elicited lower expression of IFN-β and CXCL10 than that of tumor cells/WT BMDMs, regardless of 2′3′-cGAMP stimulation (Figure 6B and Supplemental Figure 10C). Lower expression levels of IFN-β and CXCL10 were observed in both spermine- and spermidine-treated coculture of tumor cells/*Cgas*-deficient BMDMs (Figure 6C and Supplemental Figure 10, D and E). Notably, cytokine secretion in the coculture of tumor cells/*Sting1*-deficient BMDMs was markedly decreased, indicating that STING activation in macrophages is crucial for the expression of IFN-β and CXCL10. Additionally, spermine and spermidine did not affect the secretion of IFN-β and CXCL10 in the coculture of tumor cells/*Sting1*-deficient BMDMs (Figure 6C and Supplemental Figure 10, D and E).

Our previous study showed that spermine and spermidine induce the transition of B-form DNA to Z-form DNA, thereby decreasing cGAS activation (15). Less expression of IFN-β and CXCL10 was observed in a spermine or spermidine and doxorubicin combination–treated coculture of tumor cells with both WT and *Cgas*-deficient BMDMs (Figure 6D and Supplemental Figure 10, F and G), indicating that polyamines inhibited antitumor immunity by both targeting DNA and cGAMP under chemotherapy conditions. These data suggest that SAT1 and polyamines control antitumor immunity by impairing IFN-β and CXCL10 secretion in a STING-dependent manner.

Next, using WT and *Sat1*-deficient mice bearing subcutaneous B16F10 melanoma and MC38 colorectal carcinoma tumor grafts (Figure 6E), we evaluated the antitumor effect of SAT1. Elevated levels of spermine and spermidine in the TME of *Sat1*-deficient mice led to extensive extracellular accumulation of 2′3′-cGAMP and poor efficiency of 2′3′-cGAMP entrance (Figure 6, F and G). Therefore, after intratumoral administration of 2′3′-cGAMP, *Sat1-*deficient mice showed faster tumor progression than did WT mice (Figure 6, H and I, and Supplemental Figure 11, A and B). After 2′3′-cGAMP administration, intratumoral levels of IFN-β, CCL5, and CXCL10 in *Sat1-*deficient mice were considerably lower than those in WT mice (Supplemental Figure 11, C and D). The chemokines (e.g., CCL5, CXCL10) and type I IFNs are critical for CTL-mediated tumor-cell killing; they mediate T cell infiltration and drive robust Th1 and CTL responses, respectively. Concordantly, *Sat1*-deficient mice had less infiltration of total and activated CD8^+^ T cells, and an elevated proportion of CD11b^+^Gr-1^+^ myeloid-derived suppressor cells (MDSCs) and CD4^+^ FOXP3^+^ Treg cells (Figure 6, J–O). Collectively, these results indicate that SAT1 maintains polyamine catabolic homeostasis to potentiate cGAMP-STING signaling, thereby enabling type I IFN–dependent T cell recruitment and CTL-mediated antitumor immunity.

### DENSpm potentiates STING-dependent antitumor immunity via polyamine catabolic reprogramming.

**Figure 7:**
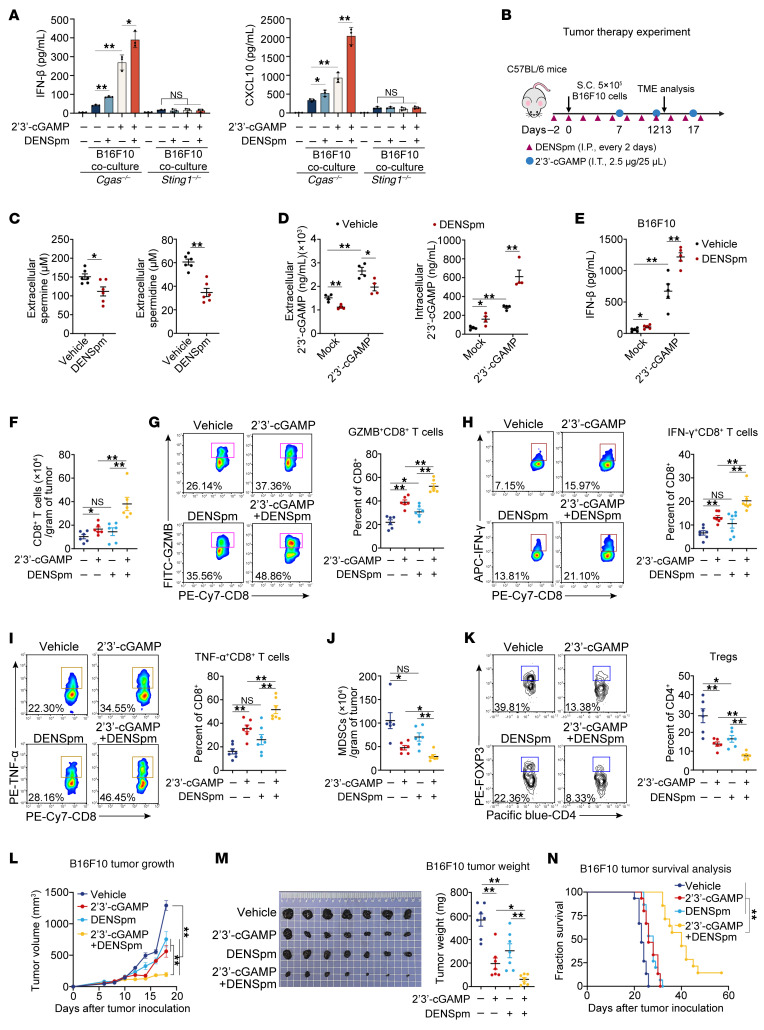
DENSpm potentiates antitumor immunity via polyamine catabolic reprogramming. (**A**) ELISA analysis of IFN-β and CXCL10 secretion in *Cgas^–/–^* or *Sting1^–/–^* BMDMs coculture with B16F10, pretreated with DENSpm, followed by 2′3′-cGAMP stimulation. (**B**) Experimental scheme for C57BL/6 mice bearing B16F10 subcutaneous tumors. (**C**–**K**) C57BL/6 mice at day 13 after B16F10 tumor inoculation treated with DENSpm/2′3′-cGAMP combination. Quantitative liquid chromatography‑tandem mass spectrometry (LC‑MS/MS) quantification of extracellular spermine and spermidine (**C**; *n* = 6 per condition) or 2′3′-cGAMP (**D**; *n* = 4 per condition). ELISA analysis of IFN-β secretion in tumor (**E**; *n* = 5 per condition). Quantification of CD8^+^ T cell (**F**) or MDSC (**J**) populations per gram tumor from tumor-bearing mice (*n* = 6 per condition). Flow cytometry analysis showing the percentage of intratumoral CD8^+^ T cells expressing GZMB (**G**; *n* = 6 per condition), IFN-γ (**H**; *n* = 7 per condition), and TNF-α (**I**; *n* = 7 per condition) or CD4^+^FOXP3^+^ Tregs (**K**; *n* = 6 per condition) in B16F10 tumor. (**L**–**N**) Tumor volume curve (**L**; *n* = 8 per condition), tumor weight (**M**; *n* = 7 per condition), or survival (**N**; *n* = 15 per condition) of C57BL/6 mice after B16F10 tumor inoculation treated with DENSpm/2′3′-cGAMP combination. Statistical significance was determined using an unpaired 2-sided *t* test and adjustments were made for multiple comparisons in **A**, **C**–**K**, and **M**, 2-way ANOVA in **L**, or the log-rank Mantel-Cox test in **N**. The data are shown as the mean ± SEM. *P <0.05, **P <0.01. Similar results were obtained from 3 independent experiments. I.T., intratumoral injection; APC, allophycocyanin; PE, phycoerythrin; FITC, fluorescein isothiocyanate.

We used the SAT1 stabilizer DENSpm to potentiate antitumor immunity through physiological polyamine metabolism restoration. DENSpm increases SAT1 expression and decreases serum concentrations of polyamines (15), and enhances 2′3′-cGAMP–induced IFN-β, TNF-α, and IL-6 secretion in vivo (Supplemental Figure 12A), indicating that DENSpm could augment STING activation in vivo. In tumor cell/macrophage cocultures using *Cgas^–/–^* BMDMs, DENSpm enhanced IFN-β and CXCL10 expression irrespective of cGAMP stimulation (Figure 7A and Supplemental Figure 12B). Critically, this enhancement was completely abolished in *Sting1^–/–^* BMDM cocultures, thus establishing STING dependence (Figure 7A and Supplemental Figure 12B). These findings demonstrate DENSpm-mediated reprogramming of an immunosuppressive TME through STING-dependent chemokine induction.

We used a subcutaneous B16 melanoma model to evaluate the antitumor efficacy of the SAT1 stabilizer DENSpm in vivo (Figure 7B). Intraperitoneal injection of DENSpm significantly reduced extracellular spermine and spermidine levels in the TME (Figure 7C), consistent with its role in activating polyamine catabolism. This metabolic reprogramming enhanced 2′3′-cGAMP bioavailability, as evidenced by reduced extracellular cGAMP accumulation, increased intracellular cGAMP retention, and potentiated secretion of IFN-β, CCL5, and CXCL10 in the tumor after cGAMP stimulation (Figure 7, D and E, and Supplemental Figure 12C). Synergistic DENSpm plus cGAMP administration profoundly remodeled the TME immunophenotype, driving enhanced infiltration and activation of CD8^+^ T cells while suppressing immunosuppressive populations, including MDSCs and Tregs (Figure 7, F–K). Notably, this regimen effectively converted immunologically “cold” tumors to immunologically active states. Concordantly, therapeutic outcomes demonstrated striking combinatorial synergy. Although monotherapies (DENSpm or cGAMP alone) moderately delayed tumor progression, combined treatment significantly suppressed tumor growth and extended survival in B16F10 melanoma–bearing mice (*P* < 0.001) (Figure 7, L–N). To determine whether CD8^+^ T cells were required for cGAMP-induced antitumor response, we treated mice with neutralizing antibodies against CD8 and found that CD8^+^ T cell depletion abrogated the antitumor efficacy of DENSpm plus cGAMP combination therapy (Supplemental Figure 12, D–H). These results suggest DENSpm plus cGAMP combination therapy inhibits tumor growth mainly dependent on CD8^+^ T cells. In triple-negative breast cancer (4T1) models (Supplemental Figure 13A) — a paradigm of therapy-resistant “cold” tumors — cGAMP monotherapy failed to activate antitumor immunity (Supplemental Figure 13, B–I). DENSpm plus cGAMP combination therapy rescued this deficit, inducing robust IFN-β/CCL5/CXCL10 secretion, promoting CD8^+^ T cell infiltration/activation, and facilitating tumor clearance (Supplemental Figure 13, B–I).

**Figure 8:**
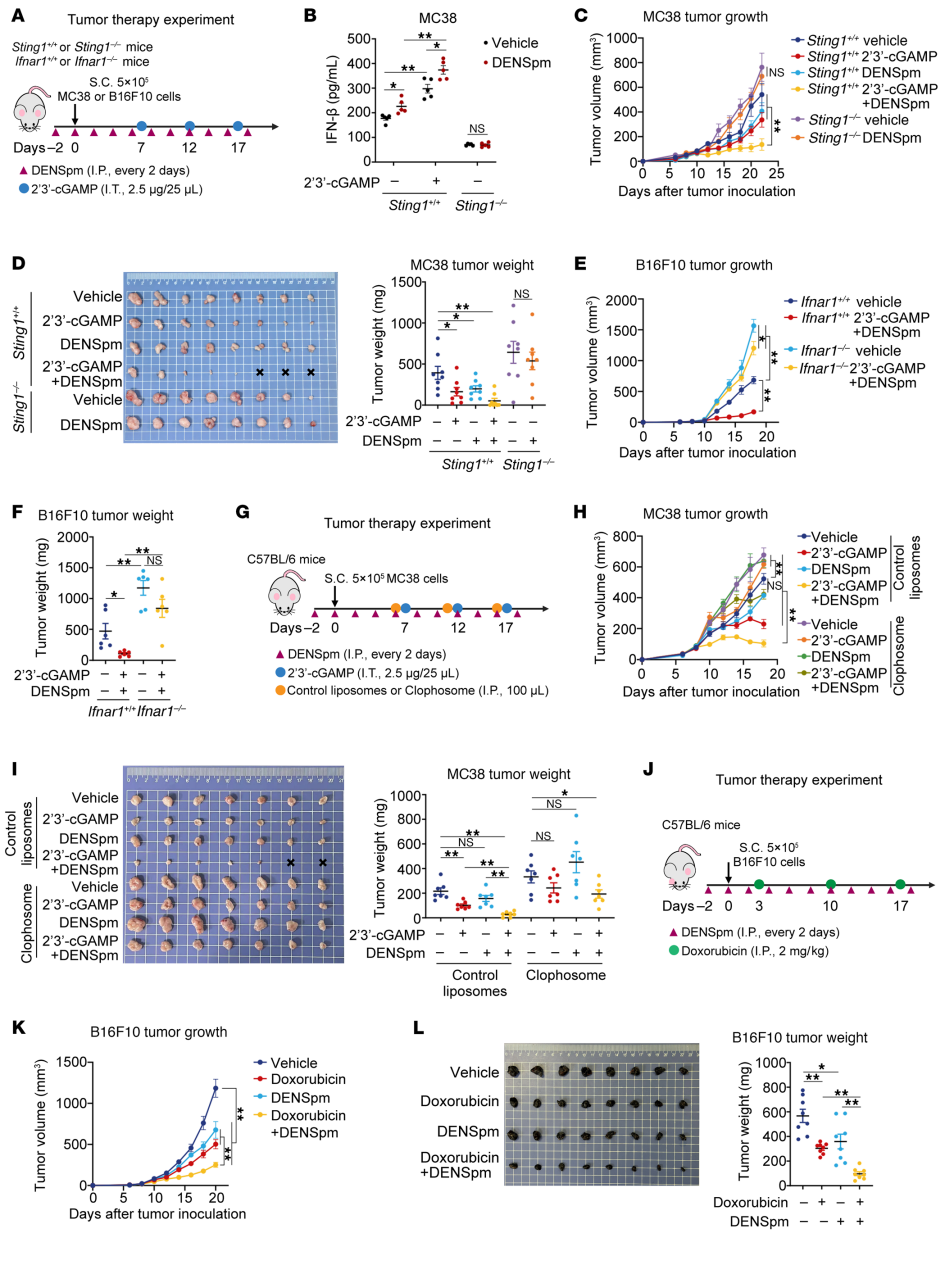
DENSpm potentiates antitumor immunity dependent on STING signaling. (**A**) Experimental scheme for *Sting^–/–^* or *Ifnar^–/–^* mice bearing subcutaneous tumors. (**B**) At day 13 after MC38 tumor inoculation, *Sting1^+/+^* mice treated with DENSpm/2′3′-cGAMP combination or *Sting1^–/–^* mice treated with DENSpm. ELISA analysis in tumor (*n* = 5/condition). (**C** and **D**) Tumor volume curve (**C**) and tumor weight (**D**) of *Sting1^+/+^* mice treated with DENSpm/2′3′-cGAMP combination or *Sting1^–/–^* mice treated with DENSpm after MC38 tumor inoculation (*n* = 8/condition). (**E** and **F**) Tumor volume curve (**E**) and tumor weight (**F**) of *Ifnar1^+/+^* or *Ifnar^–/–^* mice treated with DENSpm/2′3′-cGAMP combination after B16F10 tumor inoculation (*n* = 6/condition). (**G**) Experimental scheme for C57BL/6 mice bearing MC38 subcutaneous tumors treated with DENSpm, 2′3′-cGAMP and Clophosome. (**H** and **I**) Tumor volume curves (**H**; *n* = 8 per condition) and tumor weights (**I**; *n* = 7 per condition) of C57BL/6 mice after MC38 tumor inoculation treated with the combination of DENSpm/2′3′-cGAMP and either Clophosome or control liposomes. (**J**) Experimental scheme for C57BL/6 mice bearing B16F10 subcutaneous tumors treated with DENSpm/doxorubicin combination. (**K** and **L**) Tumor volume curves (**K**; *n* = 8 per condition), tumor weights (**L**; *n* = 8 per condition) of C57BL/6 mice after B16F10 tumor inoculation treated with the combination of DENSpm/doxorubicin. Statistical significance was determined using an unpaired 2-sided *t* test and adjustments were made for multiple comparisons in **B**, **D**, **F**, **I**, and **L** or 2-way ANOVA in **C**, **E**, **H**, and **K**. The data are shown as the mean ± SEM. *P <0.05, **P <0.01. Similar results were obtained from 3 independent experiments. I.T., intratumoral injection.

Critically, DENSpm did not augment antitumor immunity in *Sting1^–/–^* mice (Figure 8, A–D, and Supplemental Figure 14, A–E). Also, the antitumor effect of DENSpm plus cGAMP combination therapy was significantly impaired by *Ifnar* deficiency (Figure 8, E and F, and Supplemental Figure 14F), demonstrating that its therapeutic efficacy strictly depends on STING–IFN axis signaling. Furthermore, systemic macrophage depletion via i.p. injections of clodronate liposomes impaired the antitumor effects of both 2′3′-cGAMP monotherapy and its synergistic combination with DENSpm (Figure 8, G–I), indicating macrophages as indispensable effectors for STING-driven tumor clearance.

Given the rapid degradation and poor cellular permeability of 2′3′-cGAMP, we also evaluated the effects of polyamines on the synthetic CDN STING agonists 5,6-dimethylxanthenone-4-acetic acid (DMXAA) and CMA, and a non-nucleotide STING agonist, SR-717 (24, 25). Both spermine and spermidine inhibited the STING activation mediated by DMXAA, CMA, and SR-717 (Supplemental Figure 15, A–E), but SAT1 deficiency attenuated STING activation induced by these agonists (Supplemental Figure 15, F–J). Our results suggest that depleting polyamines, in combination with various STING-agonist therapies, holds promise as a broadly applicable strategy for antitumor immunotherapy.

Given that chemotherapies are important inducers of 2′3′-cGAMP, a compelling opportunity exists to test their potential synergy with DENSpm in tumor-bearing mouse models. We used a subcutaneous B16F10 melanoma model to evaluate the antitumor efficacy of the combination of doxorubicin chemotherapy and DENSpm in vivo (Figure 8J). Monotherapies (i.e., DENSpm or doxorubicin alone) moderately delayed tumor progression; however, combined treatment significantly suppressed tumor growth and extended survival in B16F10 melanoma–bearing mice (Figure 8, K and L), indicated striking combinatorial synergy. Furthermore, in evaluating the CA209-038 study dataset (26), we found that patients with a complete response or partial response to treatment with the anti–PD-1 agent nivolumab had a significantly lower score of polyamine anabolism (ornithine decarboxylase [ODC], spermidine synthase [SRM], and spermine synthase [SMS]) (Supplemental Figure 15K), suggesting that high levels of polyamines in the TME might lead to resistance to immune checkpoint inhibitors, in turn, highlighting the tremendous promise of DENSpm in combination with immune checkpoint blocker therapy.

Collectively, depleting immunosuppressive polyamines amplifies STING activation, which promotes macrophage-dependent chemokine secretion that recruits cytotoxic CD8^+^ T cells, thereby converting immunologically cold tumors to hot microenvironments and enabling tumor clearance. This activity demonstrates tremendous promise in tumor immunotherapy, including STING-agonist therapy, chemotherapy, and immune checkpoint blockade.

## Discussion

2′3′-cGAMP is a critical diffusible second messenger that amplifies STING-dependent immune responses in both autocrine and paracrine ways under diverse physiological and pathological conditions (27). In tumors, 2′3′-cGAMP is primarily generated by tumor cells through cGAS activation triggered by cytosolic DNA from chromosomal instability and mitochondrial DNA leakage, and immune cells (e.g., macrophages, dendritic cells) produce it upon phagocytosing tumor DNA (28). The transfer of 2′3′-cGAMP from tumor to immune cells broadens antitumor immunity in a DNA-sensing-independent background. Therefore, the spatial-temporal dynamics of cGAMP-STING signaling dictate immune outcomes in tumors. The regulatory mechanism of intracellular cGAMP-STING signaling is extensively characterized, including autophagy clearance of cytosolic DNA (29) and various posttranslational modifications of cGAS and STING (30); however, the spatiotemporal regulation of 2′3′-cGAMP dissemination across tissue microenvironments remains unknown. Here, we show that spermine and spermidine interact with 2′3′-cGAMP to form large polymer-like aggregates, thereby blocking the cellular uptake of 2′3′-cGAMP and restricting its propagation. Spermine and spermidine also selectively weaken 2′3′-cGAMP’s intracellular activity by attenuating its binding affinity with STING. Consequently, spermine and spermidine attenuate 2′3′-cGAMP-induced STING activation and ameliorate *Trex1*-deficiency–mediated autoimmune disorders in vivo. SAT1 deficiency inhibits STING-dependent antitumor immunity by elevating spermine and spermidine levels. Therefore, polyamine metabolism is a critical rheostat for cGAMP-STING signaling in both immune cells and tissue microenvironments, with profound implications for immunotherapy development across autoimmunity and cancer.

Polyamines (namely, putrescine, spermidine, and spermine) orchestrate immunometabolic responses by binding to target proteins, thereby fine-tuning cellular functions in immunity and diseases. Specifically, spermidine binds mitochondrial trifunctional protein to enhance fatty acid oxidation and ATP production in CD8^+^ T cells (31), and spermine directly binds to JAK1 to impair JAK1-cytokine receptor interaction, thereby suppressing IFN-I responses and attenuating autoimmune pathogenesis in systemic lupus erythematosus and psoriasis (32). Concurrently, spermidine acts as a substrate for deoxyhypusine synthase, catalyzing the hypusination of translation factor eIF5A and acetylhypusination of receptor-interacting serine/threonine-protein kinase 1 (33, 34). These modifications promote the synthesis of the autophagy transcription factor TFEB to reverse B cell senescence (33) and inhibit inflammation and cell death (34), respectively. The protonated amine groups of polyamines confer a strong multivalent cationic charge at physiological pH. This electrostatic property drives their interactions with negatively charged biomolecules, including nucleotides (DNA and RNA) and second messengers (e.g., cAMP, 2′3′-cGAMP). For instance, spermine and spermidine bind DNA to induce B-to-Z-DNA transitions, reducing cGAS affinity and attenuating antiviral immune responses (15). Critically, polyamines sequester 2′3′-cGAMP, blocking its intercellular transmission and suppressing STING engagement, thereby restricting tissue-wide immune amplification. Although interactions with other messengers (e.g., cAMP) require further validation, this ability to coordinate second-messenger hubs, particularly across organelles, positions polyamines as metabolic tuners of signaling landscapes, reprogramming immune responses via a previously underrecognized mechanism.

Polyamine metabolism is consistently dysregulated in tumors, promoting rapid cancer proliferation and establishing an immunosuppressive TME (35). Spermine promotes pro-tumor polarization of TAMs via TDG-mediated DNA demethylation (36). N1-Ac-Spd activated SRC signaling to induced CCL1 macrophage polarization, thus dampening immunotherapeutic efficacy (37). However, the role of spermine and spermidine in mediating antitumor immunity via the innate immune system remains unclear. Chronic inflammation leads to the accumulation of N1-Ac-Spd in the TME, which alters immune cell infiltration and represents a sustained immunosuppressive state. In contrast, our results indicate that spermine and spermidine exert their effects during the transient activation of intrinsic tumor immunity, such as with STING-agonist treatment or chemotherapy. Notably, whereas Liu et al. (37) observed increased CD8^+^ T cell infiltration in tumors formed by *Sat1*-silencing Hepa1–6 cells inoculated into the liver tissues of C57BL/6 mice, our experiments showed that when WT tumor cells were inoculated into *Sat1*-deficient mice, CD8^+^ T cell infiltration was slightly reduced. These contrasting findings suggest SAT1 in tumor cells and immune cells plays distinct roles in shaping the TME.

Although therapeutic strategies targeting polyamine dysregulation (e.g., SAT1 stabilizer DENSpm) or STING activation (e.g., natural CDNs) have shown preclinical promise, their clinical translation remains limited (38–41). DENSpm monotherapy exhibits insufficient efficacy in trials, whereas CDNs face challenges, including poor membrane permeability, rapid degradation by extracellular nucleases, and suppression via polyamine-mediated electrostatic sequestration in the TME. Diverse synthetic non-nucleotide STING agonists (e.g., DMXAA, vadimezan) demonstrate improved stability; however, their clinical efficacy remains limited, likely attributed to poor cross-species translatability and the human STING protein being highly polymorphic (25). Even engineered CDNs (e.g., phosphorothioate-modified CDNs ADU-S100) and combination therapies (e.g., ADU-S100 plus PD-1 antibody spartalizumab) also displayed poor therapeutic efficacy (42, 43), underscoring the need for novel approaches. Critically, our study demonstrates that co-administration of DENSpm and 2′3′-cGAMP synergistically disrupts immunosuppressive TMEs by exhausting physiological polyamines and enhancing STING activation, thereby markedly facilitating tumor clearance in vivo. Therefore, cotargeting dysregulated polyamine metabolism and fine-tuning STING activation kinetics represent promising avenues to overcome current barriers in immunotherapy, leveraging synergistic mechanisms to reprogram immunosuppressive TMEs and improve immunotherapy outcomes.

This study establishes that polyamines (spermine and spermidine) sequester 2′3′-cGAMP into polymer-like aggregates, blocking its intercellular propagation and suppressing STING activation by reducing ligand-receptor binding affinity. Polyamine–cGAMP interactions function as a tunable rheostat governing spatiotemporal control of STING signaling across tissues. Synergistic delivery of endogenous 2′3′-cGAMP with DENSpm overcomes extracellular trapping, rescues STING activation, and reprograms “old tumors to immunogenic hot states via enhanced CD8^+^ T cell infiltration. This strategy offers a cost-effective solution for natural CDN delivery, demonstrating superior activation efficacy compared with synthetic STING agonists while concurrently normalizing polyamine metabolism to enhance immune checkpoint blockade efficacy and treat cGAMP-related disorders.

## Methods

### Sex as a biological variable.

Our study examined male and female animals. Similar findings are reported for both sexes.

### Animal model.

C57BL/6 and BALB/c mice were obtained from the Vital River Laboratory Animal Technology Co. *Sat1^–/–^* (strain S-KO-04195), *Cgas^–/–^* (strain S-KO-05046), and *Ifnar^–/–^* (catalog C001268) mice were purchased from Cyagen. *Sting1^–/–^* (strain 025805) mice were obtained from The Jackson Laboratory. *Trex-1^–/–^* (strain T013987) mice were sourced from GemPharmatech. All mice (male and female) were 6–8 weeks old.

For polyamine administration experiments, *Trex1^+/+^ or Trex1^–/–^* mice received 3 mM spermine (85590; Sigma–Aldrich) and 3 mM spermidine (S2626; Sigma–Aldrich) dissolved in sterile drinking water, initiated at 3 weeks of age. Serum and tissue analyses were performed after 4 weeks of treatment. For survival studies, polyamine-supplemented water was administered continuously, and mice were monitored until reaching experimental endpoints. Mice received i.p. injection of 2′3′-cGAMP (50 μg/mouse; tlrl-nacga23, InvitroGen). Serum cytokine levels were measured 2 hours after injection, whereas tissue analyses were conducted 8 hours after 2′3′-cGAMP administration. In tumor challenge experiments, syngeneic mice were subcutaneously inoculated with 5 × 10^5^ B16F10 melanoma, 5 × 10^5^ MC38 colon cancer, or 1 × 10^6^ 4T1 breast cancer cells in 100 μL of PBS. The mice were randomized into different groups, ensuring that the tumor size was similar across all groups before initiating treatment. DENSpm (120 nmol/mouse; HY-13610A, MedChemExpress) was initiated 2 days before tumor inoculation and repeated every 2 days. 2′3′-cGAMP (2.5 μg/25 μL per dose) was administered via intratumoral injections on days 7, 12, and 17. Clophosome (F70101C; FormuMax) or control liposomes (F70101F; FormuMax) were i.p. administered to mice 1 day before cGAMP treatment. Doxorubicin (2 mg/kg) was administered via i.p. injection once weekly. Anti-mouse CD8α or IgG2α isotype control (100 μg) was administered via i.p. injection every 2 days, starting on day 5 after tumor inoculation.

Tumor length and width were measured on the indicated days, and tumor size was calculated as V = ½ (tumor length × tumor width^2^). The immunological profile of the TME was analyzed on day 13 after tumor inoculation. Mice were monitored twice a week and euthanized when the subcutaneous tumor diameter exceeded 20 mm.

### Cell culture.

To obtain mouse primary PMs, mice were injected i.p. with 3% Brewer’s thioglycolate, and peritoneal exudate cells were harvested 3 days later. Subsequently, peritoneal exudate cells were cultured for 2 hours, and nonadherent cells were removed. The remaining adherent monolayer cells were used as the PMs. MEFs were isolated from *Sat1*^+/+^ and *Sat1^–/–^* embryos (day 13.5). BMDMs were grown for 7–10 days in DMEM supplemented with macrophage colony-stimulating factor, 10% FBS (Gibco), 100 U/mL penicillin, and 100 μg/mL streptomycin. B16F10 melanoma (catalog TCM36), MC38 colon cancer (catalog TCM46), 4T1 breast cancer (catalog TCM32) cells were purchased from the Cell Bank, Chinese Academy of Sciences. HeLa cells were purchased from Ubigene Biosciences. PMs, MEFs, BMDMs, 4T1, and Hela cells were cultured with complete DMEM containing 10% FBS and antibiotics (100 μg/mL streptomycin and 100 units/mL penicillin; Gibco). MC38 and B16F10 cells were cultured in RPMI 1640 medium containing 10% FBS and antibiotics. All cells were cultured at 37°C in an atmosphere of 5% CO_2_.

### Reagents, plasmids, and antibodies.

Spermine (catalog 55513), spermidine (catalog 49761), putrescine (catalog 51799), CMA (catalog SML3185), and DFMO (catalog D193) were purchased from Sigma-Aldrich. DENSpm (catalog HY-13610A), doxorubicin (catalog HY-15142), and SR-717 (catalog HY-131454) were obtained from MedChemExpress. Chloroquine (catalog S6999) and DMXAA (catalog S1537) were sourced from Selleck Chemicals. 2′3′-cGAMP (catalog tlrl-nacga23), 3′3′-cGAMP (catalog tlrl-nacga), c-di-AMP (catalog tlrl-nacda), c-di-GMP (catalog tlrl-nacdg), poly(dG:dC) (catalog tlrl-pgcn), and G3-YSD (catalog tlrl-ydna) were purchased from InvivoGen. 2′3′-cGAMP-Cy5 was obtained from AAT Bioquest. N1-Ac-Spm (catalog T36416) and N1-Ac-Spd (catalog T37170) were obtained from TargetMol. pCDNA3.1-C-FLAG-SAT1 was purchased from Biosune Biotechnology. Anti-mouse CD8α and IgG2α isotype control were purchased from Bio X Cell. Anti–p-IRF-3 (Ser396; catalog 4947), anti-IRF3(D83B6; catalog 4302), anti-STING (catalog 50494), anti–p-STAT1 (Tyr701; catalog 9167), anti-STAT1 (catalog 9172), anti-TBK1 (catalog 3031S), and anti-SAT1 (catalog 61586) were sourced from Cell Signaling Technology. Anti-NAK/TBK1 (phosphoS172; catalog ab109272) and anti-spermine (catalog ab26975) antibodies were purchased from Abcam. Anti–mouse β-actin (catalog 66009-I-Ig) and anti-STING (1F1E1; catalog 66680-1-Ig) were obtained from Proteintech. Anti-TREX1 (C-11; catalog sc-133112) was sourced from Santa Cruz Biotechnology.

### Endogenous metabolite compound screening.

The metabolites from the Human Endogenous Metabolite Compound Library used for screening were purchased from TargetMol (catalog L2500; 2018). The library contains 132 endogenous metabolites that are produced and found in the body, and exert specific physiological functions and biological activities, including bioactive peptides, nucleosides and their derivatives, vitamins, hormones, and other endogenous metabolites. PMs were pretreated with 10 μM endogenous metabolite library for 1 hour, followed by 2′3′-cGAMP stimulation for 4 hours. The resulting supernatant was used to measure IFN-β concentrations using an ELISA kit (BioLegend). Fold changes were calculated using the IFN-β production of the endogenous metabolite library treated with 2′3′-cGAMP versus untreated 2′3′-cGAMP. The R package *EnhancedVolcano* was used to plot the effect of endogenous metabolites on STING activity.

Isothermal titration calorimetry. All dissociation constants of binding reactions were determined through isothermal titration calorimetry (ITC) using a MicroCal VP-ITC (Malvern). Spermine, spermidine, or putrescine (2 mM in the syringe, 35 × 5 μL injections) and 2′3′-cGAMP, 3′3′-cGAMP, c-di-AMP, c-di-GMP, cAMP, cGMP, IP_3_, or DAG (40 μM in the reaction cell) were operated with a reference power of 16 μCal/s, an initial delay of 60 seconds, a 300-second interval between injections, and a syringe stirring speed of 307 rpm. All titration experiments we describe were performed using identical procedures. Origin software was used for baseline correction, data integration, and fitting the data curves to a single-site binding model.

### Indirect hemagglutination test.

An appropriate number of sheep red blood cells (SRBCs) (S9450, Solarbio) was washed 3 times with PBS (centrifuging at 376*g* for 5 minutes per wash and discarding the supernatant after each centrifugation). The cells were resuspended in PBS to obtain a 4% (vol/vol) suspension. Next, 10 μM 2′3′-cGAMP was mixed with the SRBC suspension and incubated at 37°C for 30 minutes. After incubation, cells were washed 3 times with PBS to remove unbound 2′3′-cGAMP. In designated wells of a U-bottom 96-well microplate, 50 μL of 10, 20, or 30 μM spermine, spermidine, or putrescine were added, respectively, followed by 50 μL of the 2′3′-cGAMP–coated SRBC suspension. The contents were gently mixed and incubated at 37°C for 30 minutes before observing agglutination. The agglutination level was scored as no agglutination (i.e., SRBCs form a dense, sharp-edged button at the bottom of the well; score 0), weak agglutination (i.e., a small SRBC button in the center with slight agglutination in the surrounding area; score 1), moderate agglutination (i.e., a thin layer of agglutinated SRBCs with a small central button; score 2), strong agglutination (i.e., most SRBCs agglutinate into a membranous layer with a tiny amount of sediment at the edge; score 3), or complete agglutination (i.e., SRBCs spread evenly as a membranous layer at the well bottom with an irregular edge, no visible button; score 4).

### In situ PLA.

PMs were treated with spermine for 1 hour and transfected with cGAMP for 0.5 hour. The cells were fixed and permeabilized with 0.5% Triton-X 100 in PBS. Subsequently, the cells were blocked in 5% BSA for 1 hour and incubated with STING (Proteintech, 66680-1-Ig) and spermine (Abcam, ab101458) primary antibodies at 4°C overnight. PLA was performed using Duolink In Situ PLA Probe Anti-Mouse MINUS (Sigma-Aldrich, DUO92004) and Detection Reagents Red (Sigma-Aldrich, DUO92008), following the manufacturer’s instructions. The nuclei were stained with DAPI (Beyotime) and visualized using a Zeiss LSM980 confocal laser microscope provided by the Micro Characterization Facility of Shandong University.

### Dot blot analysis.

PVDF membranes (Millipore) were activated in methanol and then washed in ultrapure water. After air drying at room temperature, 2′3′-cGAMP at concentrations of 5, 10, or 20 μM was spotted onto the membrane. Next, the membranes were blocked in 5% BSA for 1 hour after incubation with 10 μM spermine or spermidine for 1 hour. After washing, membranes were incubated with anti-spermine antibody (Abcam) overnight at 4°C. Membranes were incubated with species-specific HRP-conjugated secondary antibody, following detection using chemiluminescent substrate and imaging.

### In vitro pull-down assay.

Biotin–2′3′-cGAMP, biotin–3′3′-cGAMP, biotin–c-di-AMP, and biotin–c-di-GMP used for the in vitro pull-down assay were purchased from Biolog. The cells were lysed in NP-40 lysis buffer. Lysates were incubated with biotin-CDNs at 4°C for 4 hours and incubated with streptavidin agarose resin beads (Thermo Fisher Scientific) at 4°C for another 2 hours. The beads were washed 5 times with lysis buffer and subsequently analyzed for binding of the CDNs to STING through Western blotting. To assess 2′3′-cGAMP-polyamine co-aggregation, biotin-spermine, biotin-spermidine, or biotin-putrescine (Ruixibio) were incubated with excess 2′3′-cGAMP in lysis buffer (30 minutes on ice). After 3 washes with lysis buffer, complexes were thermally dissociated (95°C, 10 minutes), and free 2′3′-cGAMP was quantified using ELISA.

### Coculture experiments.

Tumor cells were seeded in 24-well plates at a concentration of 5 × 10^5^ cells/well. Subsequently, BMDMs were added to the culture at a 5:1 ratio (BMDMs/tumor cells) or omitted. Then, 24 hours later, the supernatants from the coculture were collected for subsequent analysis. The production of chemokines and cytokines by BMDMs was analyzed using the ELISA described above.

### In vivo analysis.

For in vivo analysis, tumor-bearing mice were treated after tumor injection and euthanized on day 13. Tumor tissues were minced into 1–2 mm³ fragments and digested in 4 mL of enzyme solution (RPMI 1640 medium containing 2% FBS, 0.8 mg/mL collagenase I [17100017, Gibco], 0.8 mg/mL collagenase IV [17104019, Gibco], and 8 μg/mL DNase I [D8071-25, Solarbio]) with constant agitation at 37°C for 1 hour. The digested suspension was sequentially filtered through 70 μm nylon mesh to obtain single-cell suspensions. Processed samples underwent either (a) quantitative mass spectrometry profiling of cGAMP and polyamines or (b) flow cytometry analysis of tumor-infiltrating immune cell populations.

### Molecular docking.

Molecular docking of 2′3′-cGAMP, 3′3′-cGAMP, c-di-AMP, and c-di-GMP with polyamines was performed with the AutoDock 4.2.6 program. The initial structures of 4 compounds were prepared using AutoDockTools 1.5.6, generating pdbqt files for docking simulations. The 3D structure of the spermidine was downloaded from the PubChem database. The Molecular Orbital Package (MOPAC) program was subsequently used to optimize the structure and calculate the PM3 atomic charge. The structures of polyamines were also prepared using AutoDockTools 1.5.6, and the corresponding pdbqt file was generated for docking simulations. The docking boxes were wrapped around the entire compounds. The number of grid points in the *xyz* axes of the grid box was set to 50 × 50 × 50, the grid spacing was 0.375 Å, the number of genetic algorithm runs was set to 100, and the rest parameters were set to default. The structure with the lowest docking energy was subjected to energy minimization. The optimization processes were performed in 2 steps as follows: first, the steepest descent method optimization of 2,000 steps was conducted, then the structures were further optimized by 2,000 steps using the conjugate gradient method.

For molecular docking analysis of polyamines’ effects on cGAMP-STING interaction, the PDB structure 4KSY was preprocessed before docking (12). Specifically, ADFR software was used to protonate the protein receptor, remove crystallographic water molecules, and merge charges, thereby generating a receptor model suitable for docking. For the ligands, 2′3′-cGAMP and other candidate molecules were first protonated using PyMOL. Subsequently, their 3D structures were generated under physiological pH 7.4 conditions using SailVina, followed by energy minimization with the MMFF94 force field to ensure the ligands were in their most stable conformational states. Next, SailVina was used to define the docking grid box (44), thereby identifying the potential binding region of the receptor. Molecular docking simulations were then performed using AutoDock Vina with the Vina scoring function (45). To achieve sufficient conformational sampling, the docking parameter exhaustiveness was set to 200, and the top 20 docking poses ranked by docking scores were retained for further analysis. As an initial validation step, 2′3′-cGAMP was docked individually, and the resulting docking pose was compared with its position in the original complex. Next, in multiligand docking experiments, spermidine, spermine, and putrescine were introduced simultaneously with 2′3′-cGAMP into the docking system.

### MD simulation.

The chemical structures of spermine, spermidine, putrescine, and 2′3′-cGAMP were constructed using the ChemDraw software package, and their structural optimization was performed using Gaussian. Three complex systems were constructed using the insert-molecule module of GROMACS (https://www.gromacs.org/), with a box size of 4.5 × 4.5 × 4.5 Å. To verify the ability of polyamines to form clusters with 2′3′-cGAMP, we conducted 100-ns MD simulations on the 3 systems. The MD simulations were performed using the AMBER20 software package and ff16SB force field, with the solvent model using the TIP3P water model and box boundaries set at 4.5 Å (46, 47). Before the MD simulations, 2-step energy optimization was performed on the 3 systems as follows. First was solute constraint (constraint force constant of 2.09 × 10^5^ kJ·mol/nm^2^, followed by 5,000 steps of steepest descent and 5,000 steps of conjugate gradient optimization). The subsequent procedure involved an unconstrained steepest descent, followed by 5,000 steps of conjugate gradient optimization. The convergence criteria established a limit at an energy gradient of less than 4.182 × 10^–4^ kJ·mol/nm^2^. After energy optimization, the MD simulations comprised the following 2 steps. The first experiment involved 5 ns solute-constrained MD simulations, with a constraint force constant of 41.82 kJ·mol/nm^2^. These simulations involved a gradual increase in system temperature from 0 K to 300 K. In the second experiment, 95 ns unconstrained isothermal MD simulations were conducted, with bond lengths of hydrogen-containing atoms constrained using the SHAKE algorithm and a nonbonding interaction radius of 8 Å (48). The integration step size was set to 2 fs, with conformations sampled every 1 ps. This resulted in 10,000 conformations being collected for subsequent analysis.

### Bioinformatics analysis.

Cell-type identification by estimating relative subsets of RNA transcript (CIBERSORT) is a computational method used to translate a normally differentiated gene expression matrix into an infiltrating immune-cell proportion. Using the CIBERSORT.R script based on TCGA data (49), the comparison of the differential expression of 18 immune cells between the SAT1 group and controls was visualized using the box diagram. For single-sample gene set enrichment analysis (ssGSEA) analysis, gene expression data were acquired from the TCGA-SKCM cohort or from patients treated with nivolumab from the CA209-038 study dataset (26). The gene signatures “GOBP_TYPE_I_INTERFERON_ PRODUCTION,” “GOBP_T_CELL_MEDIATED_CYTOTOXICITY,” and “polyamine anabolism (including ODC, SRM, and SMS)” were used to derive ssGSEA scores. ssGSEA was performed using the GSVA package (50).

### Statistics.

All experiments were repeated at least thrice. Data are presented as the mean ± SEM. Statistical analysis was performed using GraphPad Prism 10. All quantitative measurements were tested for normal distribution. Comparisons between the 2 groups were performed using the Student’s *t* test (unpaired and 2-tailed). The *P* value was corrected for multiple comparisons using the Holm-Sidak method. Survival curves were estimated using the Kaplan-Meier method and compared using the log-rank test. The Wilcoxon test showed statistically significant differences in the immune cell subtypes between the 2 clusters. Two-way analysis of variance was applied to compare tumor growth curves.

### Study approval.

All animal experiments were performed in accordance with the National Institutes of Health Guide for the Care and Use of Laboratory Animals and approved by the Scientific Investigation Board of the School of Basic Medical Science, Shandong University, Jinan, Shandong Province, China (approval ECSBMSSDU2021-2-048).

### Data availability.

All data supporting the findings of this study are available within the article and supplemental material, with all data points in graphs reported in the Supporting Data Values file; and from the corresponding author upon reasonable request. See complete unedited blots in the supplemental material.

## Author contributions

WZ designed research studies and wrote the manuscript. YM, CZ, JG, and WW performed the majority of the experiments; YF and JZ analyzed the data. KZ, XM, ZY, MJ, YQ, and HS assisted contributed to the experiment process and provided technical assistance. CG provided valuable expertise and advice. WZ conceived the project and provided overall directions. Co-first authorship was determined by the independent and complementary contributions: YM led the in vitro and cellular work, while CZ led the animal studies and contributed to manuscript writing.

## Conflict of interest

The authors have declared that no conflict of interest exists.

## Funding support

- National Natural Science Foundation of China (grants 82125020 to WZ, 82321002 to CG, and 32470925 to CZ).
- Shandong Provincial Nature Science Foundation (grant ZR2023ZD57 to WZ).

## Supplementary Material

Supplemental data

Unedited blot and gel images

Supporting data values

## Version 1 06/01/2026

Electronic publication
