# Supplementary material for: Polyamine sequestration of 2′3′-cGAMP constrains intercellular transmission and STING engagement to subvert antitumor immunity
Source: J Clin Invest. 2026 Jun 1;136(11):e201460. doi: 10.1172/JCI201460 (PMC13221228; doi:10.1172/JCI201460)
Supplement: Supplemental data [file jci-136-201460-s231.pdf]

Supplementary information

Polyamine sequestration of 2'3'-cGAMP constrains intercellular transmission and STING engagement to subvert antitumor immunity

Yunjin Ma[1, 2, 6], Chunyuan Zhao[1 ,2, 6], Jiacheng Guo[1,2], Yue Fu[1,2], Wei Wang[1,2], Jiangong Zhang[1,2], Kun Zhao[3], Xiangbo Meng[4], Zhongshang Yuan[5], Chengjiang Gao[1], Mutian Jia[1], Ying Qin[1,2], Hui Song[1,2], and Wei Zhao[1,2, *]

[1]Key Laboratory of Infection and Immunity of Shandong Province, and Key Laboratory for Experimental Teratology of the Chinese Ministry of Education, School of Basic Medical Science, Cheeloo College of Medicine, Shandong University, Jinan, Shandong, China

[2] State Key Laboratory for Innovation and Transformation of Luobing Theory; Key Laboratory of Cardiovascular Remodeling and Function Research of MOE, NHC, CAMS and Shandong Province; Department of Cardiology, Qilu Hospital of Shandong University, Jinan, China

[3]School of Pharmaceutical Sciences, Cheeloo College of Medicine, Shandong University, Jinan, Shandong, China

[4] Advanced Medical Research Institute, Meili Lake Translational Research Park, Cheeloo College of Medicine, Shandong University, Jinan, Shandong, 250012, China.

[5]Department of Biostatistics, School of Public Health, Cheeloo College of Medicine, Shandong University, 250012, Jinan, Shandong, China

[6]These authors contributed equally

*Correspondence to Wei Zhao: wzhao@sdu.edu.cn

Supplemental Methods

Measuring intracellular pH value

The intracellular pH value of PMs was measured using the pHrodo™ Green AM intracellular pH indicator, a fluorogenic probe, along with the intracellular pH calibration buffer kit, following the manufacturer's protocol (Cat# P35373, ThermoFisher Scientific). Briefly, cells were washed with live cell imaging solution (LCIS) and then incubated at 37 °C for 30 minutes with amixture of 10 μL pHrodo™ Green AM, 100 μL PowerLoad™ and 10 mL LCIS, followed by pH measurement using a spectrometer.

Plasmid transfection and RNA interference

The SAT1 expression plasmid was generated via polymerase chain reaction (PCR) amplification of cDNA derived from PMs and subsequently cloned into the pcDNA3.1-Flag eukaryotic expression vector. Successful plasmid construction was verified through DNA sequencing. Lipofectamine 2000 reagent (Invitrogen, San Diego, CA) was employed for transient plasmid transfection into MEFs. Regarding transient RNA interference, siRNA duplexes targeting mouse *Sat1* (sequences listed in Supplemental Table 2) were transfected into primary PMs using INTERFERin reagent (Polyplus-transfection) according to the manufacturer's protocols.

Protein extraction and immunoblotting

Cells were lysed in radioimmunoprecipitation assay (RIPA) buffer (Pierce, Thermo Fisher Scientific) supplemented with protease and phosphatase inhibitor cocktails (Sigma). Protein concentrations were quantified using the Pierce BCA Protein Assay Kit (Thermo Fisher Scientific). The cell lysates were boiled with the loading buffer and separated by sodium dodecyl sulfate–polyacrylamide gel electrophoresis (SDS-PAGE) gel electrophoresis. For non-reducing PAGE, the cell lysates were mixed

with loading buffer without boiling. Before SDS-PAGE, the gel was pre-run for 1 h. Electrophoresis was performed on ice under low voltage. Subsequently, the proteins were transferred onto polyvinylidene difluoride (PVDF) membranes (Millipore, Burlington, MA) for immunoblot analysis. Immunoblot signals were normalized to β-actin expression.

ELISA

ELISA kits were sourced as follows: IFN-β from BioLegend (San Diego, CA); TNF-α and IL-6 from Dakewe Biotech (Shenzhen, China); CXCL10 and CCL5 from Assay Genie (Dublin, Ireland); anti-ssDNA IgG and anti-ANA IgG from Alpha Diagnostic International (San Antonio, TX); and 2'3'-cGAMP, 3'3'-cGAMP, c-di-AMP, and c-di-GMP from Cayman Chemical (Ann Arbor, MI) to quantify the respective cytokines and compounds. ELISA detection was performed using cell supernatants, mouse serum, or mouse tumor. Mouse serum was obtained via retro-orbital bleeding under isoflurane anesthesia. For tumor lysate preparation, approximately 100 μg tissue was homogenized in ice-cold RIPA buffer (200 μl, Pierce) containing protease and phosphatase inhibitor cocktails (Sigma). Lysate supernatants were collected after centrifugation.

Quantitative real-time PCR (qPCR)

Total RNA was extracted using the RNA fast200 Extraction Kit (Fastagen, Shanghai) according to the manufacturer's protocols. cDNA was synthesized with reverse transcriptase (Vazyme Biotech), followed by qPCR analysis on an Applied Biosystems StepOnePlus Real-Time PCR system using ChamQ Universal SYBR Master Mix (Vazyme Biotech). Primer sequences are listed in Supplemental Table 2. All data were normalized to β-actin expression.

Immunofluorescence staining and confocal microscopy

PMs were plated on glass coverslips in 24-well plates, treated with polyamines, and either stimulated

or transfected with 2’3’-cGAMP-Cy5 (AAT Bioquest). The cells were fixed and permeabilized with 0.5% Triton-X 100 in PBS. Subsequently, the cells were blocked in 5% bovine serum albumin (BSA) for 1 h, then directly stained with DAPI (Beyotime, Shanghai, China) or incubated with primary antibodies (Spermine/Spermidine) at 4°C overnight. Next, the cells were stained with secondary antibodies (Alexa Fluor 488, Invitrogen), and the nuclei were stained with DAPI. Image analysis was performed using a Zeiss LSM980 confocal microscope at the Micro Characterization Facility of Shandong University. The fluorescence intensity was quantified using ImageJ software (NIH, Bethesda, Maryland, http://rsb.info.nih.gov/ij/, 1997–2006).

Flow cytometry analysis

The following monoclonal antibodies recognizing the indicated antigens were used: CD45 (30-F11) and CD4 (GK1.5) from eBioscience; and CD8 (53-6.7), CD11b (M1/70), TNF-a (MP6-XT22), FOXP3 (MF-14), GZMB (QA16A02), Gr-1 (RB6-8C5), and IFN-γ (XMG1.2) from BioLegend. Cells were washed with FACS buffer (PBS with 2% FBS and 2 mM EDTA) and incubated with Fc block (CD16/32, 2.4G2, BD Biosciences) for 10 min at 4°C before proceeding with antibody-mediated staining. Zombie Yellow™ Fixable Viability Kit (423104, BioLegend) was used to exclude dead cells. Regarding cell surface staining, 2×10^5 cell pellets were stained with appropriately diluted antibodies for 30 min at 4°C. Cells were washed with PBS containing 2% FBS and analyzed using flow cytometry. For the *ex vivo* analysis of T cells, cells were cultured for 4 h in the presence of phorbol myristate acetate (50 ng/ml, Sigma–Aldrich), ionomycin (500 ng/ml, MedChemExpress), and 1x brefeldin A (eBioscience). Cells were fixed and permeabilized using the FOXP3/Transcription Factor Staining Buffer Set (00-5523-00, eBioscience) according to the manufacturer’s protocols. For intracellular staining, samples were stained with permeabilization buffer using the corresponding

antibodies. Samples were acquired on a Novocyte Quanteon (Agilent). Data were analyzed using NovoExpress software. The strategies for T cell gating are shown in Supplemental Figure 16, A and C, respectively. The strategy for MDSC gating is presented in Supplemental Figure 16B.

Quantitative liquid chromatography-tandem mass spectrometry (LC-MS/MS)

Extracellular and intracellular polyamines or 2'3'-cGAMP were quantified using LC-MS/MS. Regarding cellular spermidine/spermine quantification, 1×10^6 cells were lysed in 1 mL of pre-chilled methanol/acetonitrile/water (2:2:1, v/v/v) and sonicated at 4°C for 30 min. For cell supernatants or tissue-equivalent tumor digest supernatant, 200 μL samples were mixed with 0.8 mL of pre-chilled methanol/acetonitrile (1:1, v/v), incubated at −20°C for 2 h. Next, the supernatant was centrifuged, vacuum-dried, reconstituted in 200 μL of 50% methanol, and filtered (0.22 μm). For 2'3'-cGAMP quantification, samples were mixed with methanol and processed identically before LC-MS/MS. Chromatographic separation was performed using a Shimadzu Nexera X2 LC-30AD ultra-high-performance liquid chromatography system. Mass spectrometric analysis was conducted using a 5500 QTRAP® mass spectrometer (AB Sciex) operating in positive and negative ion modes for spermidine/spermine and 2'3'-cGAMP detections, respectively.

Supplemental Figure 1

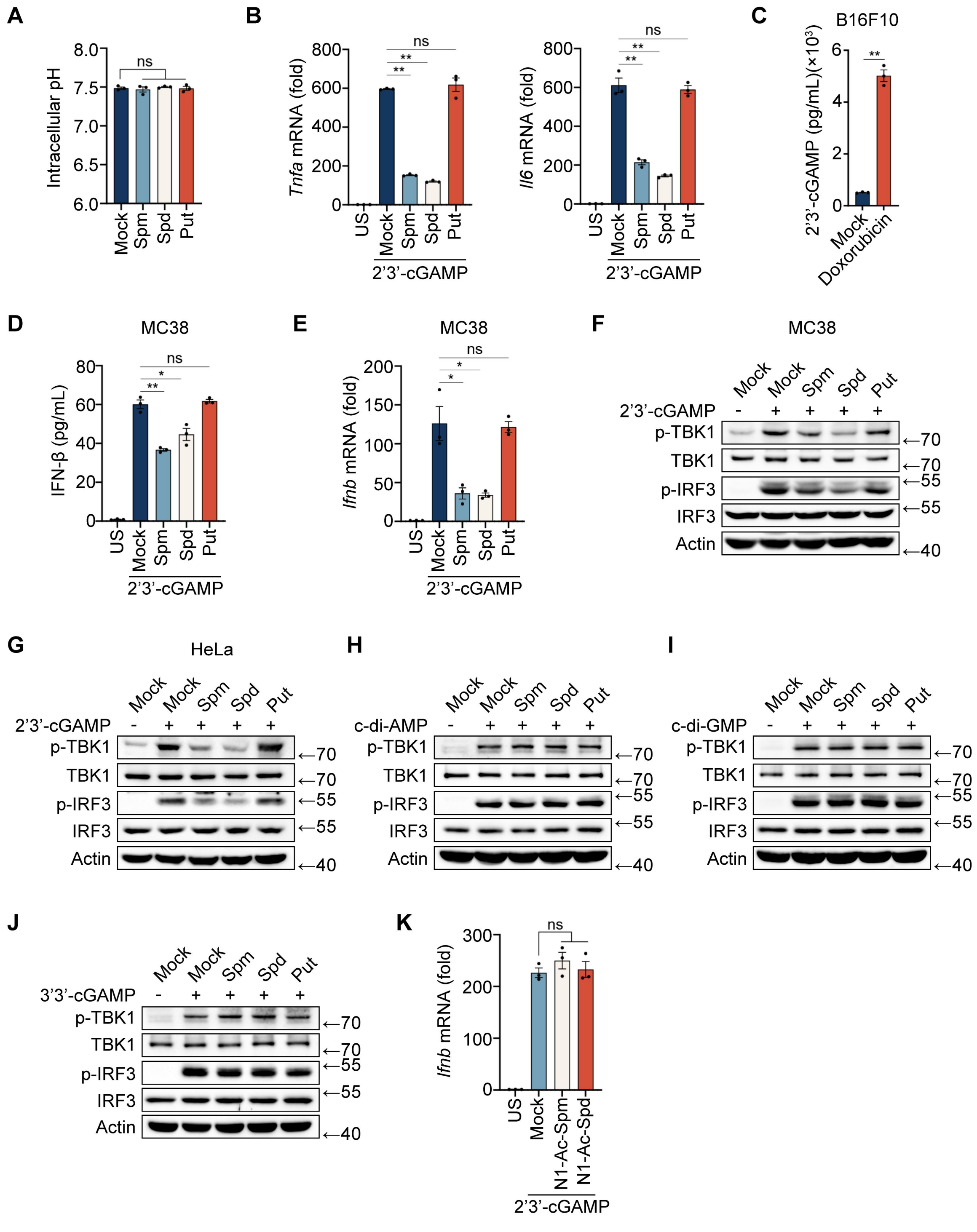

Supplementary Figure 1. Spermine and spermidine selectively inhibit 2'3'-cGAMP propagation

(**A**) PMs were treated with 10 µM polyamines for 1 h followed by measurement of cytosolic pH values.

(**B**) Q-PCR analysis of *Tnfa* and *Il6* expression in mouse PMs pretreated with polyamines and treated

with 2’3’-cGAMP for 4 h.

(**C**) ELISA analysis of 2’3’-cGAMP in B16F10 cells pretreated with 1 μM Doxorubicin for 24 h.

(**D** and **E**) ELISA (**D**) and qPCR (**E**) analysis of IFN-β expression in MC38 cells pretreated with polyamines, stimulated with 2’3’-cGAMP.

(**F** and **G**) Immuno blot analysis of indicated antibodies in MC38 (**F**) and Hela cells (**G**) pretreated with polyamines for and treated with 2’3’-cGAMP.

(**H**–**J**) Immuno blot analysis of indicated antibodies in mouse PMs pretreated with polyamines and treated with c-di-AMP (**H**), c-di-GMP (**I**), or 3’3’-cGAMP (**J**).

(**K**) QPCR analysis of *Ifnb* expression in PMs pretreated with 10 μM N1-Acetylspermine (N1-Ac-Spm) or N1-Acetylspermidine (N1-Ac-Spd) and treated with 2’3’-cGAMP.

Statistical significance was determined using unpaired two-sided t test and adjustments were made for multiple comparisons in **A**–**E**, and **K**. The data are shown as the mean ± SEM. *P <0.05, **P <0.01, and ns, not significant. Similar results were obtained from three independent experiments.

Supplemental Figure 2

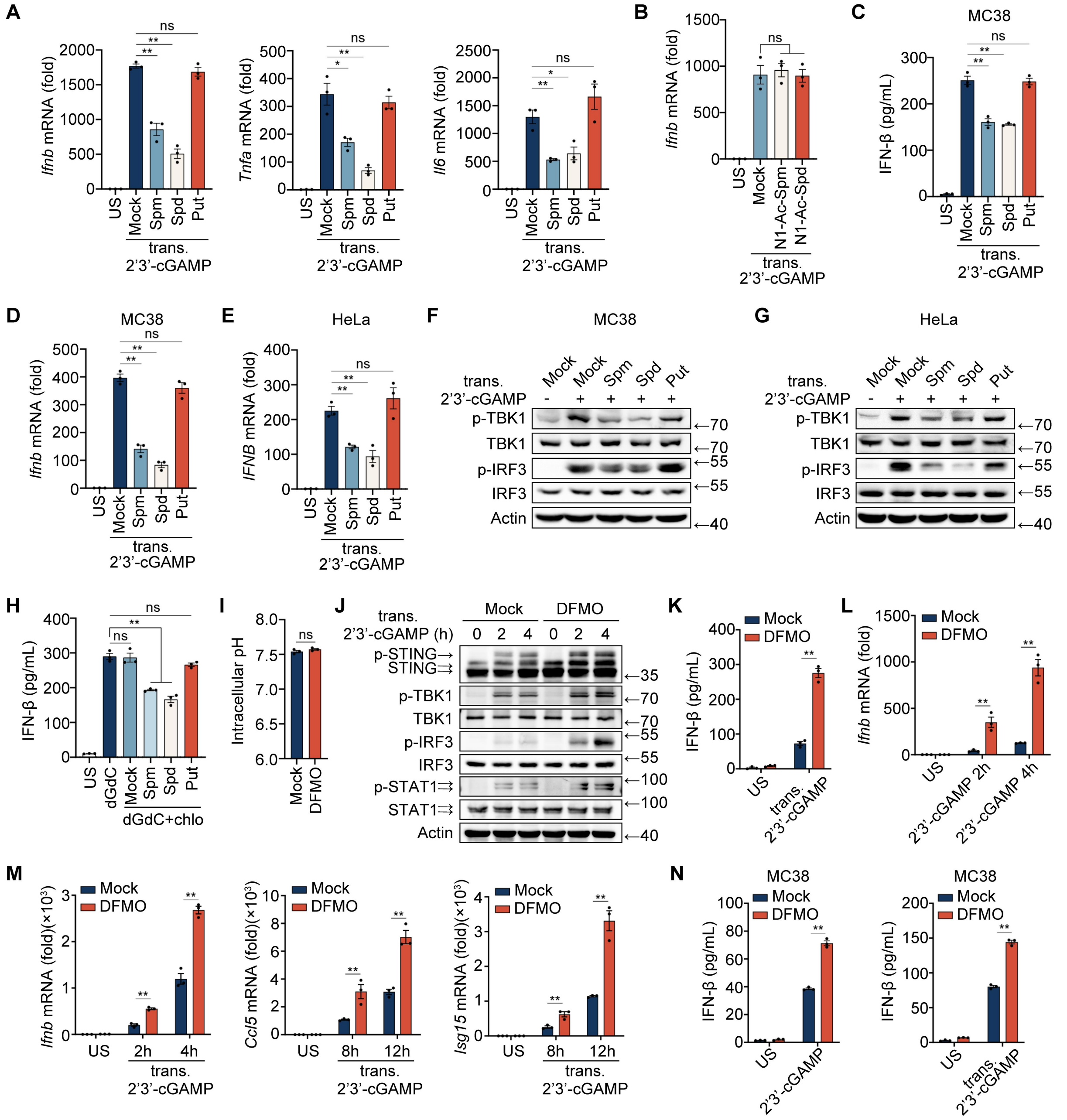

Supplementary Figure 2. Spermine and spermidine inhibit intracellular 2’3’-cGAMP-induced STING activation

(**A** and **B**) QPCR analysis of cytokines expression in mouse PMs pretreated with polyamines (**A**) or N1-acetylpolyamines (**B**), followed by transfection with 2’3’-cGAMP for 4 h.

(**C–E**) ELISA (**C**) and qPCR (**D** and **E**) analysis of IFN-β expression in tumor cells pretreated with polyamines and transfected with 2’3’-cGAMP.

(**F** and **G**) Immuno blot analysis of indicated antibodies in MC38 (**F**) and Hela cells (**G**) pretreated with polyamines for and transfected with 2’3’-cGAMP.

(**H**) Poly(dG:dC) was incubated with 100 μM chloroquine, followed by buffer exchange. ELISA analysis of IFN-β secretion in PMs pretreated with polyamines, then transfected with chloroquine-treated poly(dG:dC).

(**I**) PMs were treated with 500 μM DFMO for 24 h followed by measurement of cytosolic pH values.

(**J**) Immuno blot analysis of indicated antibodies in PMs pretreated with DFMO and transfected with 2’3’-cGAMP.

(**K**–**N**) ELISA (**K** and **N**) or qPCR (**L** and **M**) analysis of indicated mRNA levels in mouse PMs (**K**–**M**) or MC38 (**N**) pretreated with DFMO, then stimulated or transfected with 2’3’-cGAMP for the indicated hours.

Statistical significance was determined using unpaired two-sided t test and adjustments were made for multiple comparisons in **A**–**E, H**, **I** and **K**–**N**. The data are shown as the mean ± SEM. *P <0.05, **P <0.01, and ns, not significant. Similar results were obtained from three independent experiments.

Supplemental Figure 3

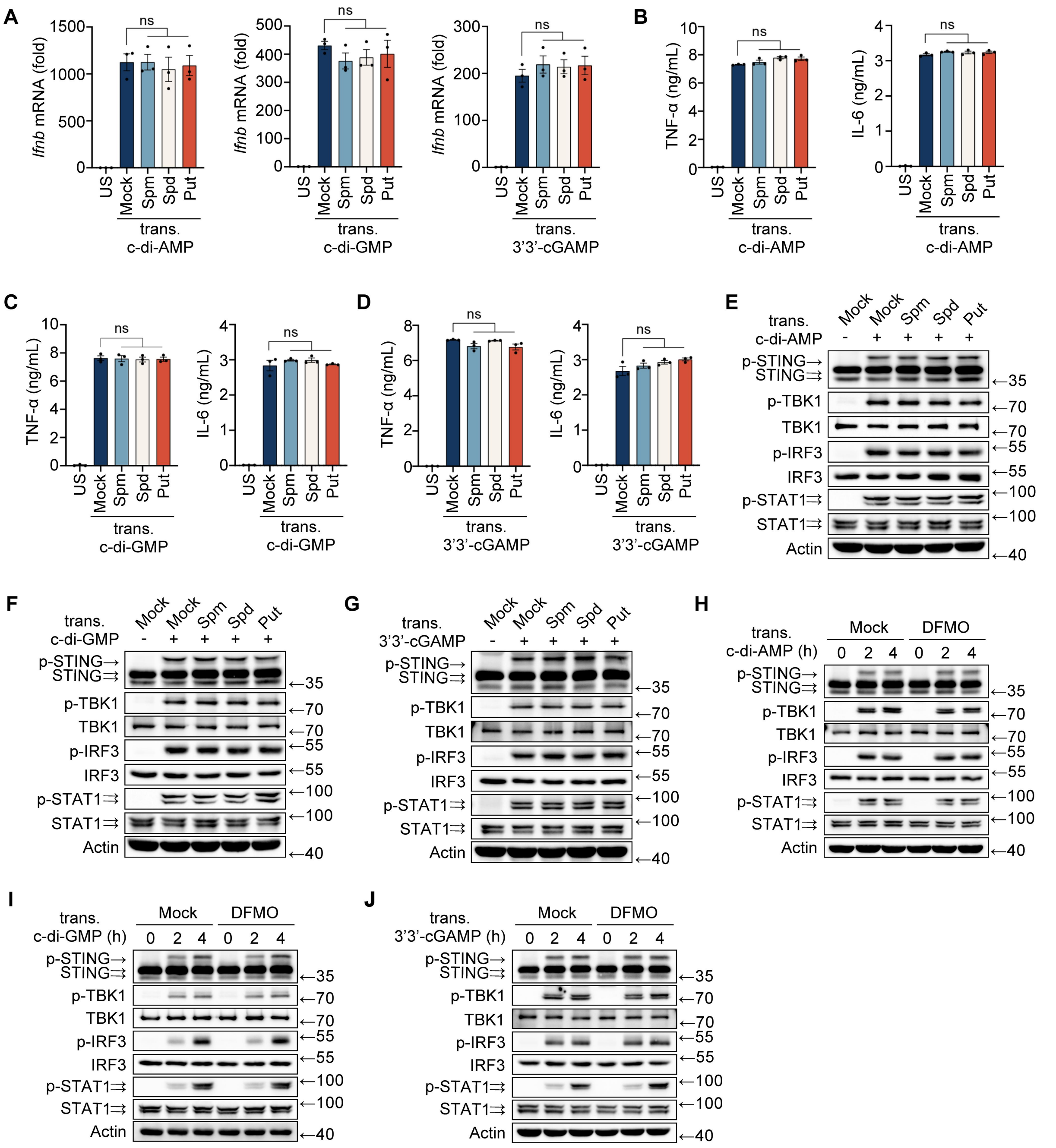

Supplementary Figure 3. Spermine and spermidine selectively inhibit intracellular 2’3’-cGAMP-induced STING activation

(**A**–**D**) QPCR (**A**) and ELISA (**B**–**D**) analysis of cytokines expression in mouse PMs pretreated with polyamines, followed by transfection with c-di-AMP, c-di-GMP, or 3’3’-cGAMP.

(**E**–**J**) Immuno blot analysis of indicated antibodies in mouse PMs pretreated with polyamines (**E**–**G**)

or DFMO (**H**–**J**) and followed by transfection with c-di-AMP, c-di-GMP, or 3'3'-cGAMP.

Statistical significance was determined using unpaired two-sided t test and adjustments were made for multiple comparisons in **A**–**D**. The data are shown as the mean ± SEM. *P <0.05, **P <0.01, and ns, not significant. Similar results were obtained from three independent experiments.

Supplemental Figure 4

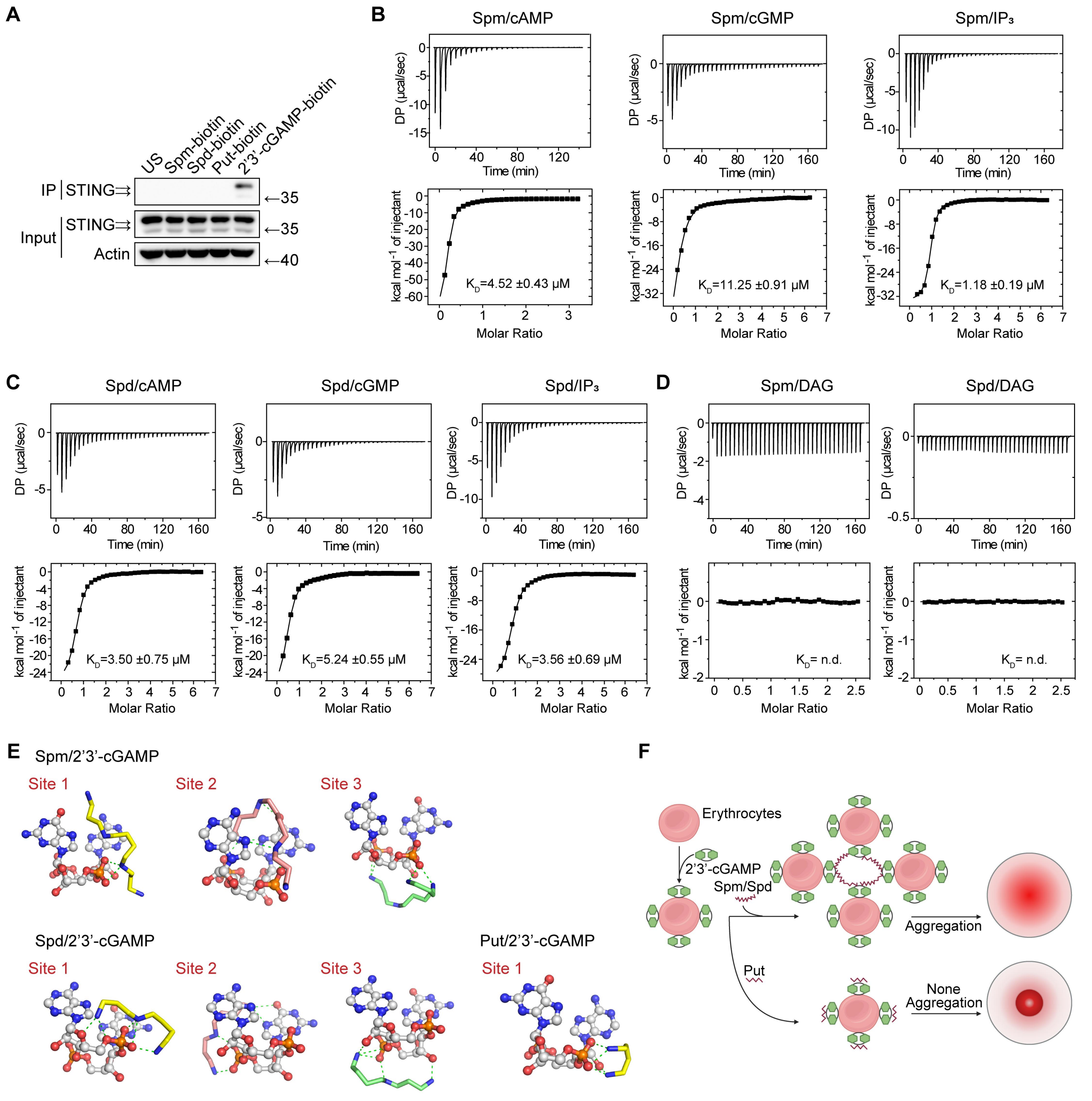

Supplementary Figure 4. Spermine and spermidine directly bind to 2'3'-cGAMP and promote its aggregation

(**A**) Immuno blot analysis of lysates from mouse PMs were pretreated with polyamines-biotin or transfected with 2'3'-cGAMP-biotin for 4 h, followed by immunoprecipitation with streptavidin.

(**B–D**) The original titration traces (top) and integrated data (bottom) of ITC experiments, where spermine (**B** and **D**) or spermidine (**C** and **D**) were titrated into a solution of cAMP, cGMP, or IP_3 (**B**

and **C**) and 1-Stearoyl-2-arachidonoyl-sn-glycerol (a diacylglycerol, DAG) (**D**).

(**E**) Molecular docking of polyamine and 2'3'-cGAMP binding conformations.

(**F**) In vitro indirect hemagglutination assay method. Figure created with Biorender.com.

Similar results were obtained from three independent experiments.

Supplemental Figure 5

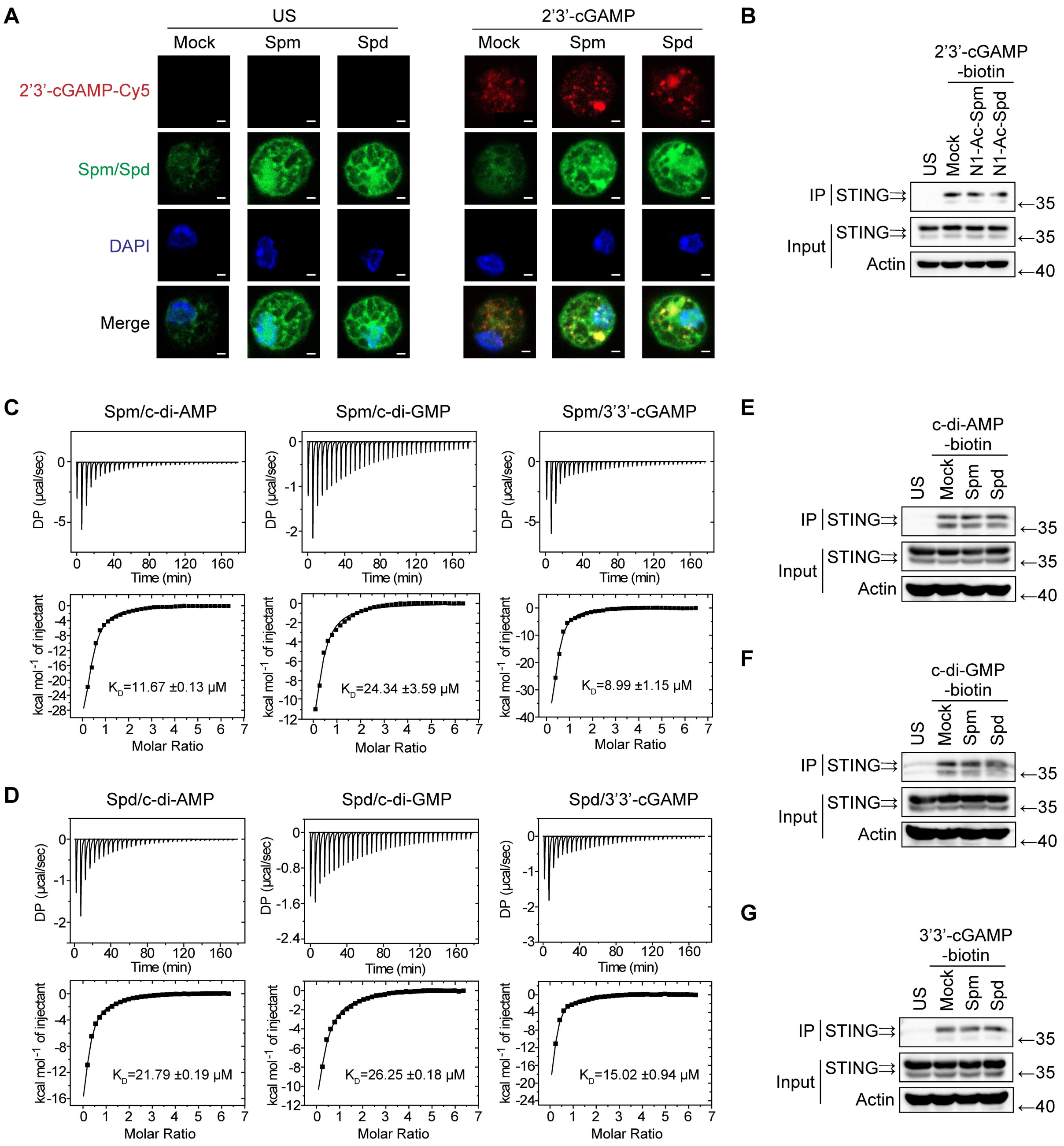

Supplementary Figure 5. Spermine and spermidine selectively suppress 2'3'-cGAMP binding to STING

(**A**)Wild-type PMs were treated with 10 μM spermine or spermidine for 1 h, then transfected with 2'3'-cGAMP-Cy5 for 1 h, before staining for spermine or spermidine. Scale bars, 2 μm.

(**B**) Immuno blot analysis of lysates from mouse PMs were pretreated with N1-Ac-Spm or N1-Ac-Spd for 1 h, then incubated with 2'3'-cGAMP-biotin for 4 h, followed by immunoprecipitation with streptavidin.

(**C** and **D**) The original titration traces (top) and integrated data (bottom) of ITC experiments, where spermine (**C**) or spermidine (**D**) was titrated into a solution of c-di-AMP, c-di-GMP, or 3'3'-cGAMP.

(**E–G**) Immuno blot analysis of lysates from mouse PMs were pretreated with spermine or spermidine, then incubated with CDN-biotin for 4 h, followed by immunoprecipitation with streptavidin.

Similar results were obtained from three independent experiments.

Supplemental Figure 6

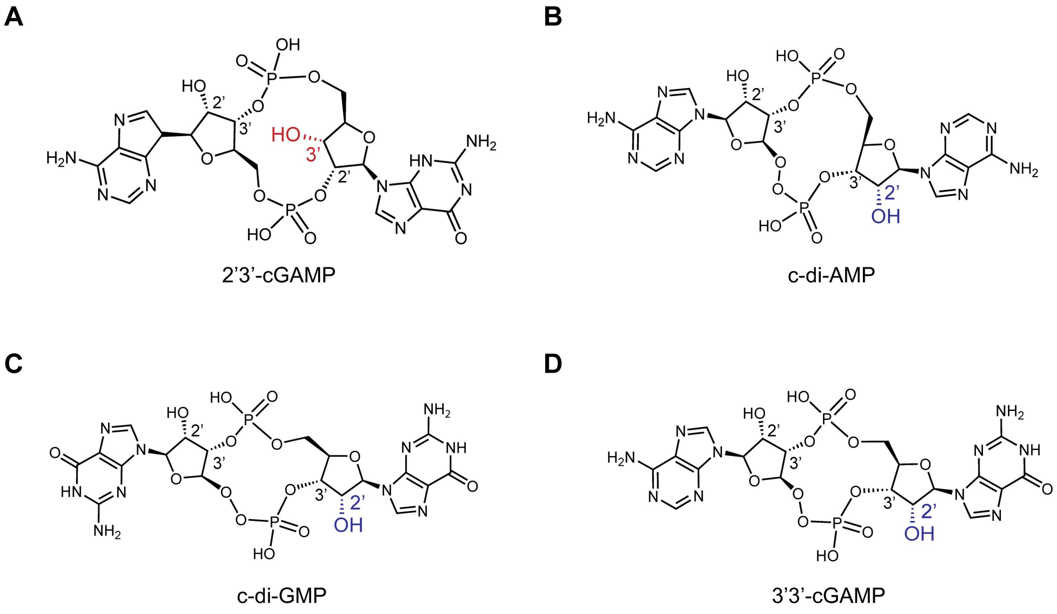

Supplementary Figure 6. The unique phosphodiester linkage architecture of 2’3’-cGAMP

Structural configurations of cyclic dinucleotides (CDNs) with different combinations of phosphodiester bonds and free hydroxyl.

Supplemental Figure 7

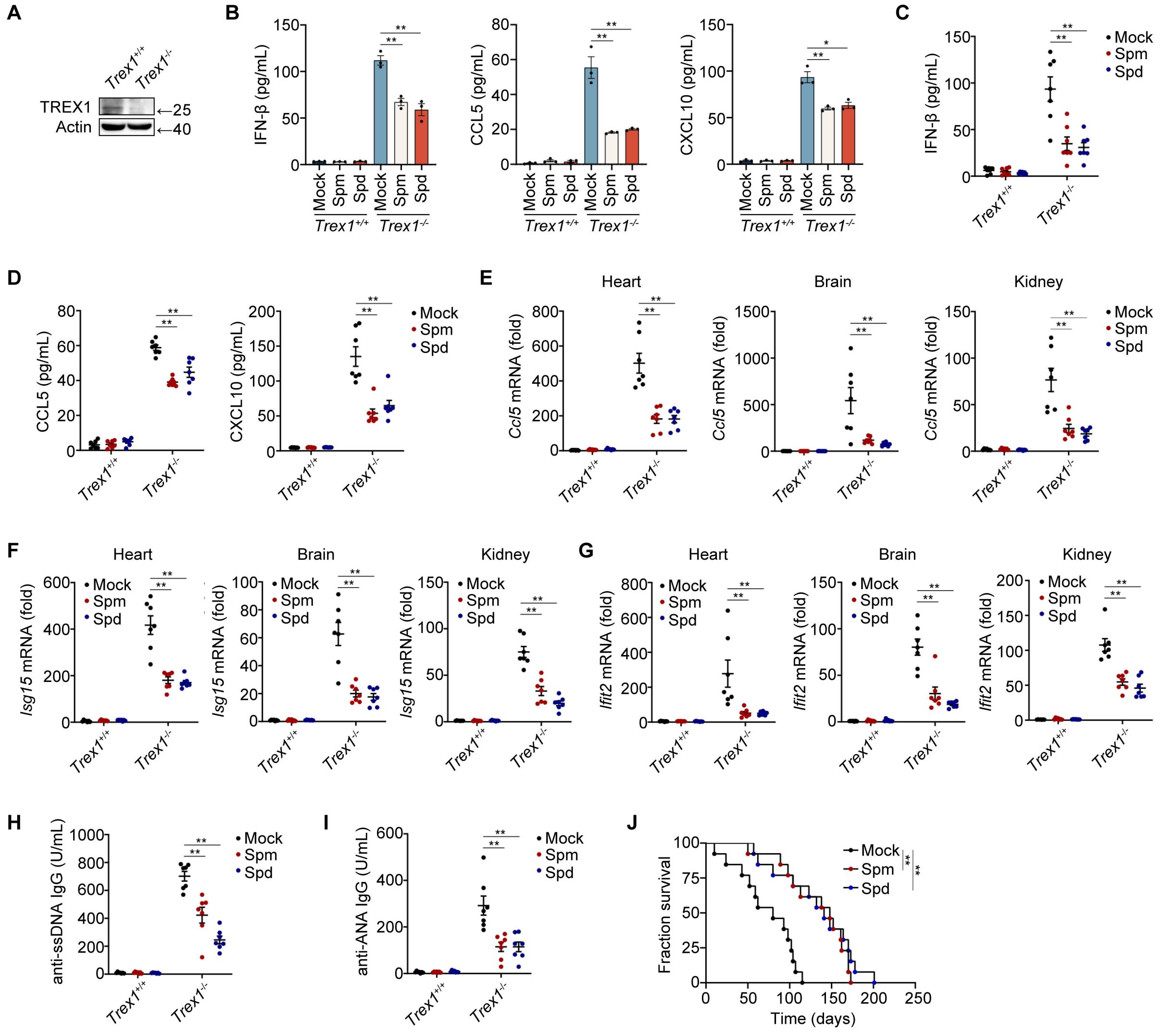

Supplementary Figure 7. Spermine and spermidine alleviate STING-dependent autoimmune disorders

(**A**) Immuno blot analysis of TREX1 in *Trex1*$^{+/+}$ or *Trex1*$^{-/-}$ mouse PMs.

(**B**) ELISA analysis of IFN-β, CCL5, and CXCL10 production in mouse PMs from *Trex1*$^{+/+}$ or *Trex1*$^{-/-}$ mice pretreated with 10 μM polyamines for 24 h.

(**C–J**) *Trex1*$^{+/+}$ or *Trex1*$^{-/-}$ mice were administered 3 mM spermine and spermidine through drinking water during the experiments. ELISA analysis of serum IFN-β (**C**), CCL5, CXCL10 (**D**), qPCR analysis of *Ccl5* (**E**), *Isg15* (**F**), and *Ifit2* (**G**) expression of the heart, brain, and kidney, ELISA analysis of serum anti-ssDNA IgG (**H**), and anti-ANA IgG (**I**) after administered for 4 weeks (n = 7

per condition). The Kaplan–Meier method was used to evaluate survival curves (**J**; n = 13 per condition).

Statistical significance was determined using unpaired two-sided t test and adjustments were made for multiple comparisons in **B**–**I,** and the log-rank Mantel-Cox test in **J**. The data are shown as the mean ± SEM. *P <0.05 and **P <0.01. Similar results were obtained from three independent experiments.

Supplemental Figure 8

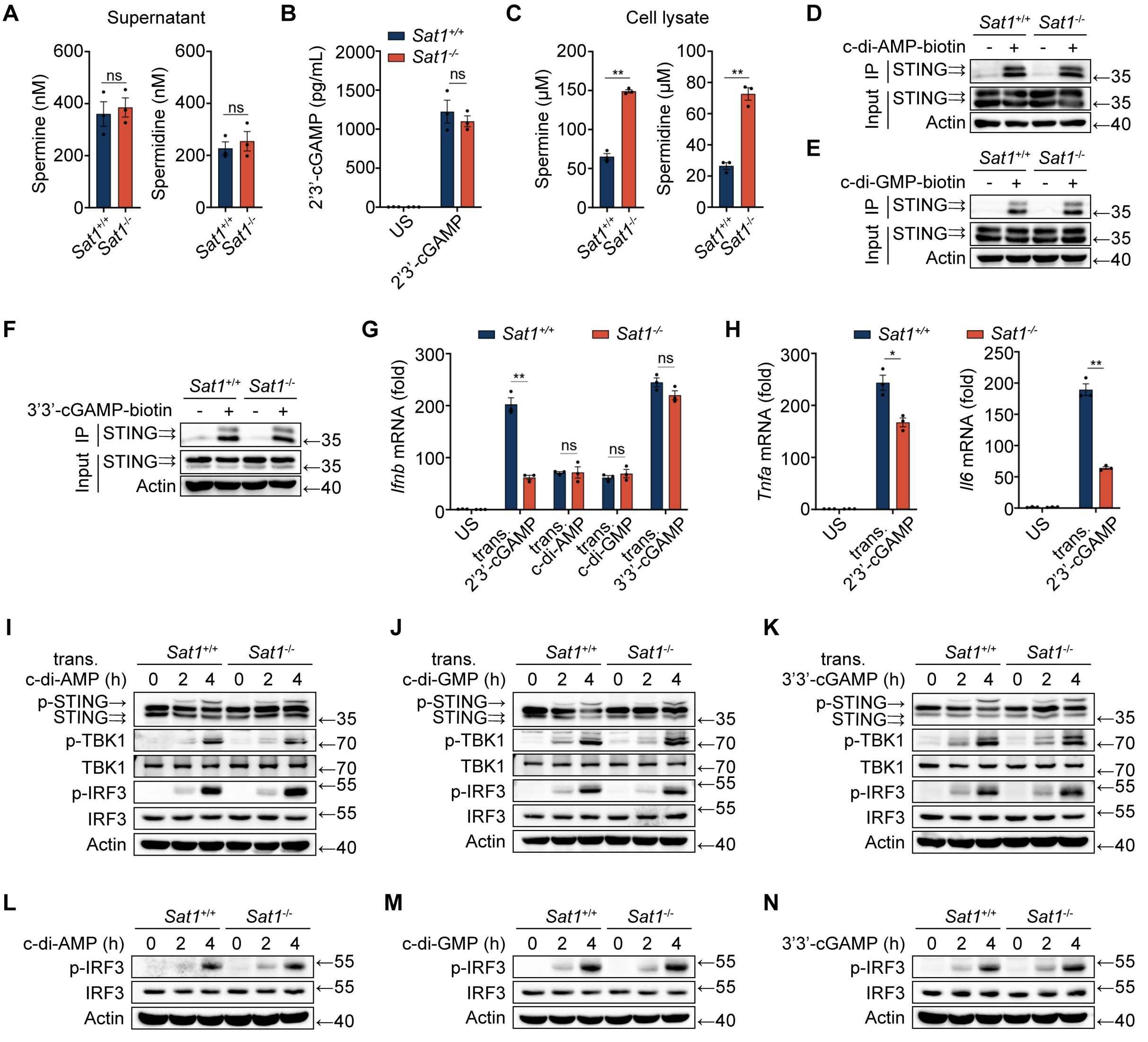

Supplementary Figure 8. SAT1 enhances 2’3’-cGAMP-induced STING activation

(**A**) MS quantification of spermine and spermidine in *Sat1*$^{+/+}$ or *Sat1*$^{-/-}$ mouse PMs supernatant.

(**B**) ELISA analysis of 2’3’-cGAMP entrance in *Sat1*$^{+/+}$ or *Sat1*$^{-/-}$ mouse PMs, followed by stimulation with 2’3’-cGAMP for 4 h.

(**C**) MS quantification of spermine and spermidine in *Sat1*$^{+/+}$ or *Sat1*$^{-/-}$ mouse PMs cell lysate.

(**D–F**) Immuno blot analysis of lysates from *Sat1*$^{+/+}$ or *Sat1*$^{-/-}$ mouse PMs incubated with c-di-AMP-biotin (**D**), c-di-GMP-biotin (**E**), or 3’3’-cGAMP-biotin (**F**) transfection for 4 h, followed by immunoprecipitation with streptavidin.

(**G** and **H**) QPCR analysis of cytokines expression in PMs from *Sat1*$^{+/+}$ or *Sat1*$^{-/-}$ mice, followed by CDNs transfection for 4 h.

(**I**–**N**) Immuno blot analysis of indicated proteins in *Sat1*$^{+/+}$ or *Sat1*$^{-/-}$ mouse PMs transfected (**I**–**K**) or stimulated (**L**–**N**) by c-di-AMP (**I** and **L**), c-di-GMP (**J** and **M**), or 3'3'-cGAMP (**K** and **N**).

Statistical significance was determined using unpaired two-sided t test and adjustments were made for multiple comparisons in **A**–**C**, **G** and **H**. The data are expressed as the mean ± SEM. *P <0.05, **P <0.01, and ns, not significant. Similar results were obtained from three independent experiments.

Supplemental Figure 9

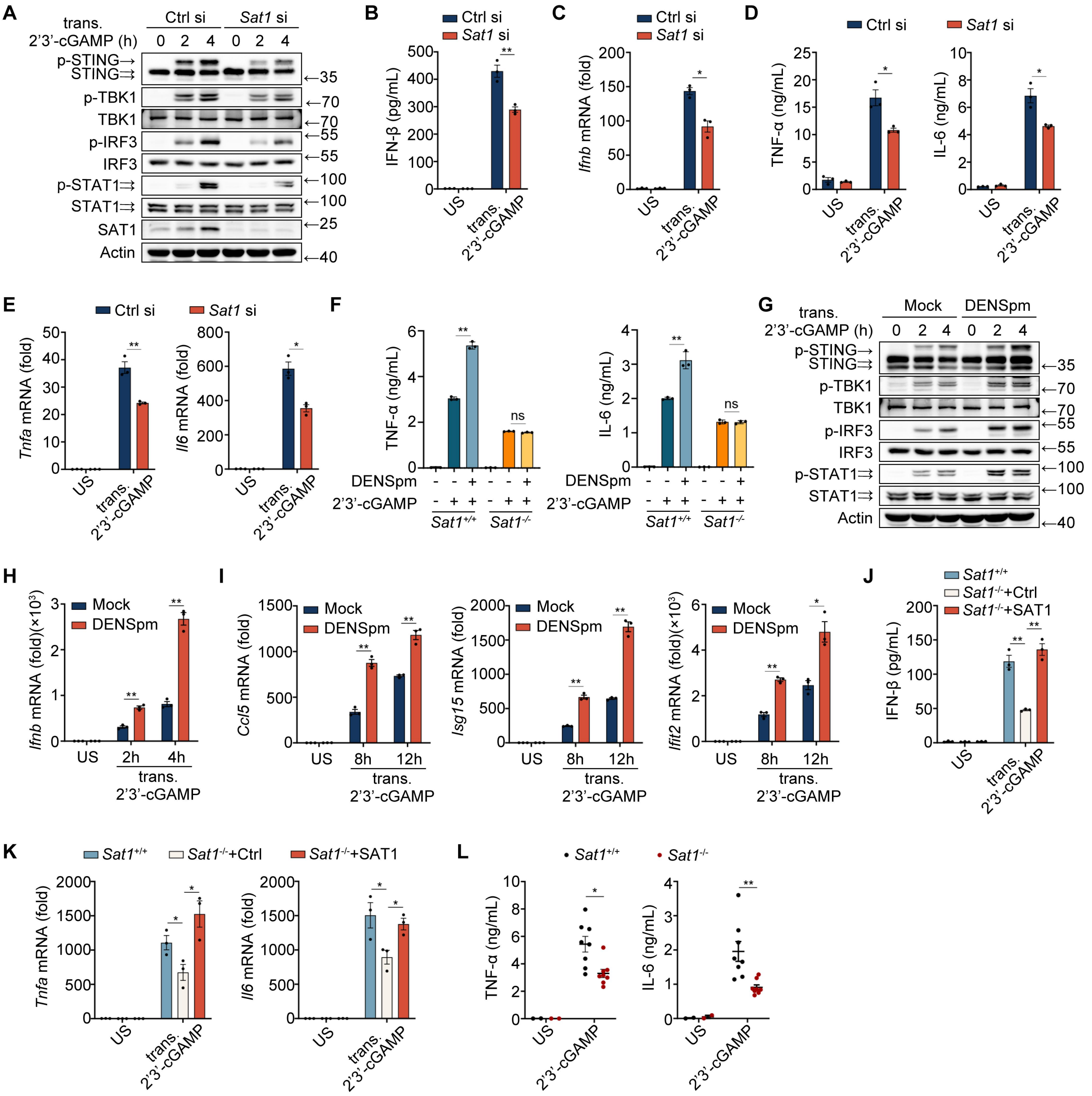

Supplementary Figure 9. SAT1 enhances 2'3'-cGAMP-induced STING activation

(**A**–**E**) Immuno blot analysis of indicated proteins (**A**), ELISA analysis of IFN-β, TNF-α, and IL-6 secretion (**B** and **D**), or qPCR analysis of *Ifnb*, *Tnfa,* and *Il6* expression (**C** and **E**) in PMs transfected with Ctrl si or *Sat1* si, followed by 2'3'-cGAMP transfection.

(**F**) ELISA analysis of TNF-α and IL-6 secretion in *Sat1*$^{+/+}$ or *Sat1*$^{-/-}$ mouse PMs, pretreated with 10 μM DENSpm for 24 h, followed by 2'3'-cGAMP stimulation for 4 h.

(**G**–**I**) Immuno blot analysis of indicated proteins (**G**) or qPCR analysis of *Ifnb* (**H**), *Ccl5*, *Isg15,* and

Ifit2 (**I**) expression in mouse PMs pretreated with 10 μM DENSpm for 24 h, followed by 2'3'-cGAMP transfection.

(**J** and **K**) ELISA analysis of IFN-β secretion (**J**) or qPCR analysis of *Tnfa* and *Il6* expression (**K**) from *Sat1*$^{+/+}$ MEFs transfected with an empty vector (*Sat1*$^{+/+}$) and *Sat1*$^{-/-}$ MEFs transfected with an empty vector (*Sat1*$^{-/-}$ +Ctrl) or SAT1 plasmid (*Sat1*$^{-/-}$ +SAT1), followed by 2'3'-cGAMP transfection for 4 h.

(**L**) *Sat1*$^{+/+}$ or *Sat1*$^{-/-}$ mice were i.p. injection of 2'3'-cGAMP (50μg per mouse). The serum cytokines were analyzed using ELISA after 2'3'-cGAMP injection for 2 h (US, n = 2; 2'3'-cGAMP, n = 8 per condition).

Statistical significance was determined using unpaired two-sided t test and adjustments were made for multiple comparisons in **B**–**F**, and **H**–**L**. The data are expressed as the mean ± SEM. *P <0.05, **P <0.01, and ns, not significant. Similar results were obtained from three independent experiments.

Supplemental Figure 10

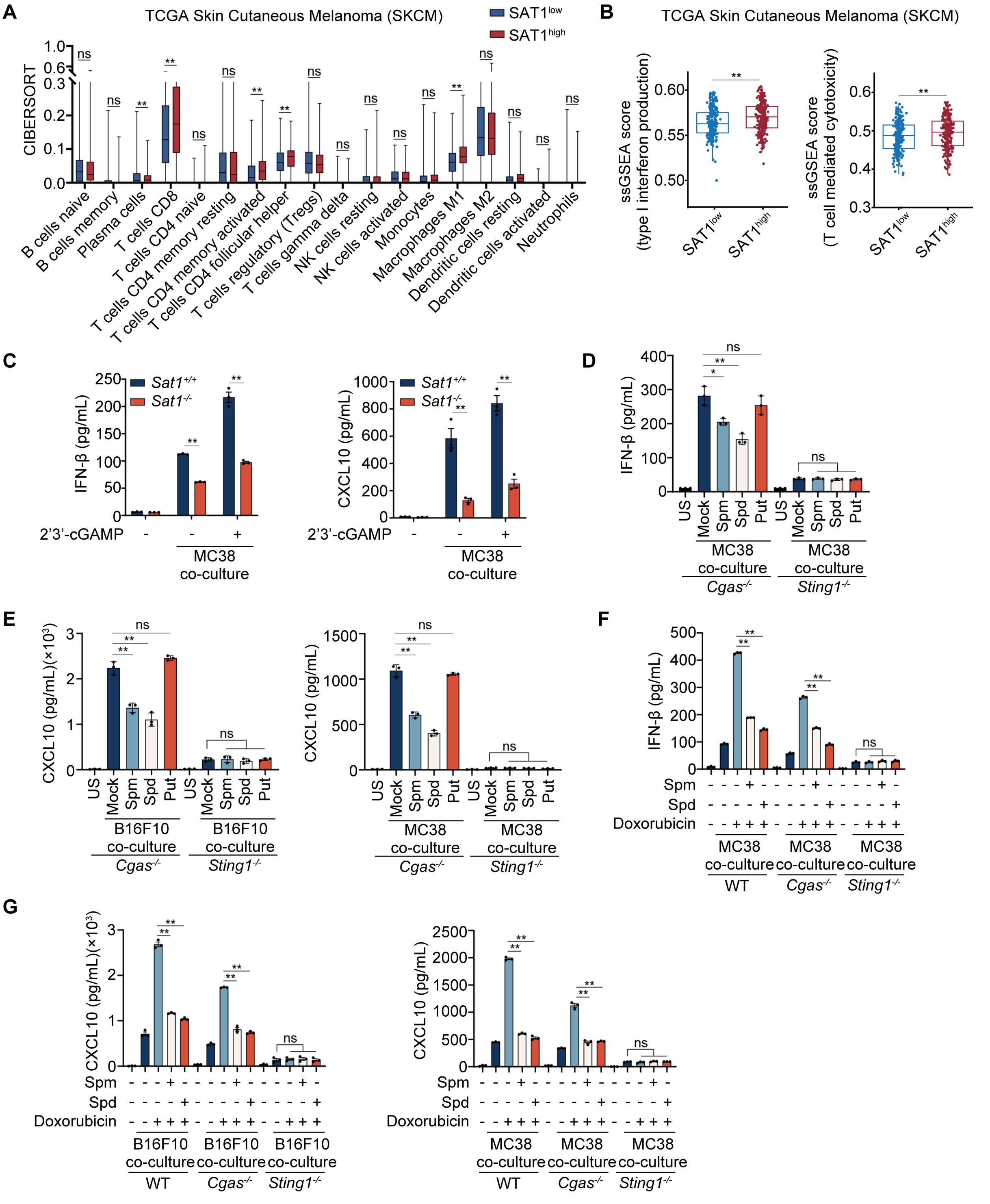

Supplementary Figure 10. *Sat1* deficiency attenuates STING-driven antitumor immunity

(**A**) Different infiltration levels of 18 immune cells in SAT1 high and low tumors analyzed using the CIBERSORT algorithm in the TCGA Skin Cutaneous Melanoma (SKCM) database ($SAT1^{low}$, n = 236 and $SAT1^{high}$, n = 237 per condition).

(**B**)The ssGSEA score of type I interferon production (left) or T cell-mediated cytotoxicity (right) in SAT1^{low} and SAT1^{high} clusters, divided by the value of GOBP_TYPE_I_INTERFERON_PRODUCTION (left) or GOBP_T_CELL_MEDIATED_ CYTOTOXICITY (right) gene set, in the TCGA Skin Cutaneous Melanoma (SKCM) database (SAT1^{low}， n = 236 and SAT1^{high}, n = 237 per condition).

(**C**) ELISA analysis of IFN-β and CXCL10 secretion in *Sat1*$^{+/+}$ or *Sat1*$^{-/-}$ BMDMs coculture with MC38, pretreated with 10 μM polyamines, followed by 2’3’-cGAMP stimulation.

(**D** and **E**) ELISA analysis of IFN-β (**D**) or CXCL10 (**E**) secretion in *Cgas*$^{-/-}$ or *Sting1*$^{-/-}$ BMDMs coculture with MC38 or B16F10 pretreated with 10 μM polyamines.

(**F** and **G**) ELISA analysis of IFN-β (**F**) or CXCL10 (**G**) secretion in wild-type, *Cgas*$^{-/-}$ or *Sting1*$^{-/-}$ BMDMs coculture with MC38 or B16F10 pretreated with polyamines, then treated with Doxorubicin or not.

Statistical significance was determined using the rank-sum Wilcoxon test in **A** or unpaired two-sided t test and adjustments were made for multiple comparisons in **B**–**G**. The data are shown as the mean ± SEM. *P <0.05, **P <0.01, and ns, not significant. Similar results were obtained from three independent experiments.

Supplemental Figure 11

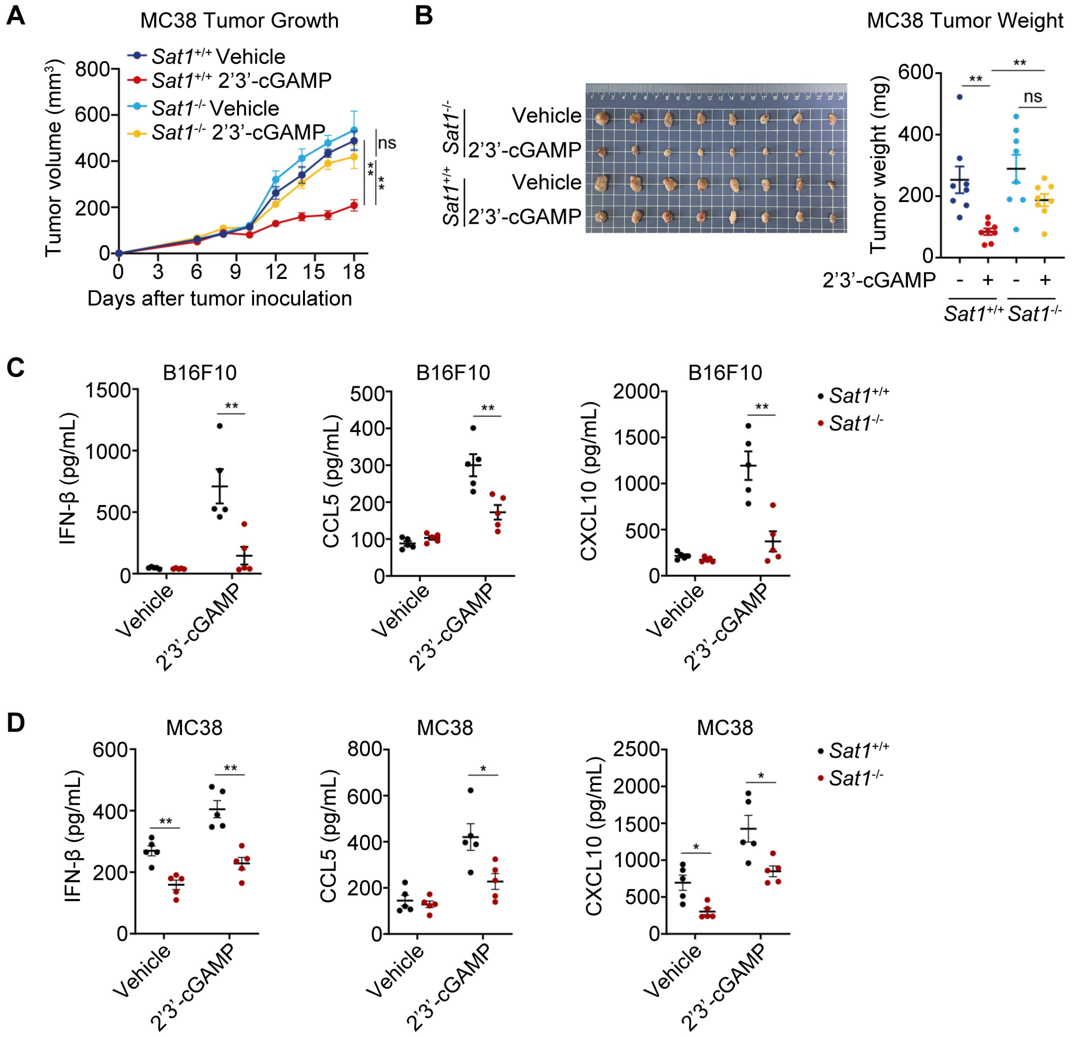

Supplementary Figure 11. ***Sat1*** **deficiency attenuates STING-driven antitumor immunity**

(**A** and **B**) Tumor volume curve (**A**) and tumor weight (**B**) of *Sat1*$^{+/+}$ or *Sat1*$^{-/-}$ mice after MC38 inoculation treated with 2'3'-cGAMP (n = 8 per condition).

(**C**) *Sat1*$^{+/+}$ or *Sat1*$^{-/-}$ mice at day 13 postinoculation with B16F10. ELISA analysis of IFN-β, CCL5, and CXCL10 concentration in tumor (n = 5 per condition).

(**D**) *Sat1*$^{+/+}$ or *Sat1*$^{-/-}$ mice at day 13 postinoculation with MC38 cancer cells. ELISA analysis of IFN-β, CCL5, and CXCL10 concentration in tumor (n = 5 per condition).

Statistical significance was determined using two-way ANOVA in **A** or unpaired two-sided t test and adjustments were made for multiple comparisons in **B**–**D**. The data are shown as the mean ± SEM. *P

<0.05, **P <0.01, and ns, not significant. Similar results were obtained from three independent experiments.

Supplemental Figure 12

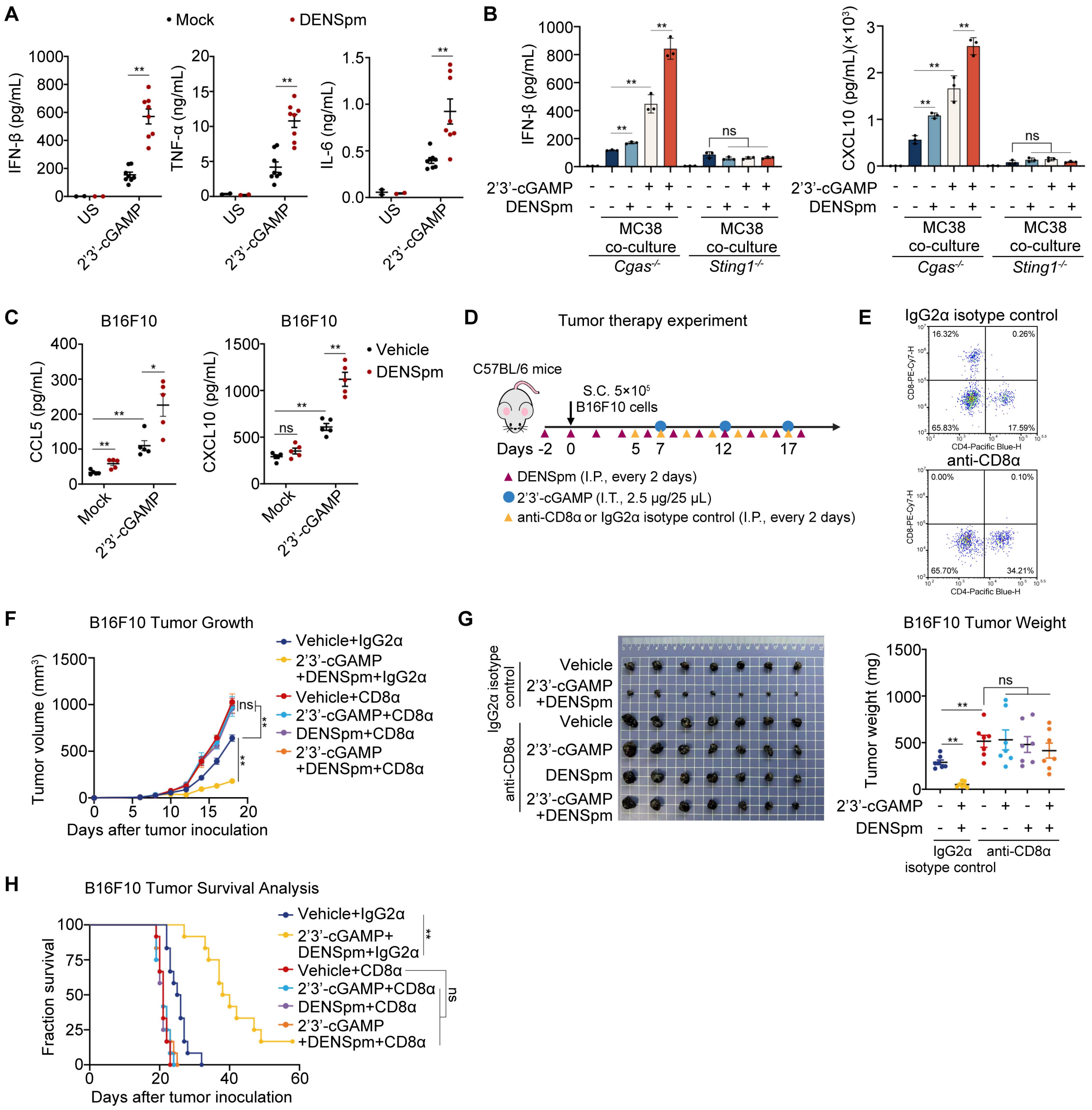

Supplementary Figure 12. DENSpm potentiates antitumor immunity via polyamine catabolic reprogramming

(**A**) Wild-type mice were treated with DENSpm (120 nmol per mouse) via i.p. injection for 1 day. Mice were then infected via i.p. injection of 2'3'-cGAMP (50μg per mouse). The serum cytokines were analyzed using ELISA after 2'3'-cGAMP injection for 2 h (US, n = 2; 2'3'-cGAMP, n = 8 per condition).

(**B**) ELISA analysis of IFN-β and CXCL10 secretion in *Cgas*$^{-/-}$ or *Sting1*$^{-/-}$ BMDMs coculture with

MC38, pretreated with 10 μM DENSpm, followed by 2’3’-cGAMP stimulation.

(**C**) C57BL/6 mice at day 13 after B16F10 tumor inoculation treated with DENSpm/2’3’-cGAMP combination. ELISA analysis of CCL5 and CXCL10 secretion in tumor (n = 5 per condition).

(**D**) Experimental scheme for C57BL/6 mice bearing B16F10 subcutaneous tumors with $CD8^+$ T cell depletion.

(**E**) Anti-CD8α depletes $CD8^+$ T cells in tumors, with IgG2a as the isotype control.

(**F–H**) Tumor volume curve (**F**, n = 7 per condition), tumor weight (**G**, n = 7 per condition) or survival (**H**, n = 12 per condition) of C57BL/6 mice after B16F10 tumor inoculation treated with DENSpm/2’3’-cGAMP combination under $CD8^+$ T cell depletion.

Statistical significance was determined using unpaired two-sided t test and adjustments were made for multiple comparisons in **A–C**, and **G** or two-way ANOVA in **F** and **H**. The data are expressed as the mean ± SEM. *P <0.05, **P <0.01, and ns, not significant. Similar results were obtained from three independent experiments.

Supplemental Figure 13

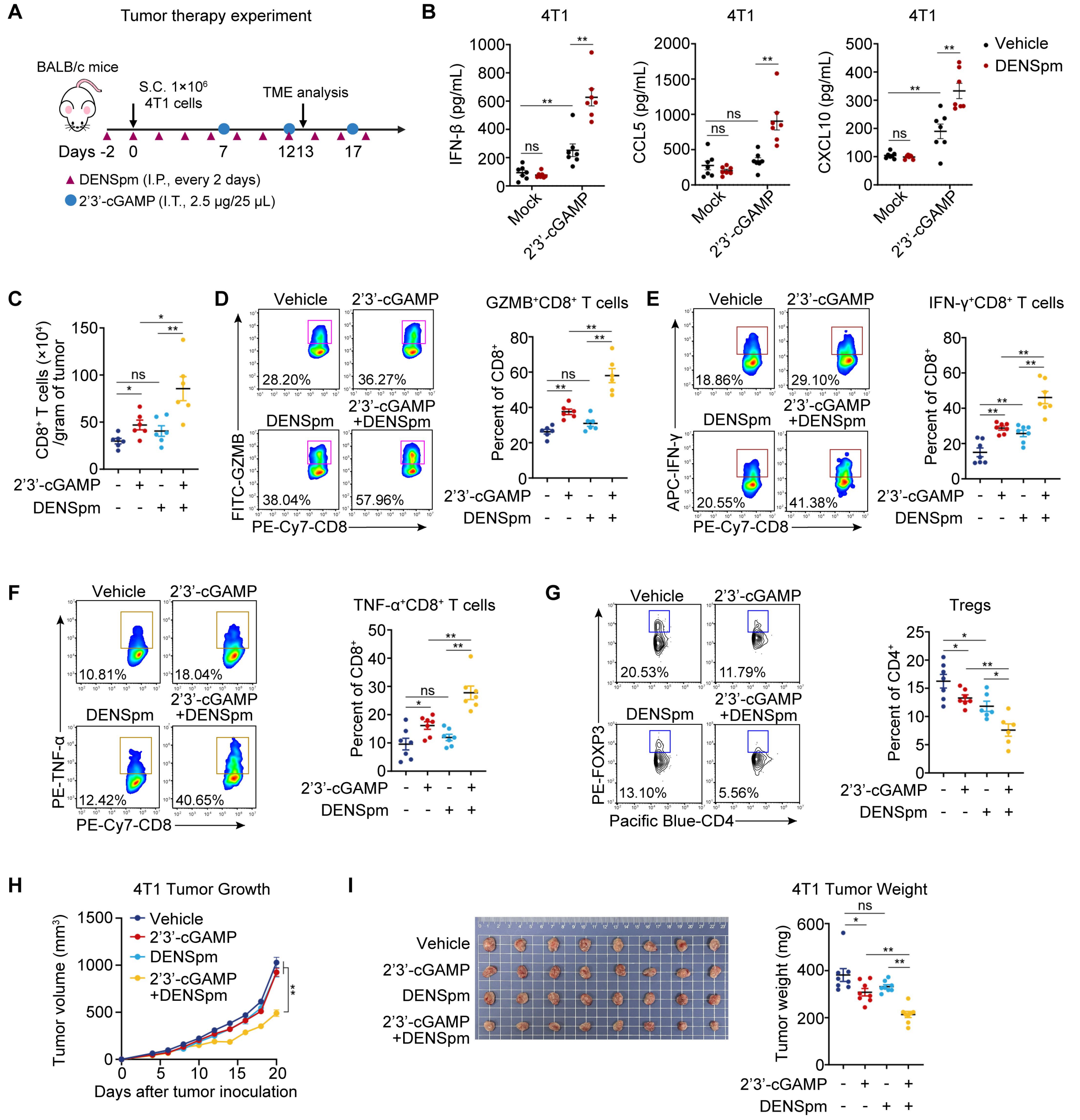

Supplementary Figure 13. DENSpm potentiates antitumor immunity via polyamine catabolic reprogramming

(**A**) Experimental scheme for BALB/c mice bearing 4T1 subcutaneous tumors.

(**B**–**G**) BALB/c mice at day 13 postinoculation with 4T1 cancer cells. ELISA analysis of IFN-β, CCL5, and CXCL10 secretion in tumor (**B**; n = 7 per condition). Flow cytometry analysis showing the percentage of total intratumoral CD8+ T cells (**C**; n = 6 per condition), intratumoral CD8+ T cells

expressing GZMB (**D**; n = 6 per condition), IFN-γ (**E**; n = 7 per condition), and TNF-α (**F**; n = 7 per condition) or $CD4^+FOXP3^+$ Treg cells (**G**; Vehicle, n = 7; DENSpm, n=6 per condition) in 4T1 tumor. (**H** and **I**) Tumor volume curve (**H**; n = 10 per condition) and tumor weight (**I**; n = 8 per condition) of BALB/c mice after 4T1 tumor inoculation treated with DENSpm/2'3'-cGAMP combination.

Statistical significance was determined using unpaired two-sided t test and adjustments were made for multiple comparisons in **B–G**, and **I** or two-way ANOVA in **H**. The data are expressed as the mean ± SEM. *P <0.05, **P <0.01, and ns, not significant. Similar results were obtained from three independent experiments.

Supplemental Figure 14

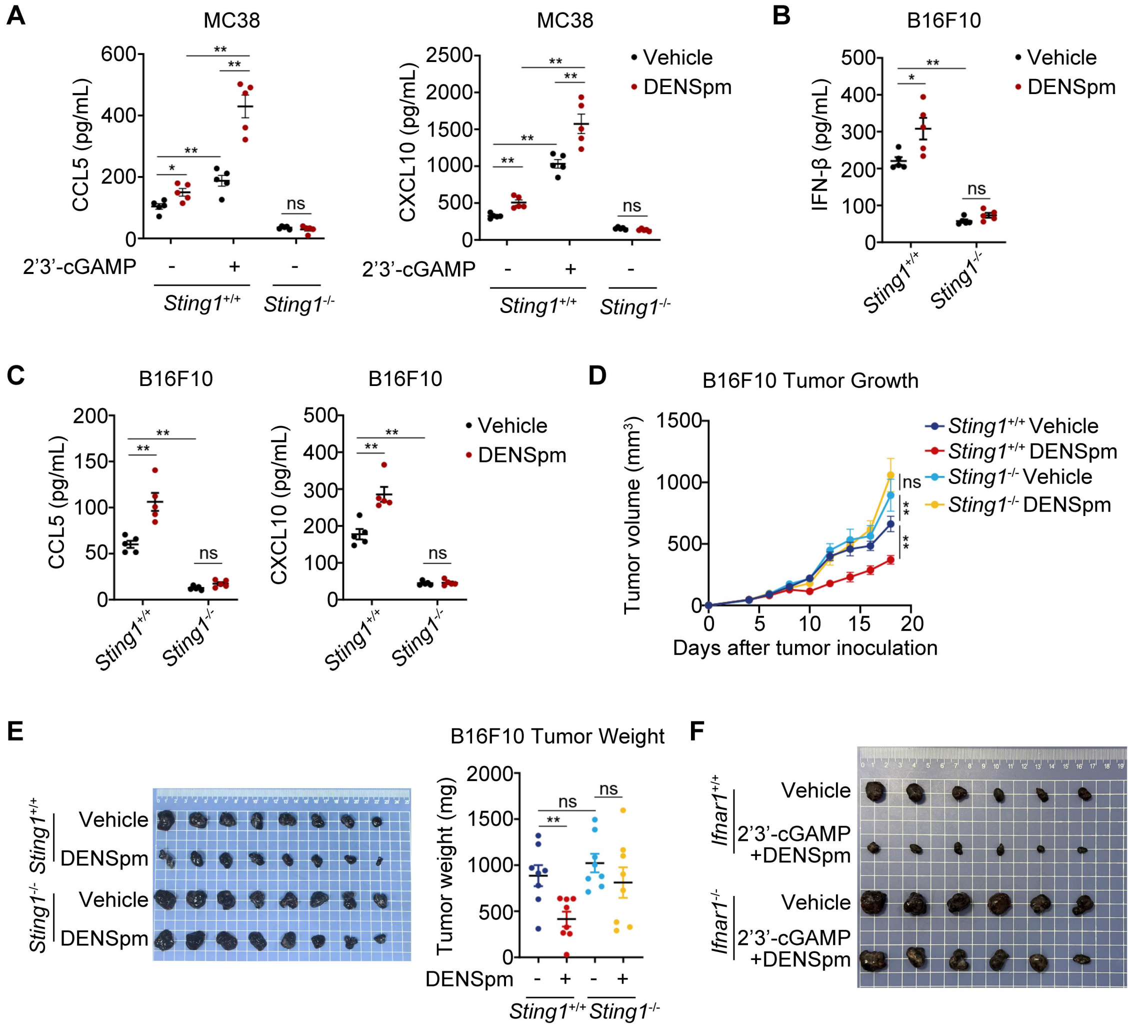

Supplementary Figure 14. DENSpm potentiates antitumor immunity dependent on STING signaling via polyamine catabolic reprogramming

(**A–E**) *Sting1*$^{+/+}$ or *Sting1*$^{-/-}$ mice were treated with DENSpm (120 nmol per mouse) via i.p. injection and supplemented every 2 days. On the day of the second DENSpm injection, the mice were inoculated subcutaneously with MC38 (**A**) or B16F10 (**B–E**) tumor cells. At day 13 after MC38 tumor inoculation, *Sting1*$^{+/+}$ mice treated with DENSpm/2'3'-cGAMP combination or *Sting1*$^{-/-}$ mice treated with DENSpm. ELISA analysis in tumor (**A**, n = 5 per condition). At day 13 after B16F10 tumor inoculation, *Sting1*$^{+/+}$ or *Sting1*$^{-/-}$ mice treated with DENSpm. ELISA analysis in tumor (**B** and **C**, n = 5 per condition). Tumor volume curve (**D**) and tumor weight (**E**) after tumor inoculation treated with

DENSpm (n = 8 per condition).

(**F**) *Ifnar1*$^{+/+}$ or *Ifnar*$^{-/-}$ mice were treated with DENSpm (120 nmol per mouse) via i.p. injection and supplemented every 2 days. On the day of the second DENSpm injection, the mice were inoculated subcutaneously with B16F10 tumor cells. Tumor weight of *Ifnar1*$^{+/+}$ or *Ifnar*$^{-/-}$ mice treated with DENSpm/2’3’-cGAMP combination after B16F10 tumor inoculation (n = 6 per condition).

Statistical significance was determined using unpaired two-sided t test and adjustments were made for multiple comparisons in **A**–**C**, and **E** or two-way ANOVA in **D**. The data are shown as the mean ± SEM. *P <0.05, **P <0.01, and ns, not significant. Similar results were obtained from three independent experiments.

Supplemental Figure 15

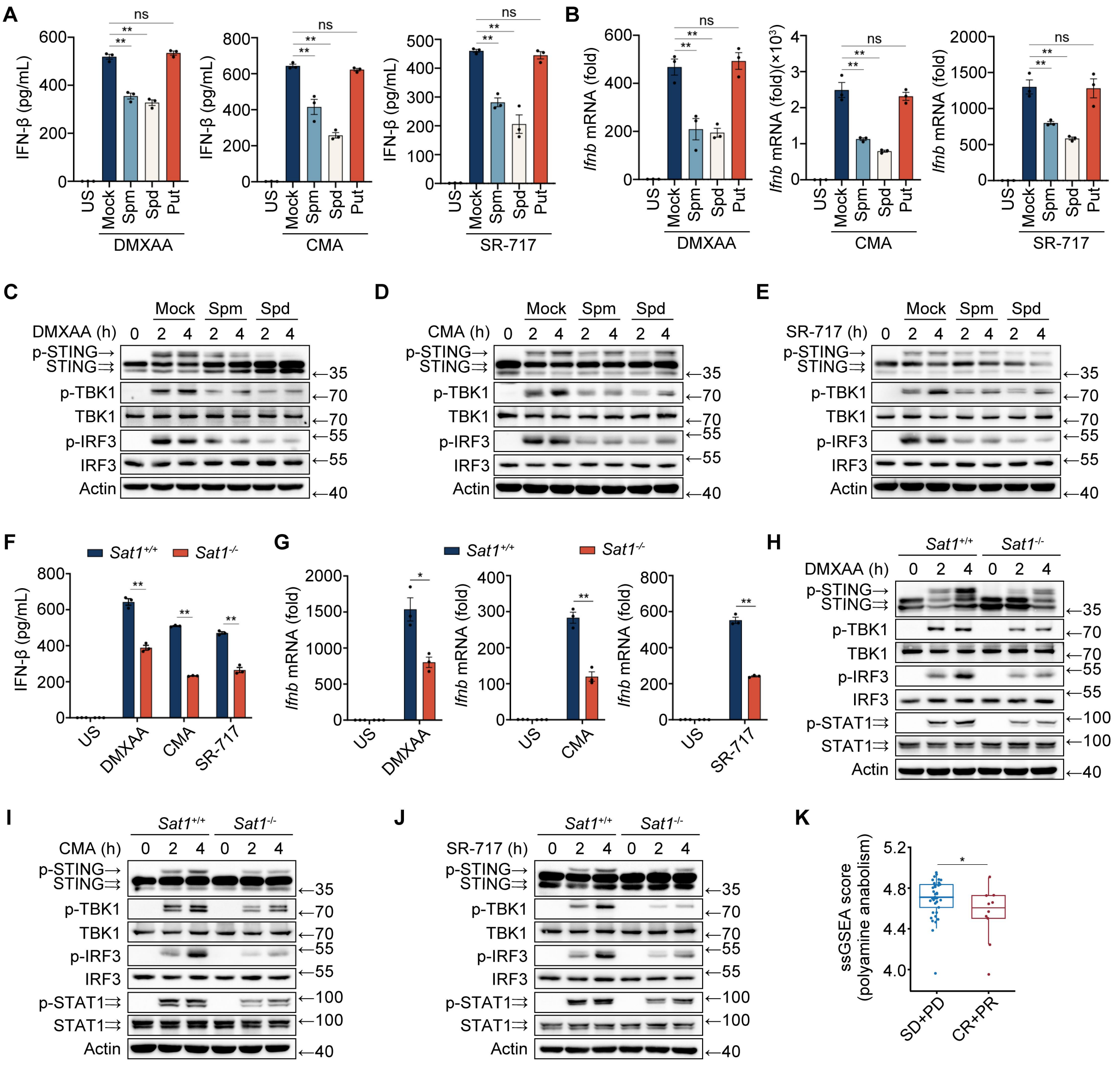

Supplementary Figure 15. DENSpm potentiates antitumor immunity dependent on STING signaling via polyamine catabolic reprogramming

(**A**–**E**) ELISA (**A**), qPCR (**B**) analysis of cytokines expression，and immuno blot analysis of indicated antibodies (**C**–**E**) in mouse PMs pretreated with polyamines, followed by treatment with CMA, DMXAA or SR-717.

(**F**–**J**) ELISA (**F**), qPCR (**G**) analysis of cytokines expression，and immuno blot analysis of indicated antibodies (**H**–**J**) in *Sat1*$^{+/+}$ or *Sat1*$^{-/-}$ mouse PMs, followed by treatment with CMA, DMXAA or

SR-717.

(**K**) The ssGSEA score of polyamine production in complete response (CR)/ partial response (PR) to nivolumab (Nivo; anti-PD-1 agent) therapy, and stable disease (SD)/ progressive disease (PD) clusters, divided by the value of "polyamine anabolism (including ODC, SRM, and SMS)" gene set, in the CA209-038 study dataset (CR/PR，n = 10 and SD/PD, n = 39 per condition).

Statistical significance was determined using unpaired two-sided t test and adjustments were made for multiple comparisons in **A**, **B**, **F**, **G** and **K**. The data are shown as the mean ± SEM. *P <0.05, **P <0.01, and ns, not significant. Similar results were obtained from three independent experiments.

Supplemental Figure 16

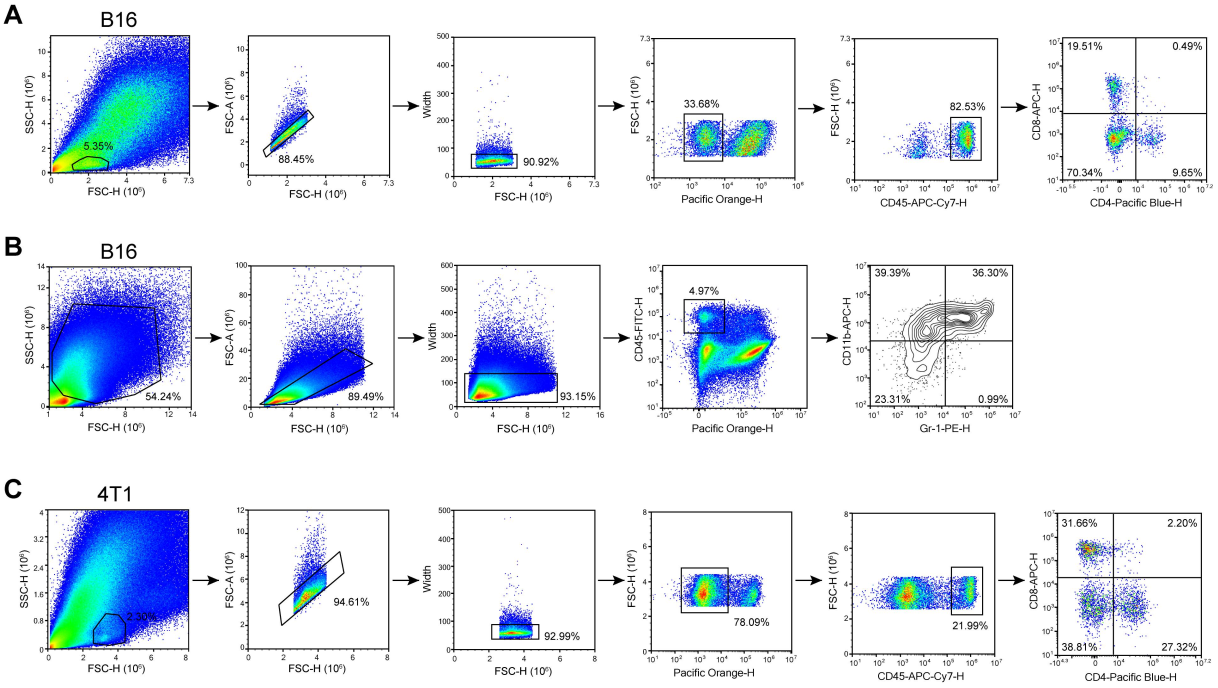

Supplementary Figure 16. Flow cytometry analyses gating strategies

(**A** and **B**) Gating strategy used to identify CD8$^+$ cells, CD4$^+$ cells (**A**), or MDSCs (**B**) for analysis in the B16F10 tumor tissue through flow cytometry.

(**C**) Gating strategy used to identify CD8$^+$ and CD4$^+$ cells for analysis in the 4T1 tumor tissue through flow cytometry. MDSC, myeloid-derived suppressor cell.

Supplementary Table 1. ***In vitro*** **extracellular 2'3'-cGAMP-induced STING activation assay**

**Metabolites**	**Fold change**	**P value**
Boldenone Undecylenate	0.985333	0.415927
Pantethine	0.820186	0.000268
D-α-Tocopherol acetate	0.759451	0.000902
α-Vitamin E	0.940971	0.015737
Acetylcholine chloride	1.052163	0.018581
D-Panthenol	1.004236	0.787188
Vitamin K1	0.501213	0.000002
Inosine	0.919451	0.010568
Dehydrocholic acid	0.816360	0.000643
L-Tryptophan	0.789520	0.000127
Glucosamine	0.866204	0.000511
i-Inositol	1.031507	0.073235
Phenylephrine hydrochloride	1.056003	0.143683
Cortisone acetate	0.774563	0.000096
Taurine	0.841750	0.000658
Dichlorisone Acetate	0.720334	0.000030
Diphenhydramine	0.858920	0.003850
Progesterone	1.084682	0.039394
Creatinine	0.967166	0.104714
L-Pyroglutamic acid	1.106020	0.000955

γ-Aminobutyric acid	1.002307	0.859308
Olaquindox	1.192617	0.000999
α-Lipoic Acid	0.940291	0.094164
Sodium erythorbate	0.877583	0.000573
Corticosterone	0.813142	0.002653
L-Ascorbic acid 2-phosphate trisodium	0.890656	0.001174
Cytidine	1.206131	0.000948
DL-Adrenaline Hydrochloride	0.997057	0.872196
D-Galactose	0.992191	0.803289
Pyridoxine	1.015443	0.425539
Ethisterone	0.716781	0.000515
6-Benzylaminopurine	0.991631	0.811262
Estrone	1.261084	0.000607
Vitamin B12	1.022913	0.710425
Estradiol	1.307637	0.000022
Retinoic acid	1.220117	0.000374
Isoprenaline hydrochloride	0.888459	0.019436
L-Glutathione reduced	1.177281	0.000465
Vitamin D2	0.386282	0.000001
Biotin	0.787256	0.000171
Taurocholic acid sodium salt hydrate	0.997682	0.880532
Riboflavin phosphate sodium	0.804460	0.000461

N-Acetyl-5-hydroxytryptamine	0.945239	0.013792
Hypoxanthine	0.871354	0.001636
Allantoin	0.950309	0.024697
Lithocholic acid	0.925312	0.004349
5α-Cholestan-3-one	0.982079	0.515858
Flavonol	0.694101	0.000326
Flavone	0.548538	0.000004
Glycocholic acid	0.819553	0.000157
Deoxycholic acid	0.790805	0.000667
Glucosamine hydrochloride	0.823714	0.000227
5α-Cholestan-3β-ol	0.707751	0.000198
Cortodoxone	0.774323	0.000699
2-Amino-5-ureidopentanoic acid	0.958825	0.033785
Cortisone	0.704856	0.000020
6-Hydroxyflavone	0.633026	0.000158
Aleuritic Acid	0.737674	0.000141
Cholic Acid	0.904566	0.004362
Spermine	0.540460	0.000005
Xanthurenic Acid	0.900204	0.002023
2-Amino-4-(2-aminophenyl)-4-oxobutanoic acid	1.000831	0.960447
Trenbolone acetate	0.600973	0.000071

Tryptamine	0.863475	0.008176
Ursodeoxycholic acid	0.795210	0.001606
D-Phenylalanine	0.945906	0.056198
Cholesterol	0.727166	0.000028
DL-Carnitine	0.876101	0.026231
Pyrithioxin	0.711506	0.000049
L-Ornithine	1.036540	0.043777
Urea	0.833563	0.000177
Niacin	0.992313	0.815718
Tramiprosate	1.028467	0.571382
Thiamine hydrochloride	1.056219	0.012845
Adrenosterone	0.981583	0.532551
Serotonin hydrochloride	1.077383	0.066936
L-5-Hydroxytryptophan	0.847679	0.000660
Pyridoxine hydrochloride	0.870703	0.003830
Batilol	0.858132	0.000864
5-hydroxytryptophan	0.712558	0.000022
Norcantharidin	1.017715	0.340650
Hyodeoxycholic acid	0.679363	0.000023
Chondroitin sulfate	0.789984	0.000060
L-Carnitine	0.949237	0.085435
Chenodeoxycholic acid	0.811943	0.000355

Adenosine	1.015514	0.400874
L-Thyroxine	1.020405	0.597457
L-Ascorbic acid	1.025297	0.423739
Riboflavin	0.900215	0.002832
DL-Panthenol	1.148282	0.000642
Ethynyl estradiol	0.882379	0.000810
17α-Hydroxyprogesterone	0.863298	0.001304
Pregnanediol	1.064190	0.111552
2'-Deoxyinosine	1.077467	0.012243
Adenosine 5'-diphosphate	1.115567	0.011532
8-Azaguanine	1.112104	0.016573
Adenosine monophosphate	0.934600	0.034028
Kinetin	1.125381	0.001205
Estriol	0.725329	0.000068
Uridine	1.189607	0.000835
NAD+	1.139813	0.000798
Isotretinoin	0.910578	0.009321
Hydrocortisone	0.803960	0.000284
N-Acetylneuraminic acid	0.857972	0.000501
Dopamine hydrochloride	0.865155	0.000483
Liothyronine	0.815963	0.000120
Melatonin	1.006506	0.823900

Vitamin K4	1.336080	0.000345
Thymopentin	0.994003	0.756380
Tauroursodeoxycholate	0.759612	0.000064
Forskolin	0.667071	0.000014
Cyclopamine	1.100808	0.001340
Bilirubin	0.937501	0.007447
Sodium Demethylcantharidate	0.942731	0.020468
Beta-Sitosterol	0.844926	0.001301
Methylcobalamin	0.867416	0.001649
Astaxanthin	0.784255	0.000199
D-(+)-Trehalose dihydrate	1.205811	0.000329
Bufalin	0.680931	0.000022
Protoporphyrin IX	3.046083	0.000000
Histamine	1.112208	0.001092
Synephrine	1.281708	0.000222
Medroxyprogesterone Acetate	0.946326	0.055375
Lecithin	0.887815	0.002453
Solanesol	0.960357	0.212502
2-Deoxy-2-sulfoamino-D-glucose sodium	0.911340	0.015113
Royal Jelly acid	0.755110	0.000272
Phosphocreatine disodium	1.270851	0.020721
4',7-Dimethoxyisoflavone	0.790699	0.000240

Calcium carbonate	1.251548	0.000277
Orotic acid zinc	1.108588	0.000900
Cinobufagin	0.852226	0.000229

Supplementary Table 2. Oligonucleotides used in this study.

Name		Sequence
mIfnb	Forward	5’-ATGAGTGGTGGTTGCAGGC-3’
mIfnb	Reverse	5’-TGACCTTTCAAATGCAGTAGATTCA-3’
mIfna4	Forward	5’-GACTTGTCTGCTACTTGGAATGC-3’
mIfna4	Reverse	5’-TTGGTTGAGGAAGAGAGGGCT-3’
mTnfa	Forward	5’-GCCACCACGTCTTCTGTCT-3’
mTnfa	Reverse	5’-TGAGGGTCTGGGCCATAGAAC-3’
mIl6	Forward	5’-ACAACCACGGCCTTCCCTAC-3’
mIl6	Reverse	5’-CATTTCCACGATTTCCCAGA-3’
mIfit2	Forward	5’-CCTAAACAGTTACTCCACCTTCG-3’
mIfit2	Reverse	5’-TTGCTGACCTCCTCCATTCT-3’
mIsg15	Forward	5’-AGAAGCAGATTGCCCAGAAG-3’
mIsg15	Reverse	5’-TGCGTCAGAAAGACCTCATAGA-3’
mCcl5	Forward	5’-AAGGAACCGCCAAGTGTGTG-3’
mCcl5	Reverse	5’-CAGGACCGAGTGGGAGTAGG-3’
mCxcl10	Forward	5’-GACCTTTTTTGGCTAAACGCTTTC-3’
mCxcl10	Reverse	5’-ATCATCCCTGCGAGCCTATCCT-3’
mβ-Actin	Forward	5’-TGTTACCAACTGGGACGAC-3’
mβ-Actin	Reverse	5’-CTGGGTCATCTTTTCACGGT-3’
hIFNB	Forward	5′-ACGCCGCATTGACCATCTAT-3′

hIFNB	Reverse	5′-GTCTCATTCCAGCCAGTGCTA-3′
hACTIN	Forward	5′-GAAGAGCTACGAGCTGCCTGA-3′
hACTIN	Reverse	5′-CAGACAGCACTGTGTTGGCG-3′
Ctrl siRNA	Control	5′- UUCUCCGAACGUGUCACGU-3’
Sat1 siRNA	siRNA	5’- CUGGCUAAAUAUGAAUACA-3’
