# Supplementary material for: Polyamine sequestration of 2′3′-cGAMP constrains intercellular transmission and STING engagement to subvert antitumor immunity
Source: J Clin Invest. 2026 Jun 1;136(11):e201460. doi: 10.1172/JCI201460 (PMC13221228; doi:10.1172/JCI201460)

Full unedited blot/gel for Figure 1L

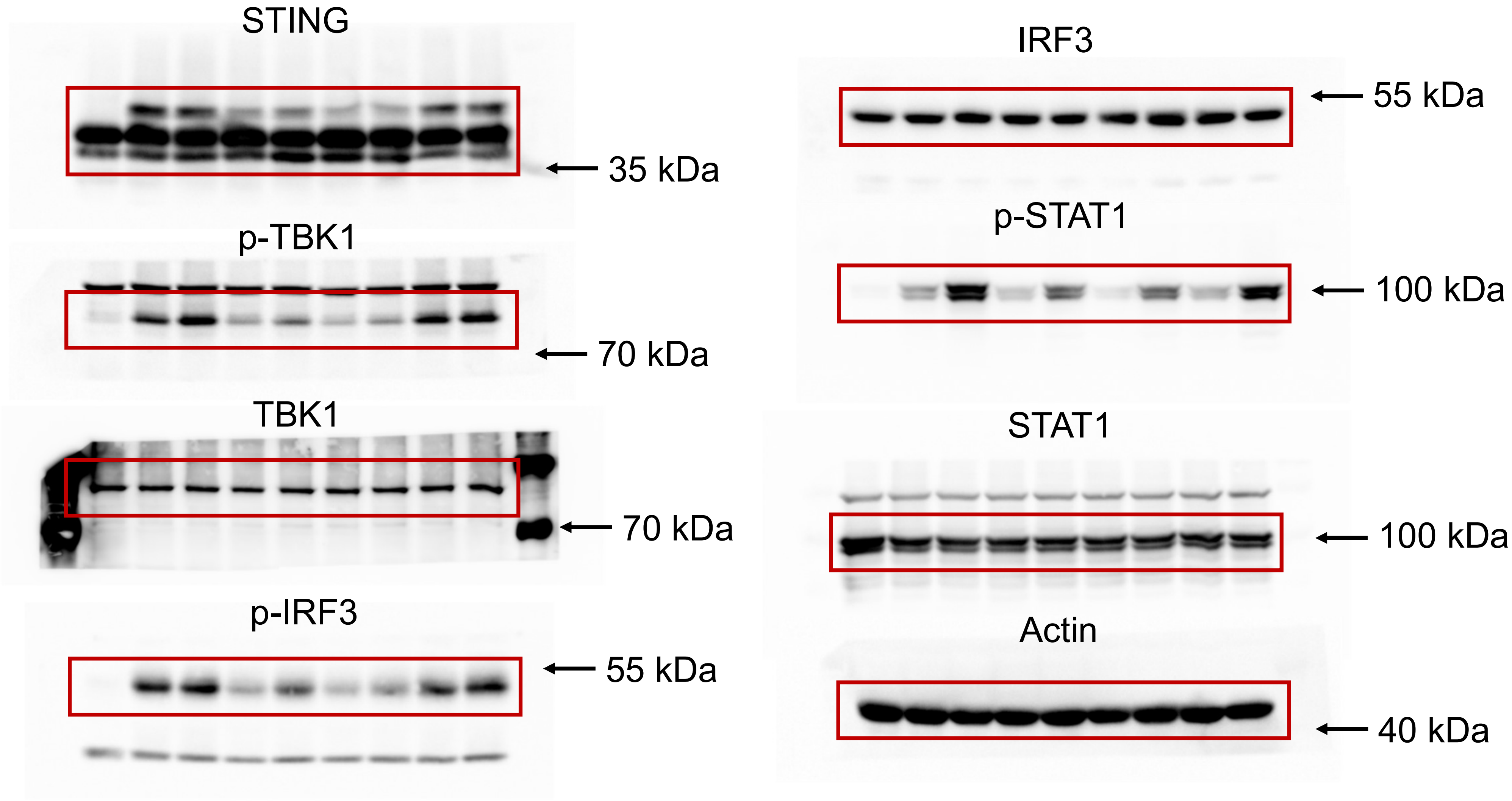

Full unedited blot/gel Figure 1Q

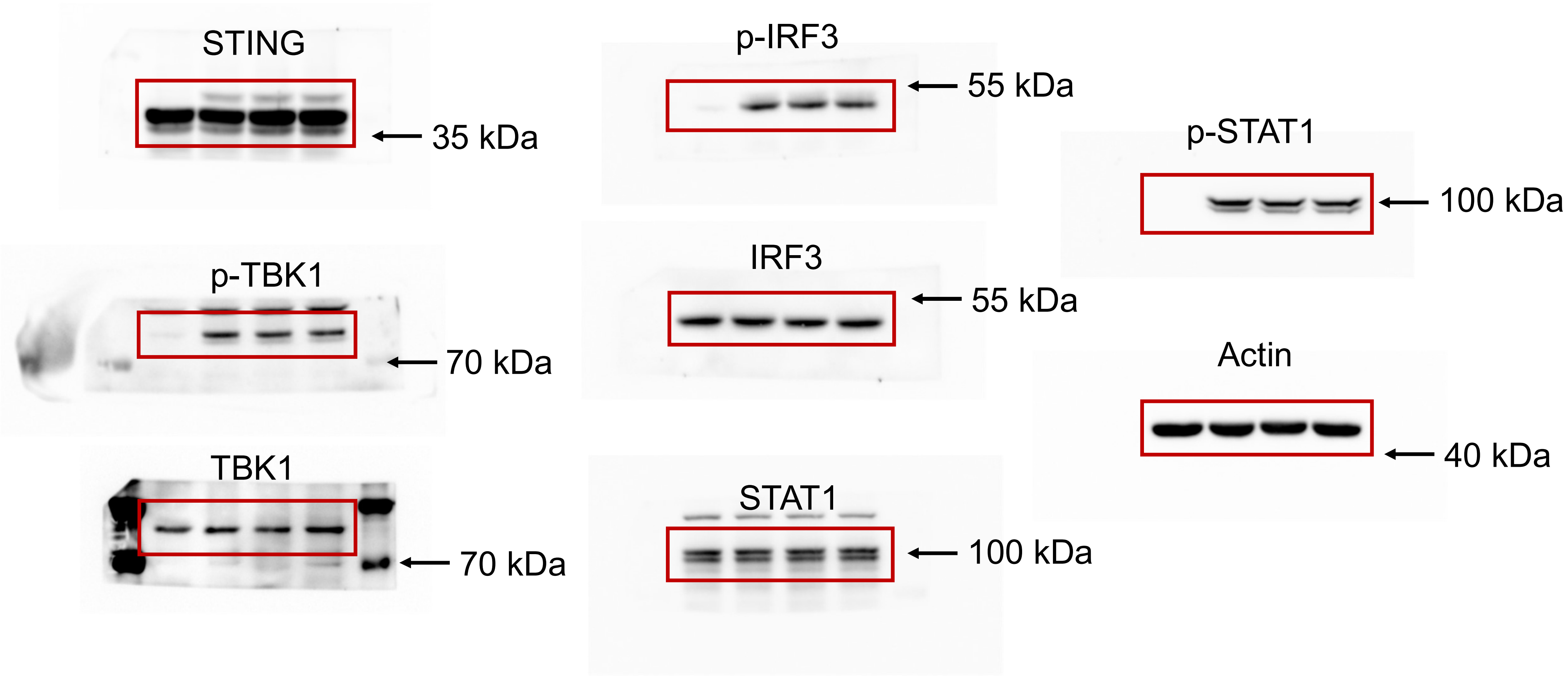

Full unedited blot/gel for Figure 2G

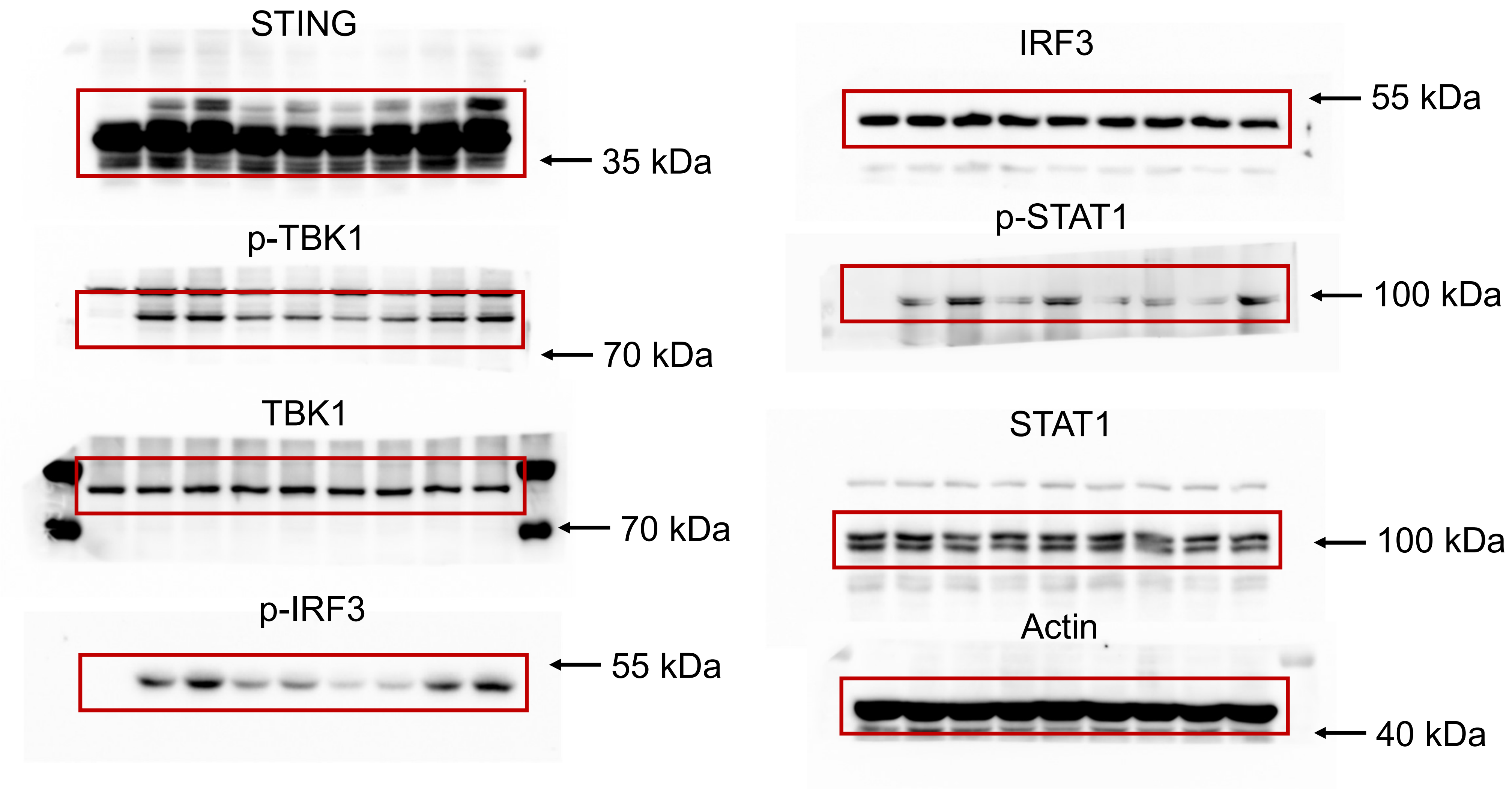

Full unedited blot/gel for Figure 2I

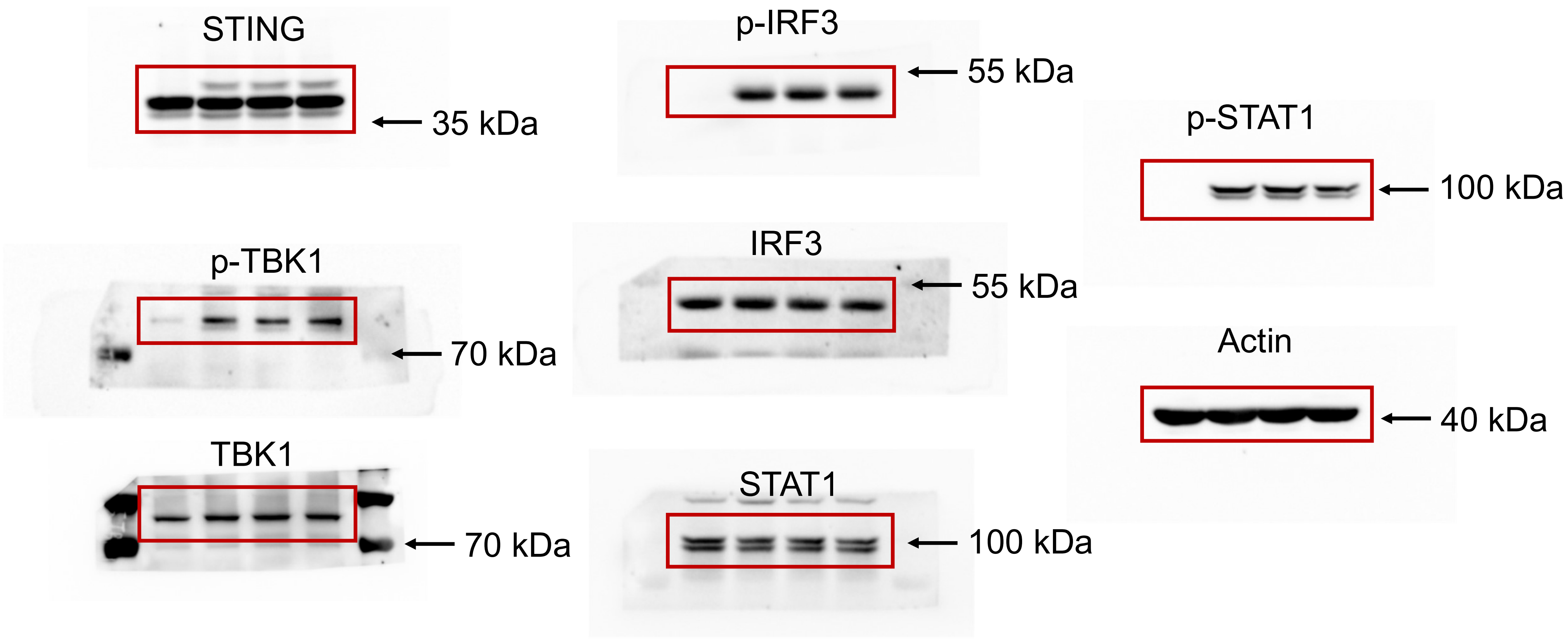

Full unedited blot/gel for Figure 2O

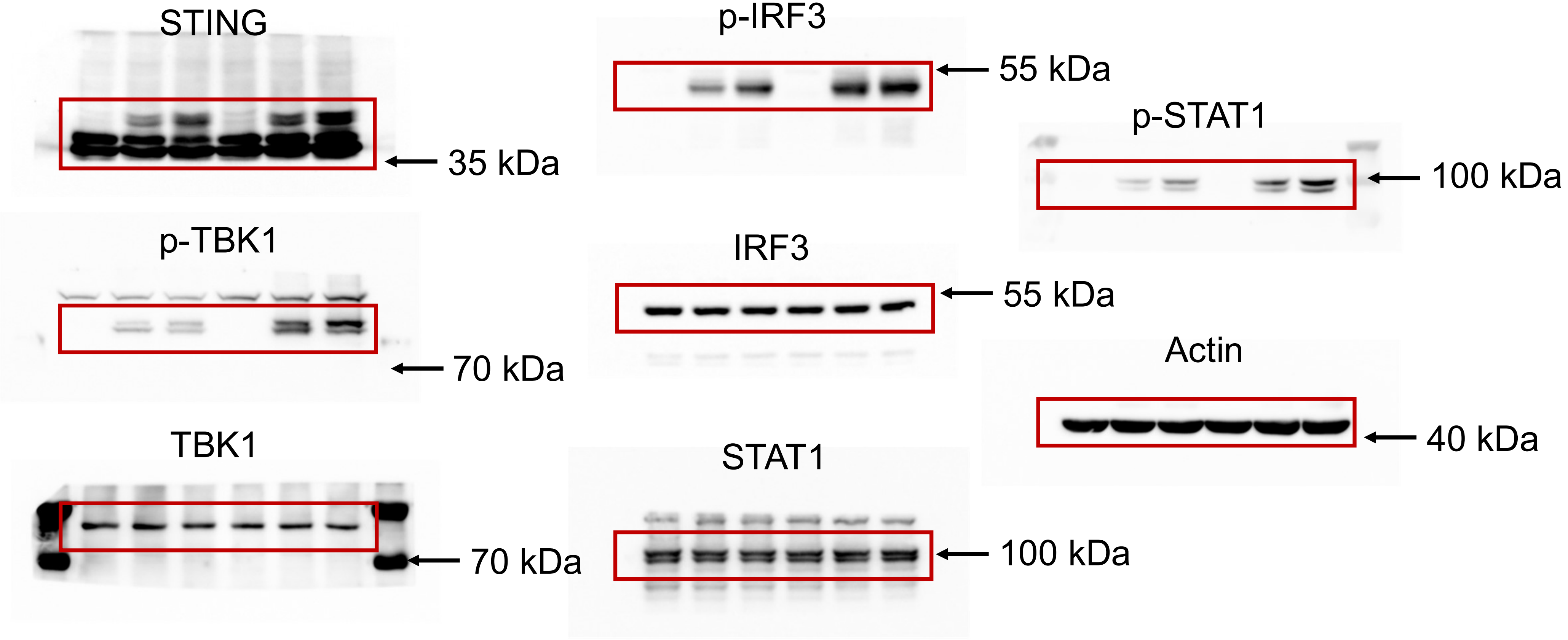

Full unedited blot/gel for Figure 3B

Spm

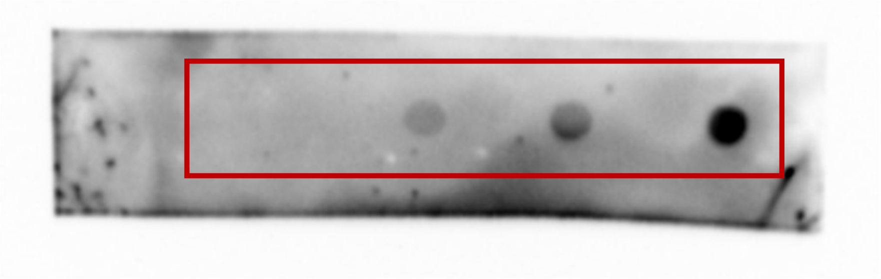

Spd

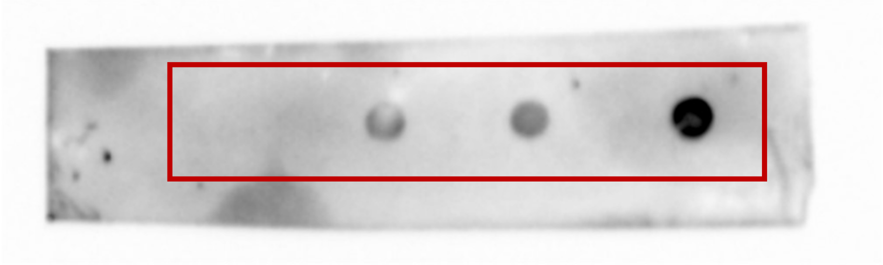

Full unedited blot/gel for Figure 4A

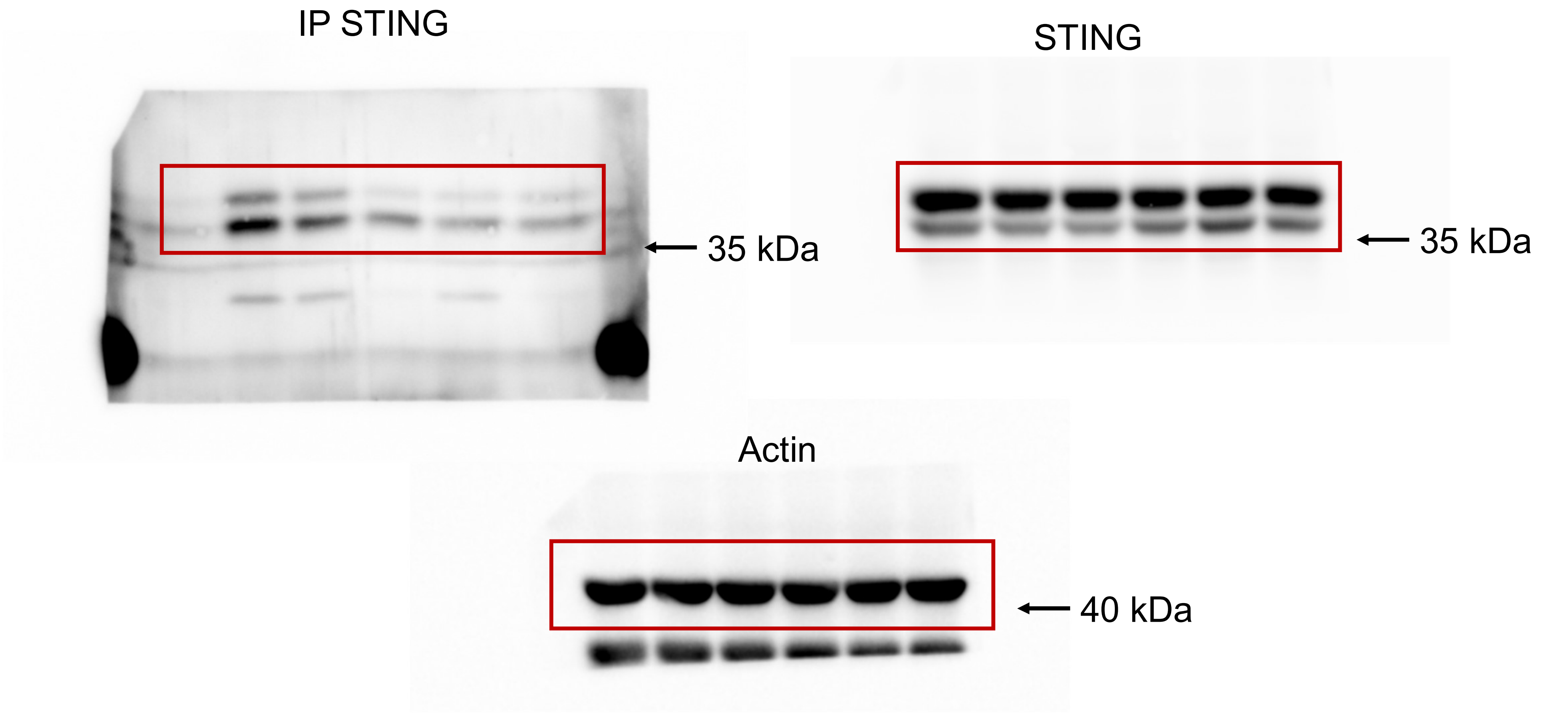

Full unedited blot/gel for Figure 4B

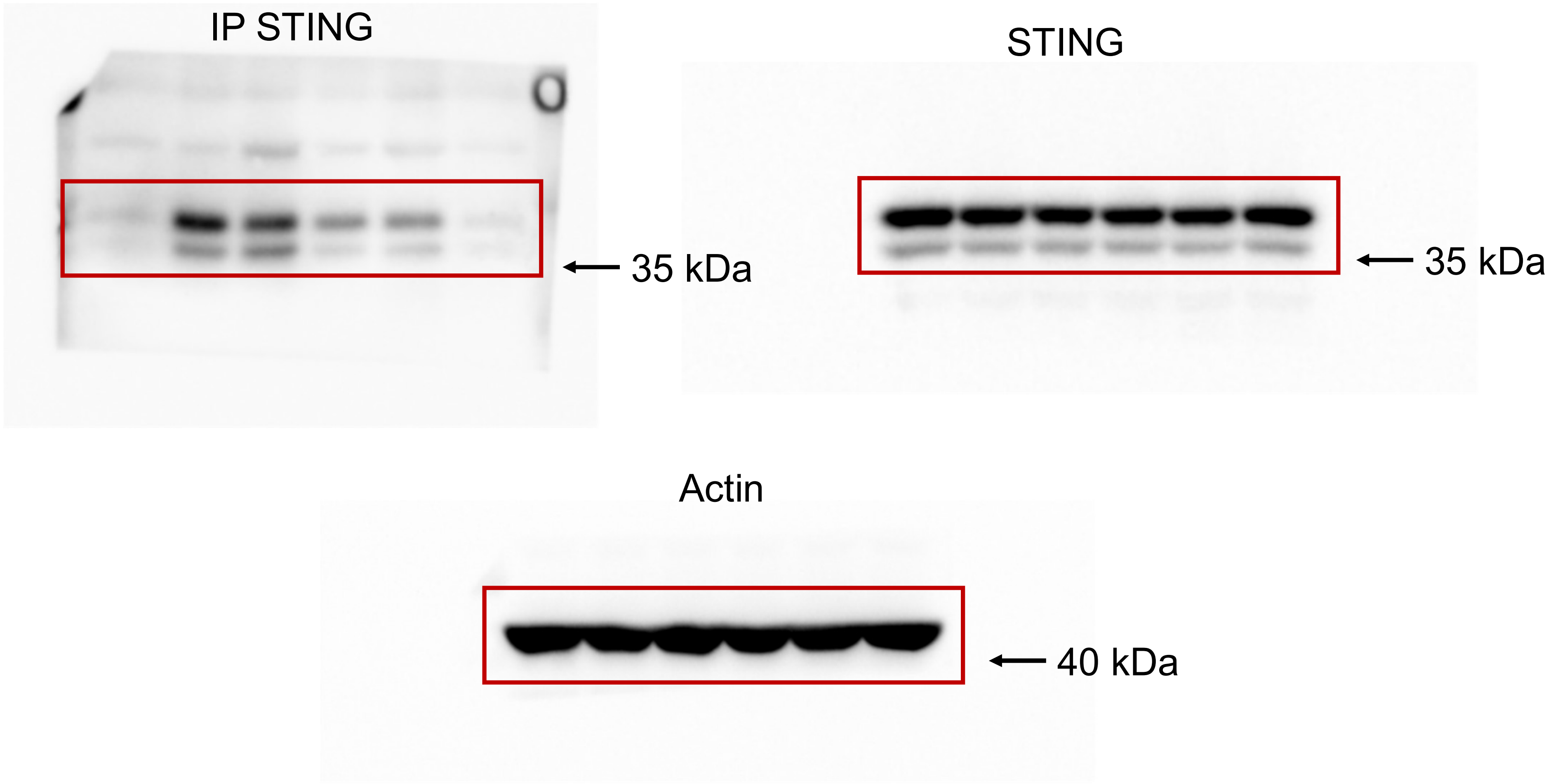

Full unedited blot/gel for Figure 4C

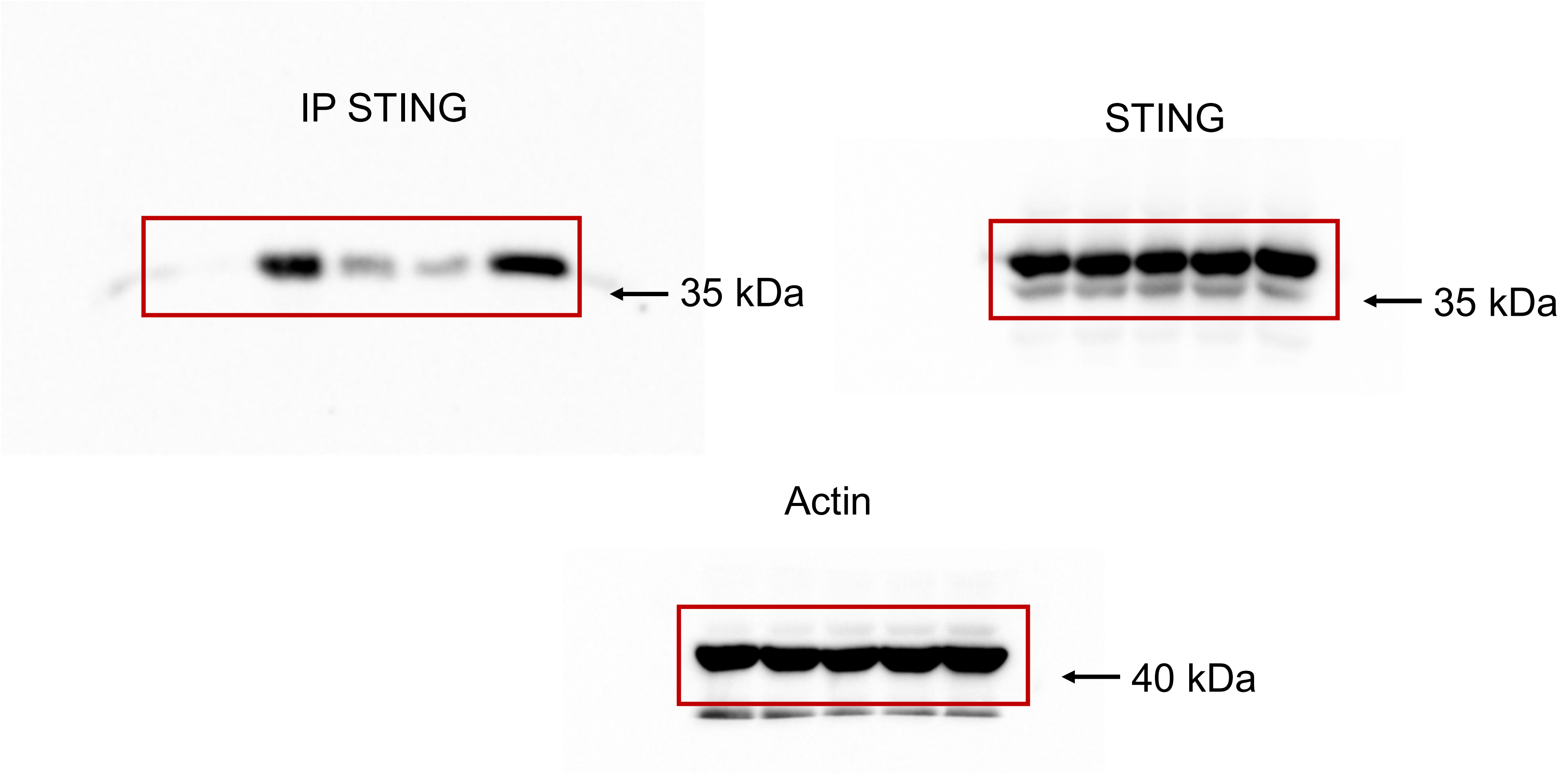

Full unedited blot/gel for Figure 4D

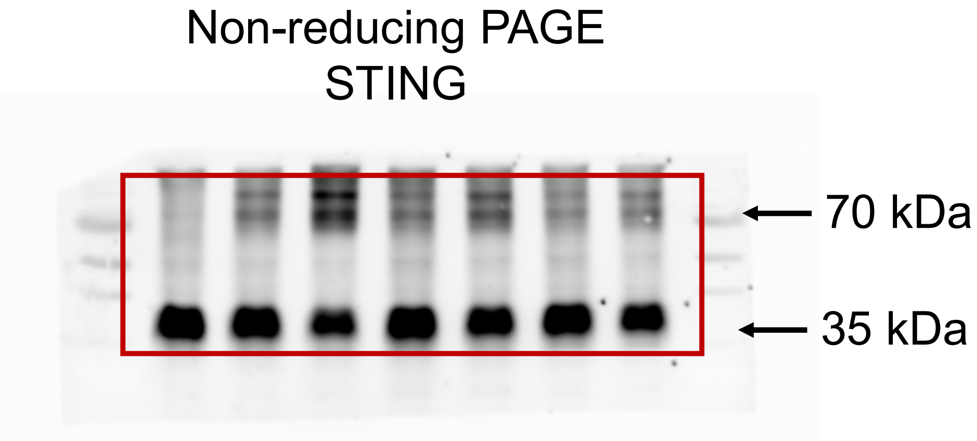

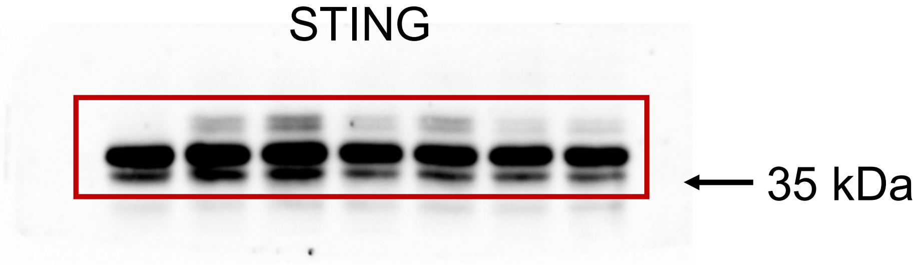

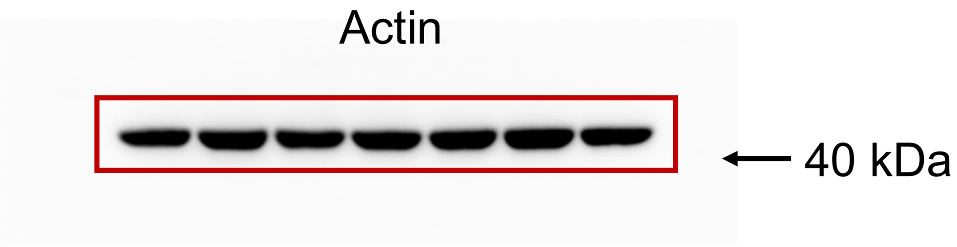

Full unedited blot/gel for Figure 4L

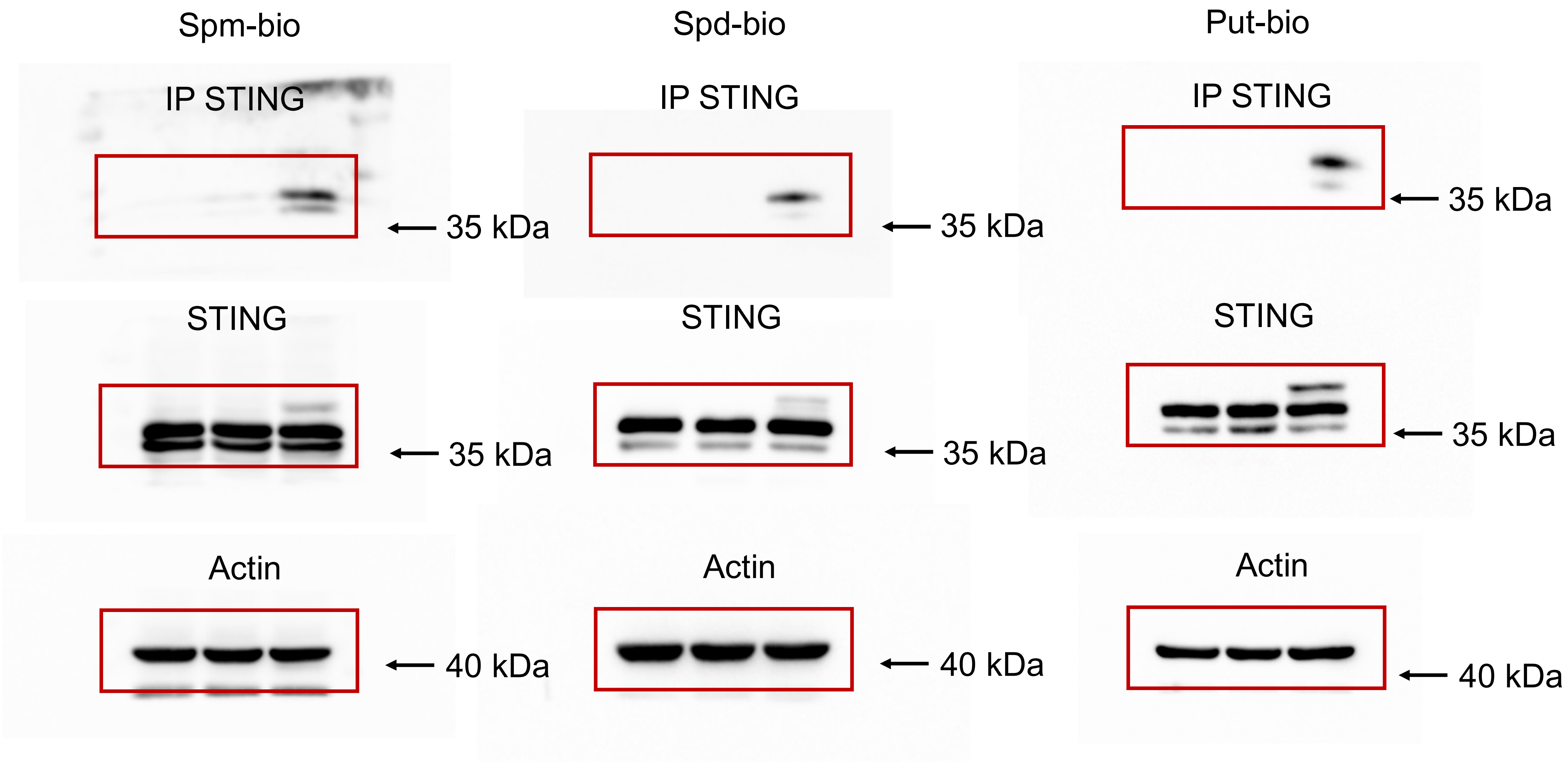

Full unedited blot/gel for Figure 5A

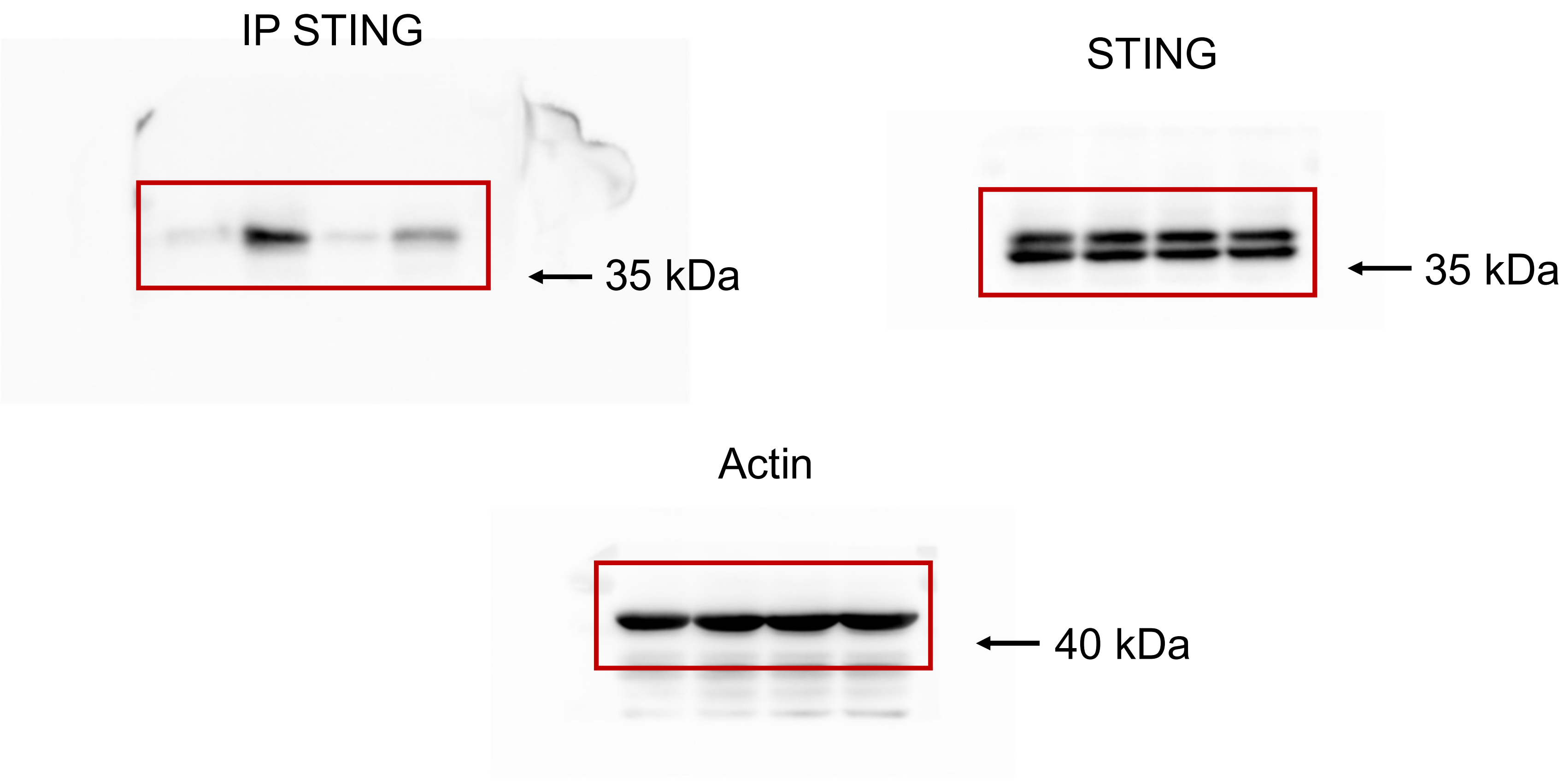

Full unedited blot/gel for Figure 5B

Non-reducing PAGE
STING

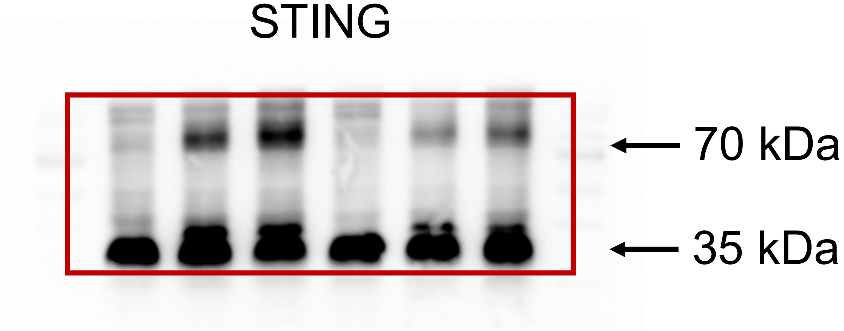

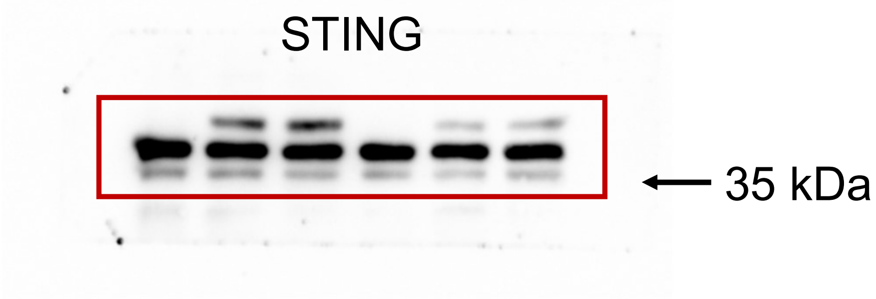

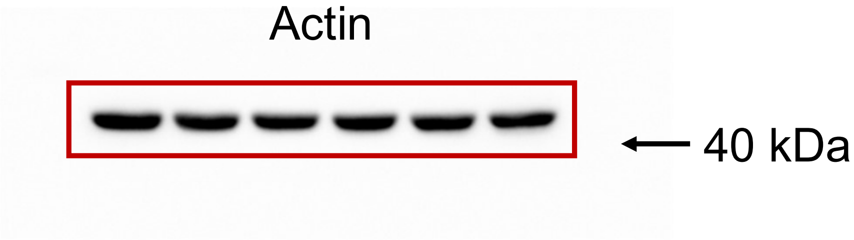

Full unedited blot/gel for Figure 5C

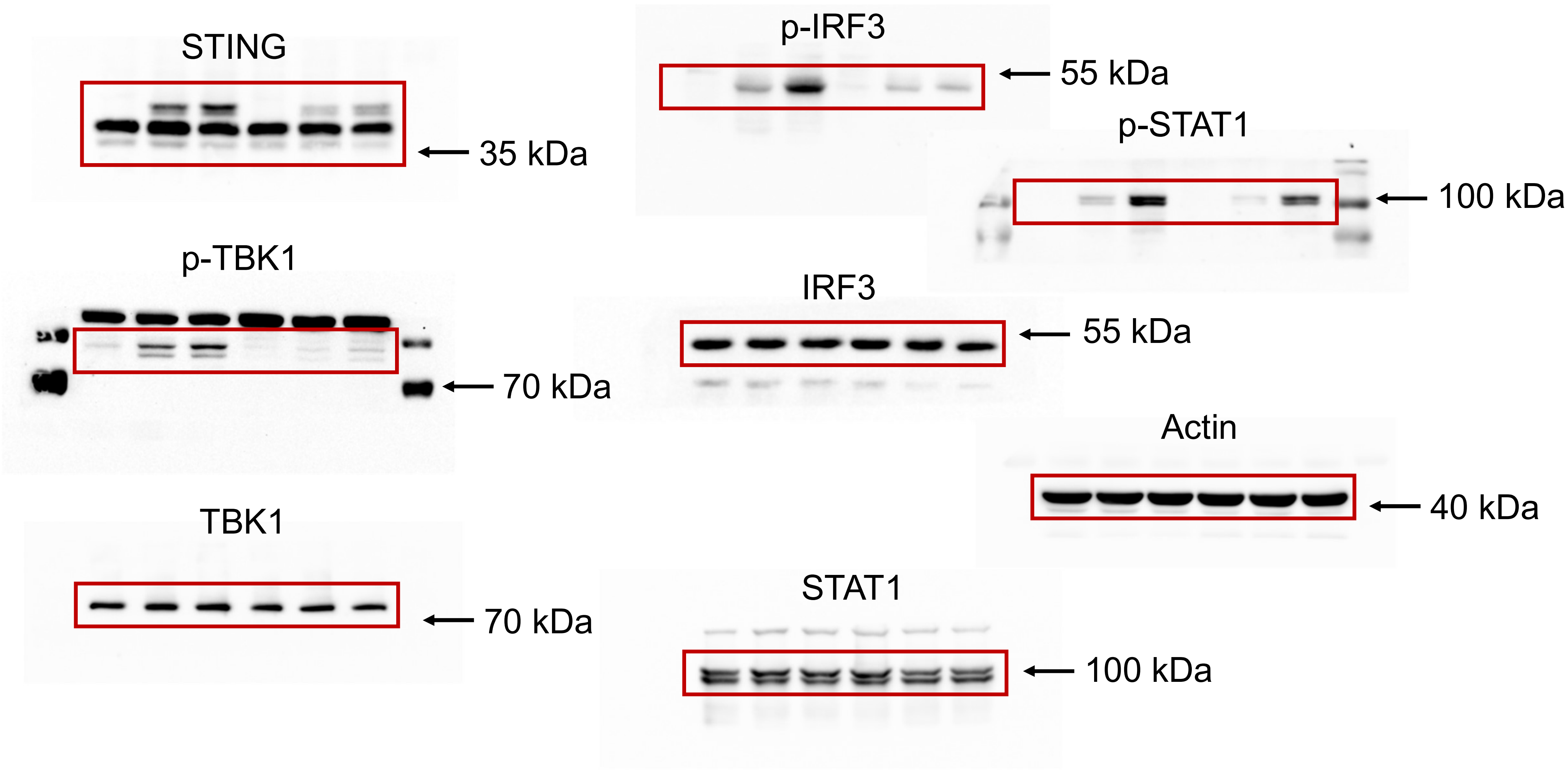

Full unedited blot/gel for Figure 5G

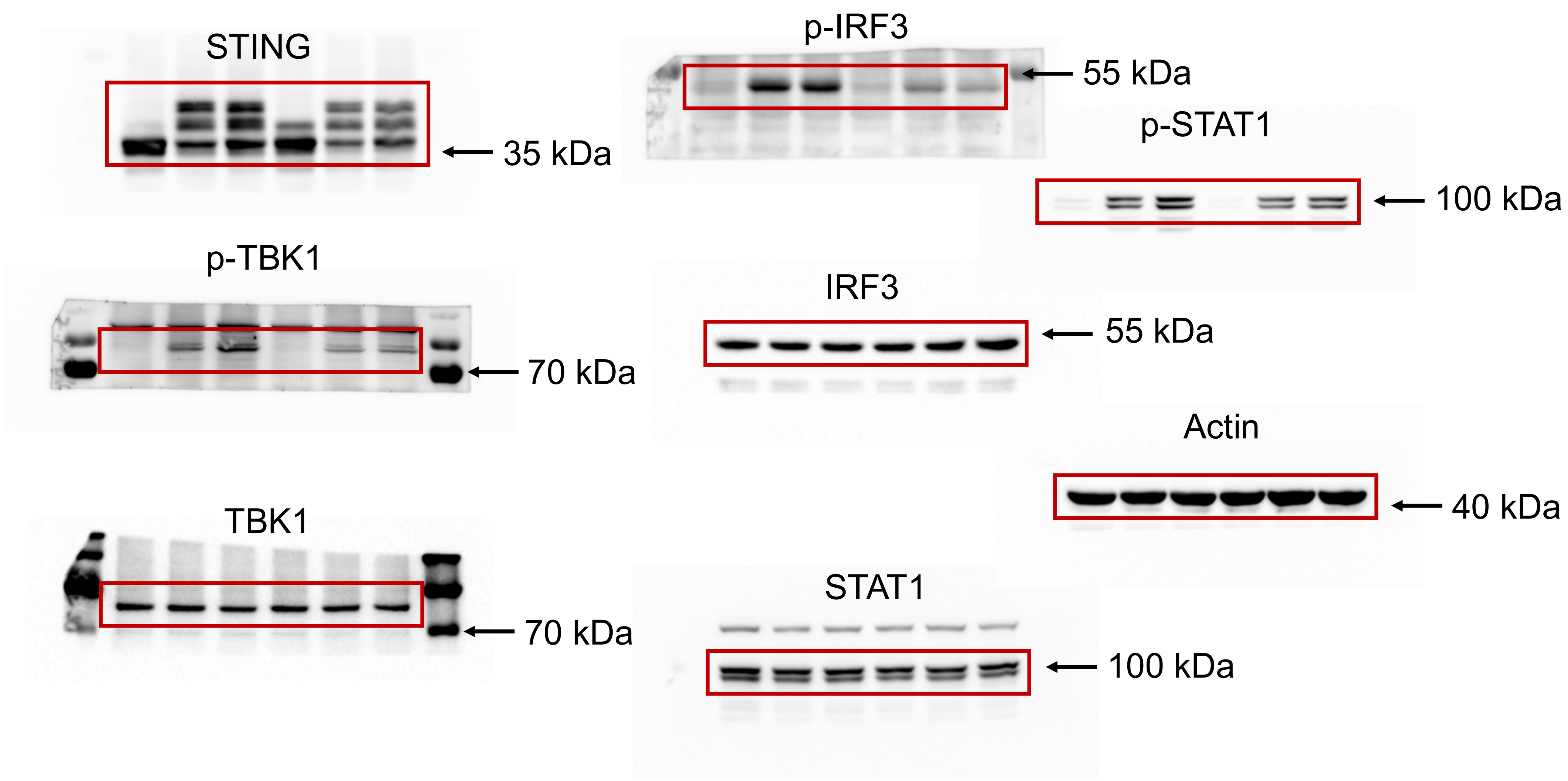

Full unedited blot/gel for Figure 5J

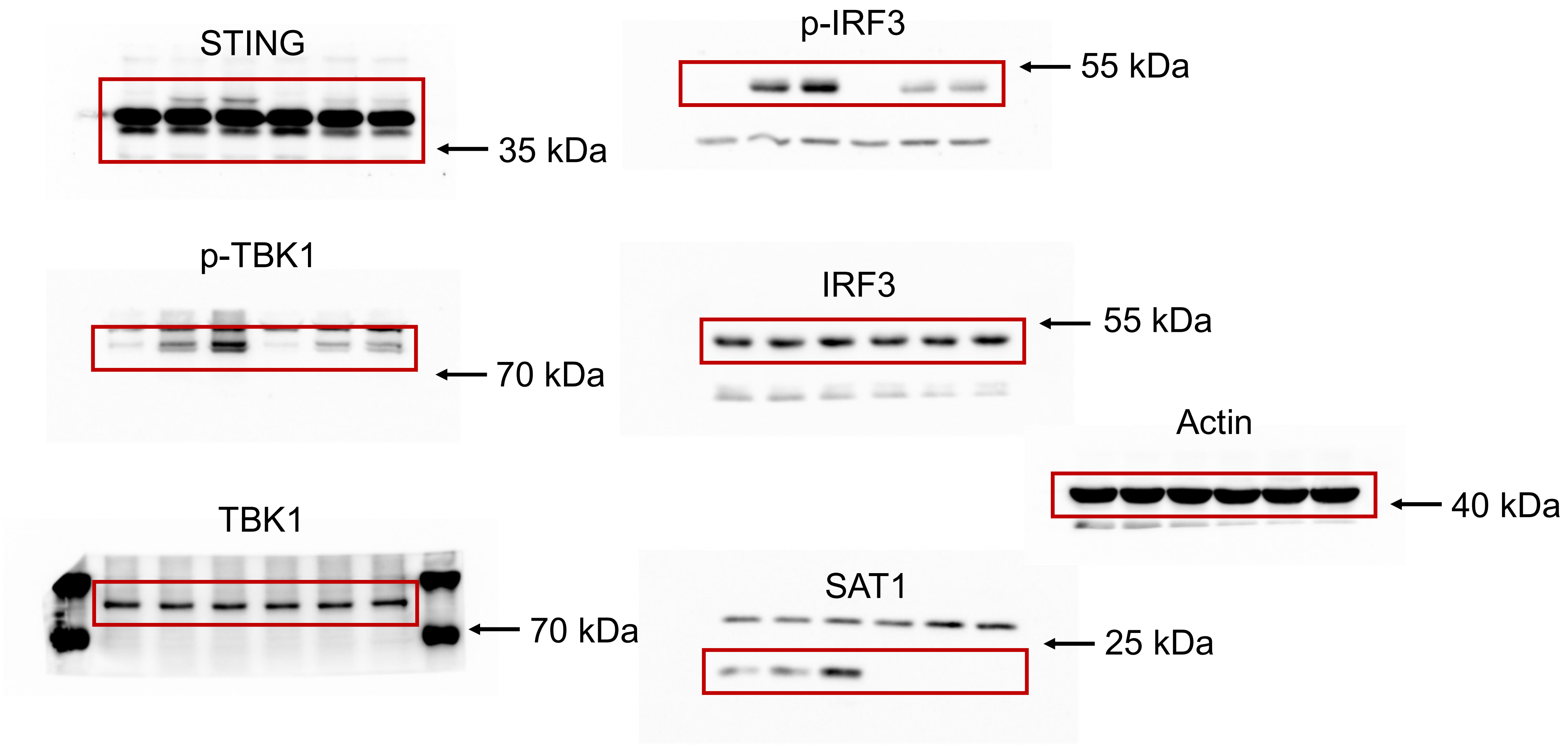

Full unedited blot/gel for Supplementary Figure 1F

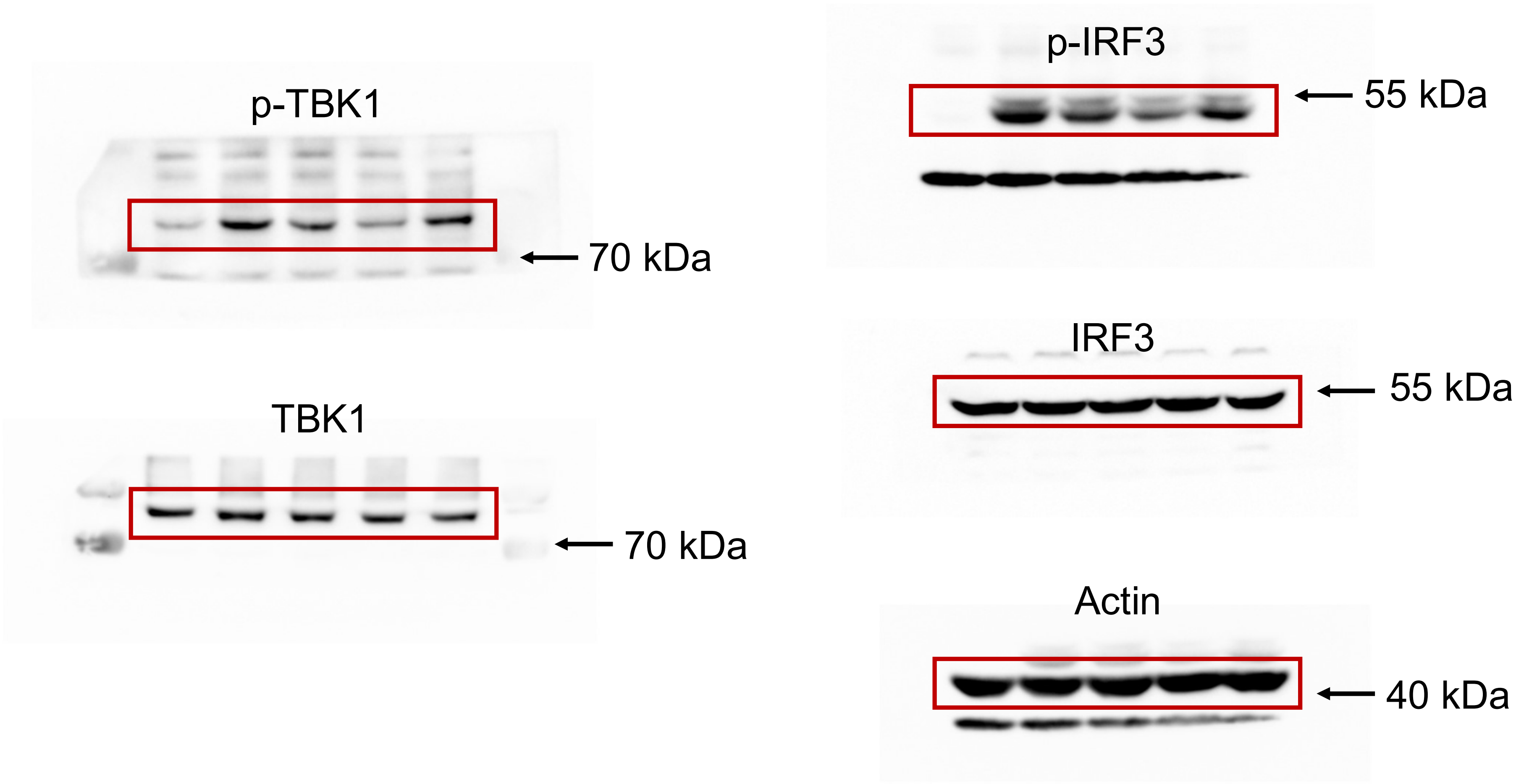

Full unedited blot/gel for Supplementary Figure 1G

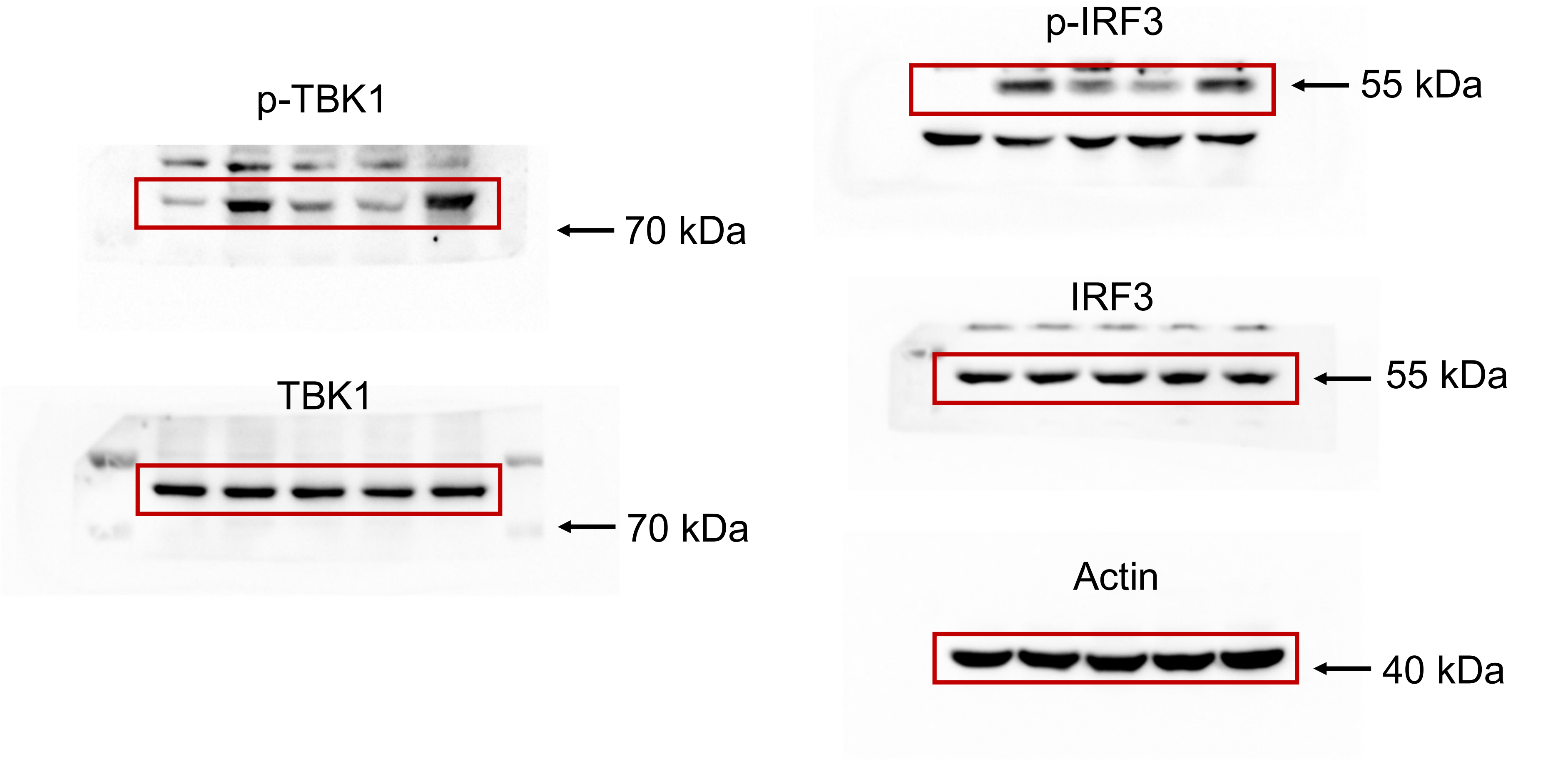

Full unedited blot/gel for Supplementary Figure 1H

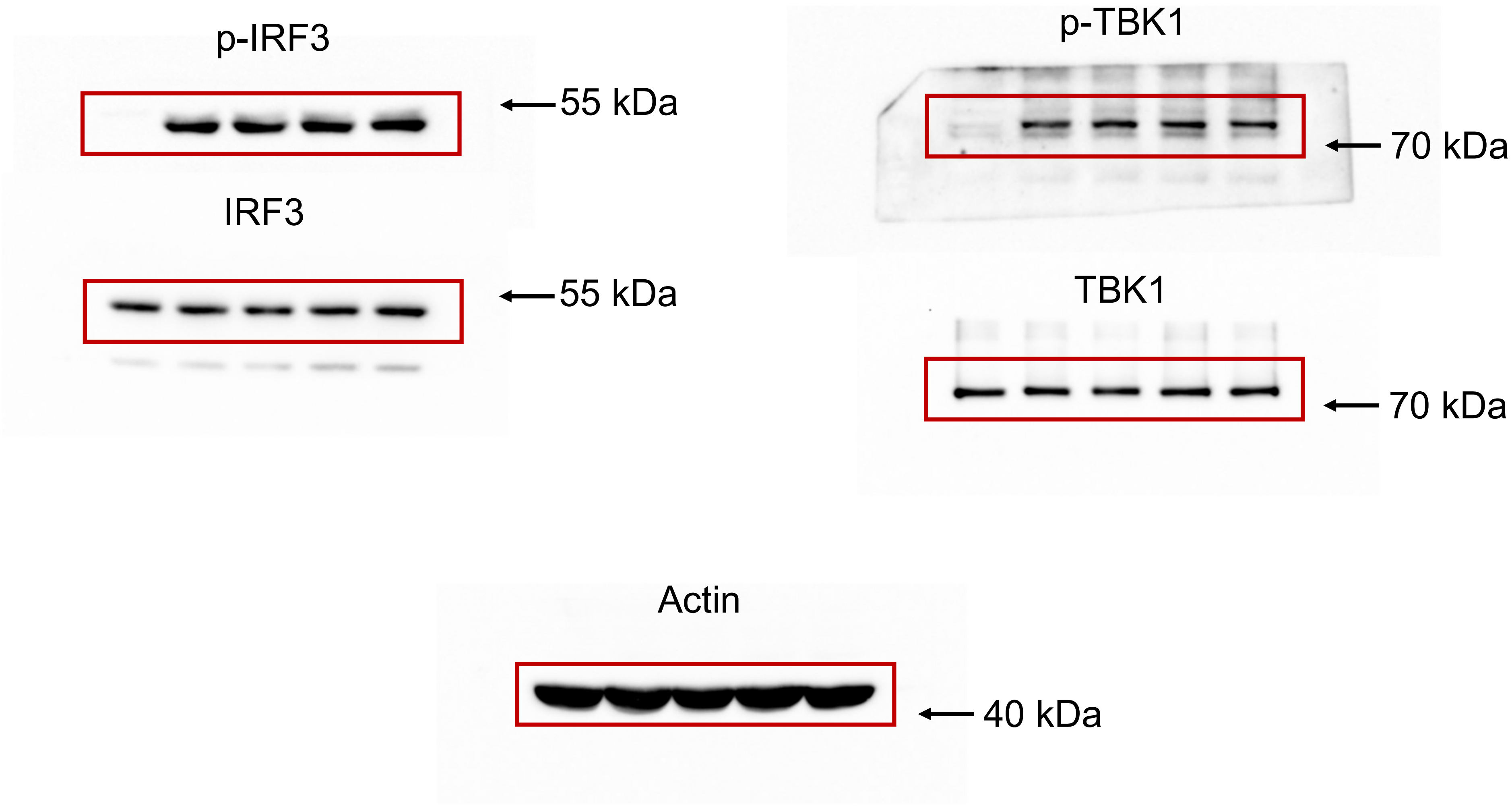

Full unedited blot/gel for Supplementary Figure 1I

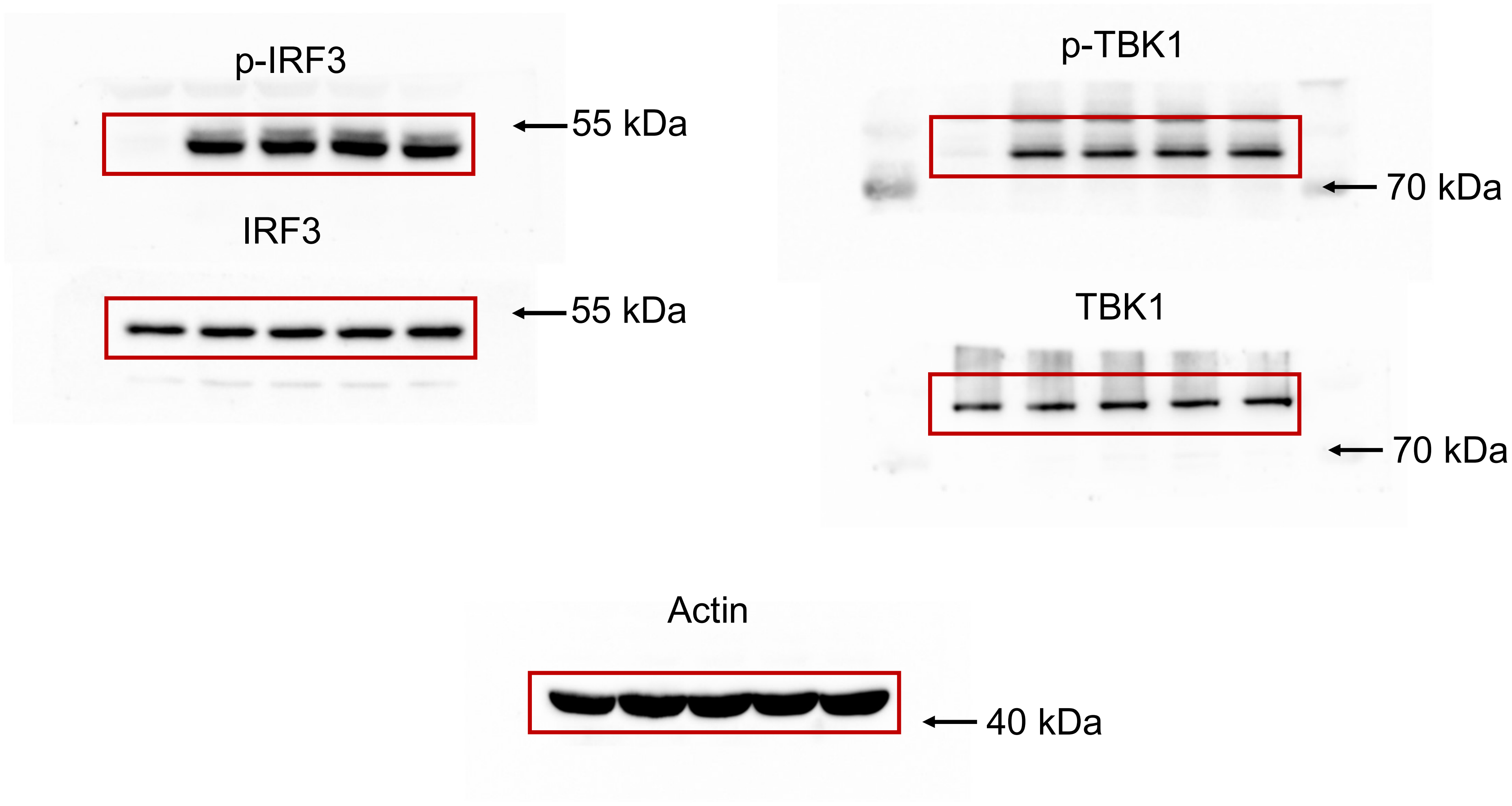

Full unedited blot/gel for Supplementary Figure 1J

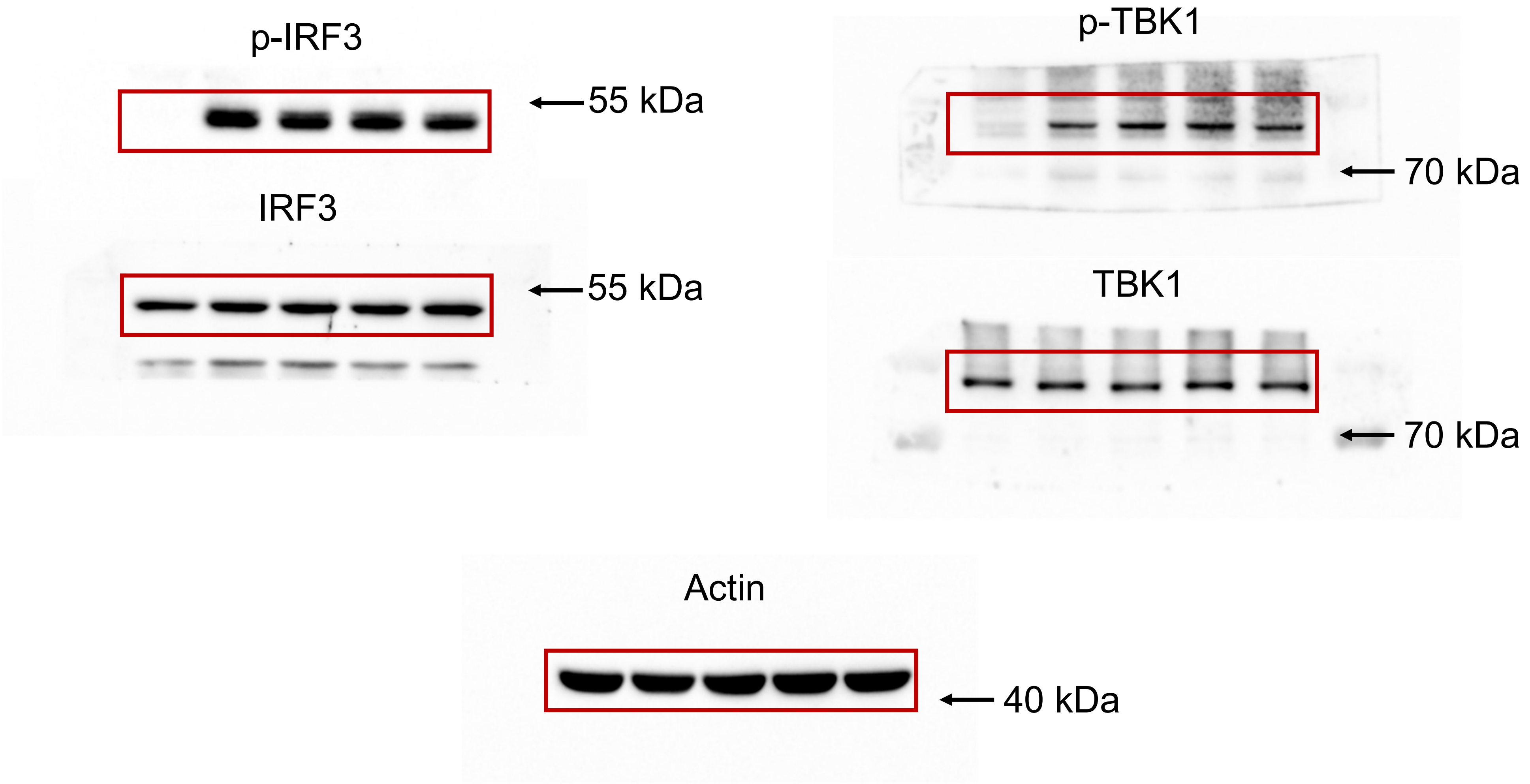

Full unedited blot/gel for Supplementary Figure 2F

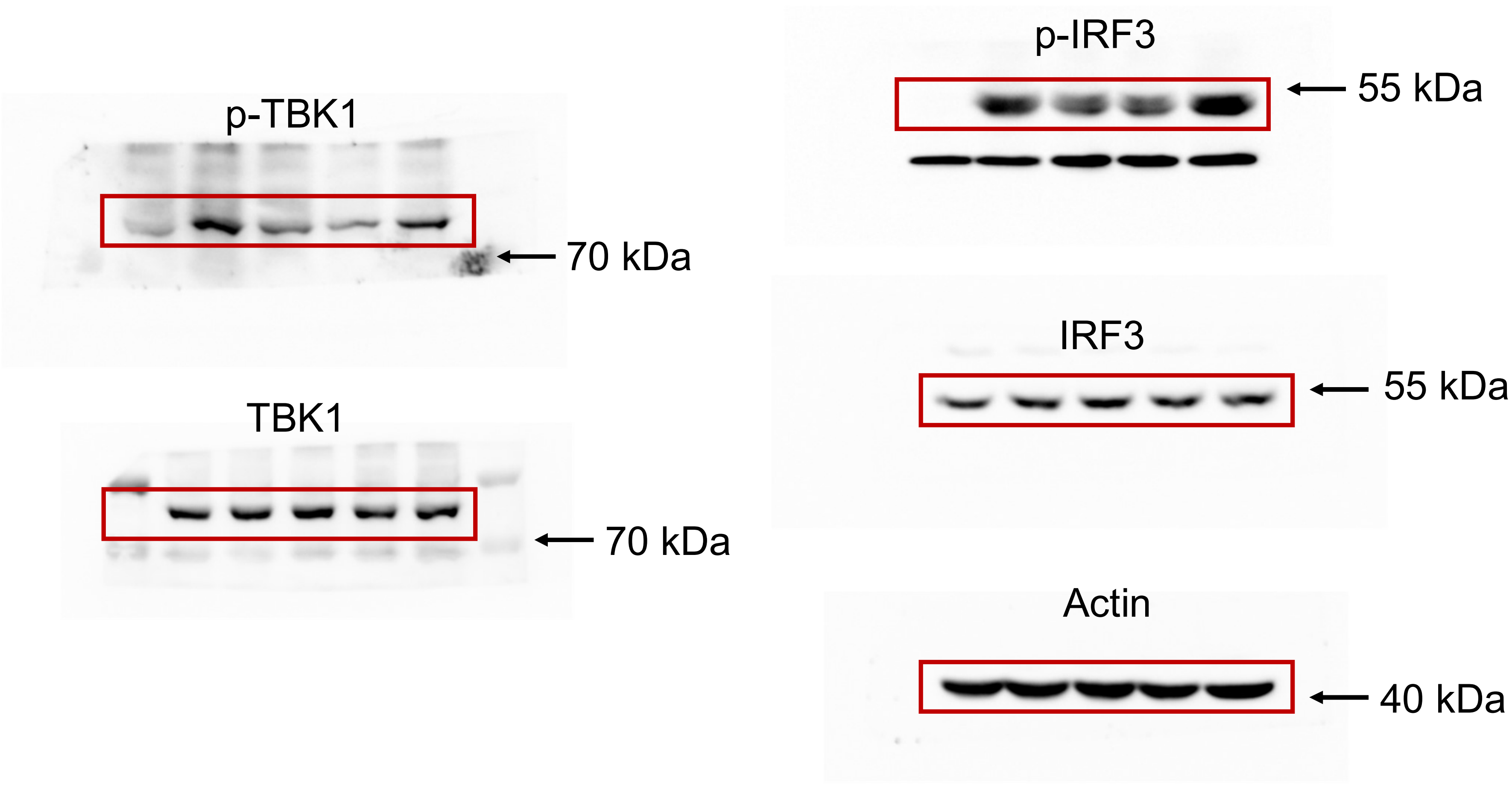

Full unedited blot/gel for Supplementary Figure 2G

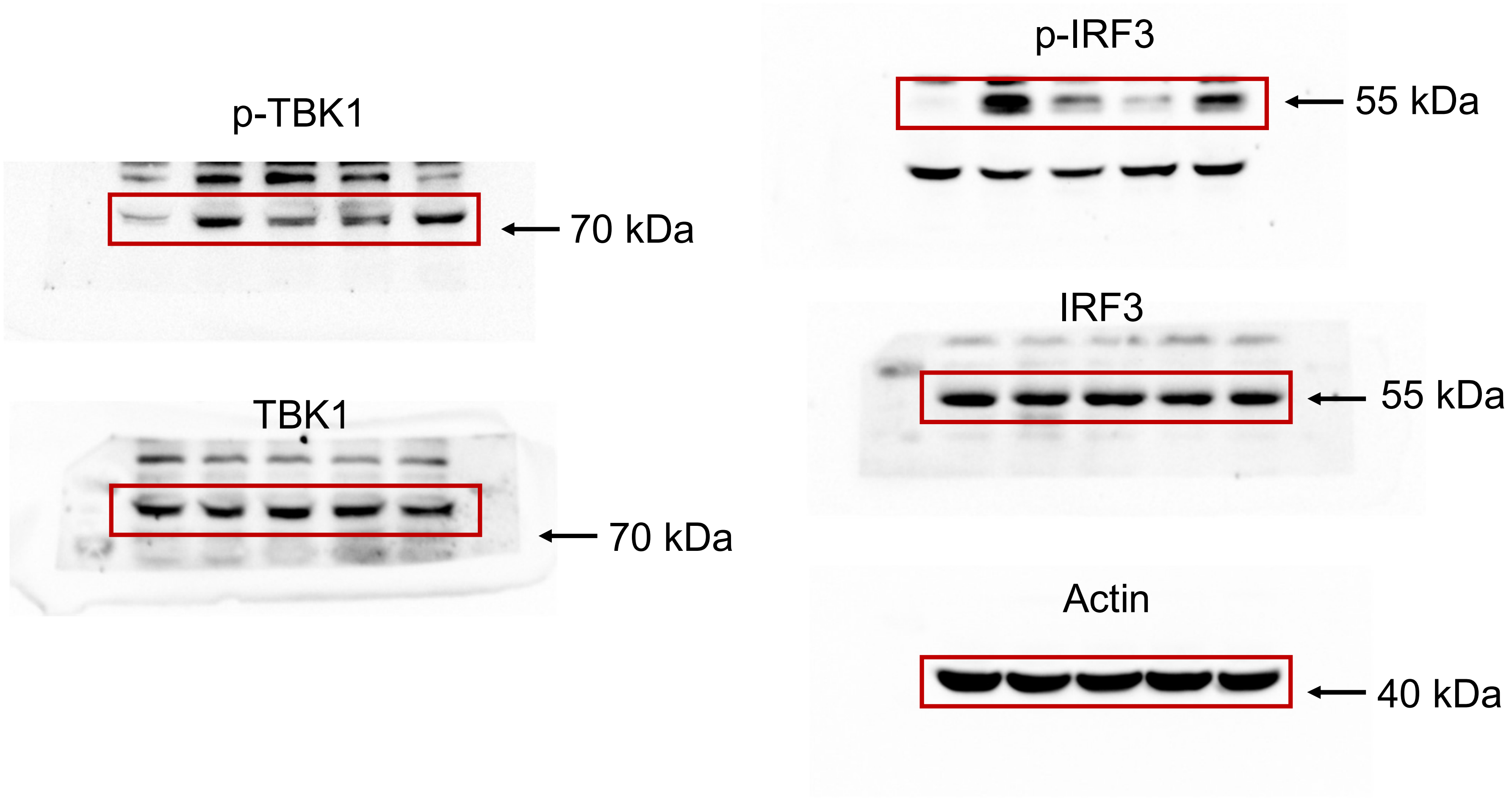

Full unedited blot/gel for Supplementary Figure 2J

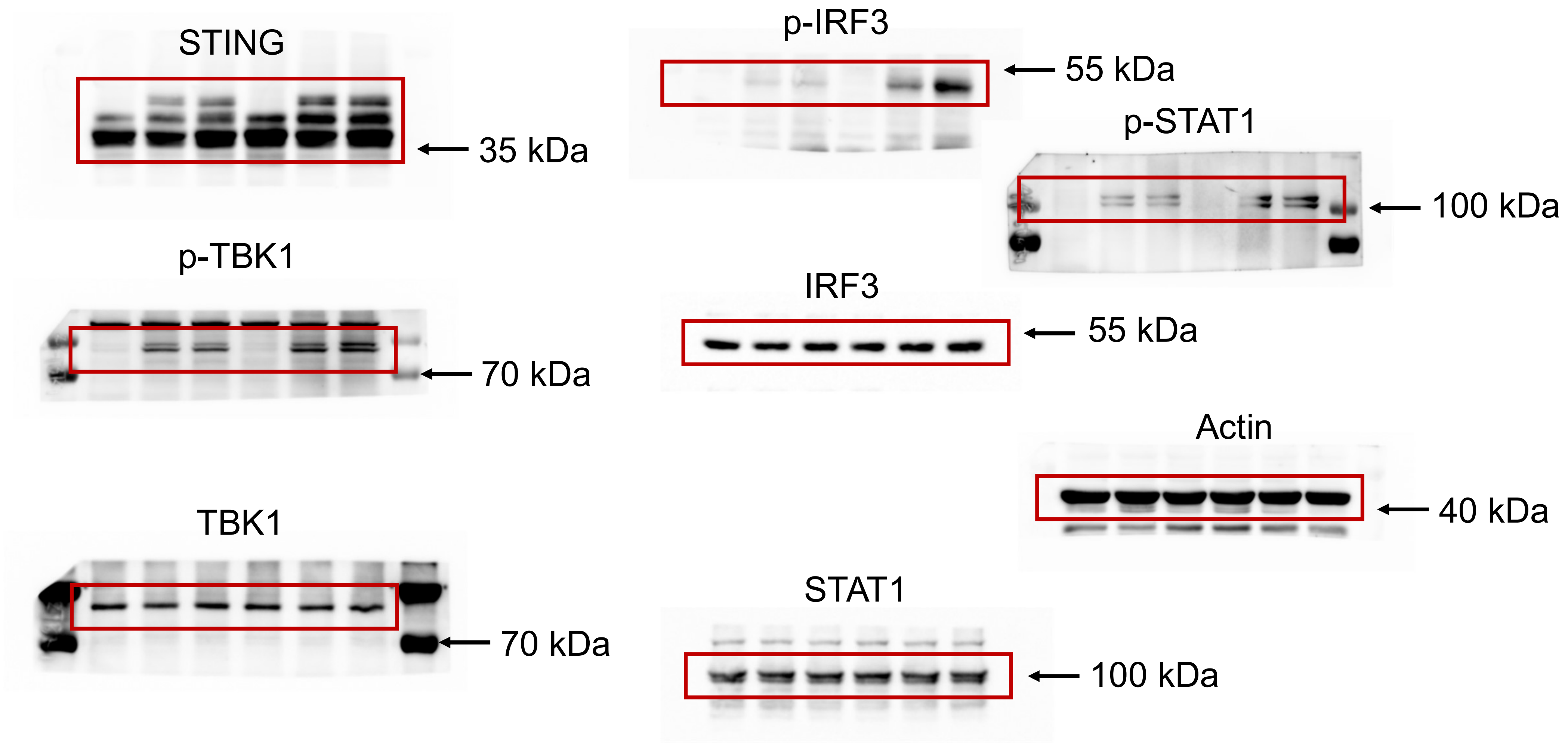

Full unedited blot/gel for Supplementary Figure 3E

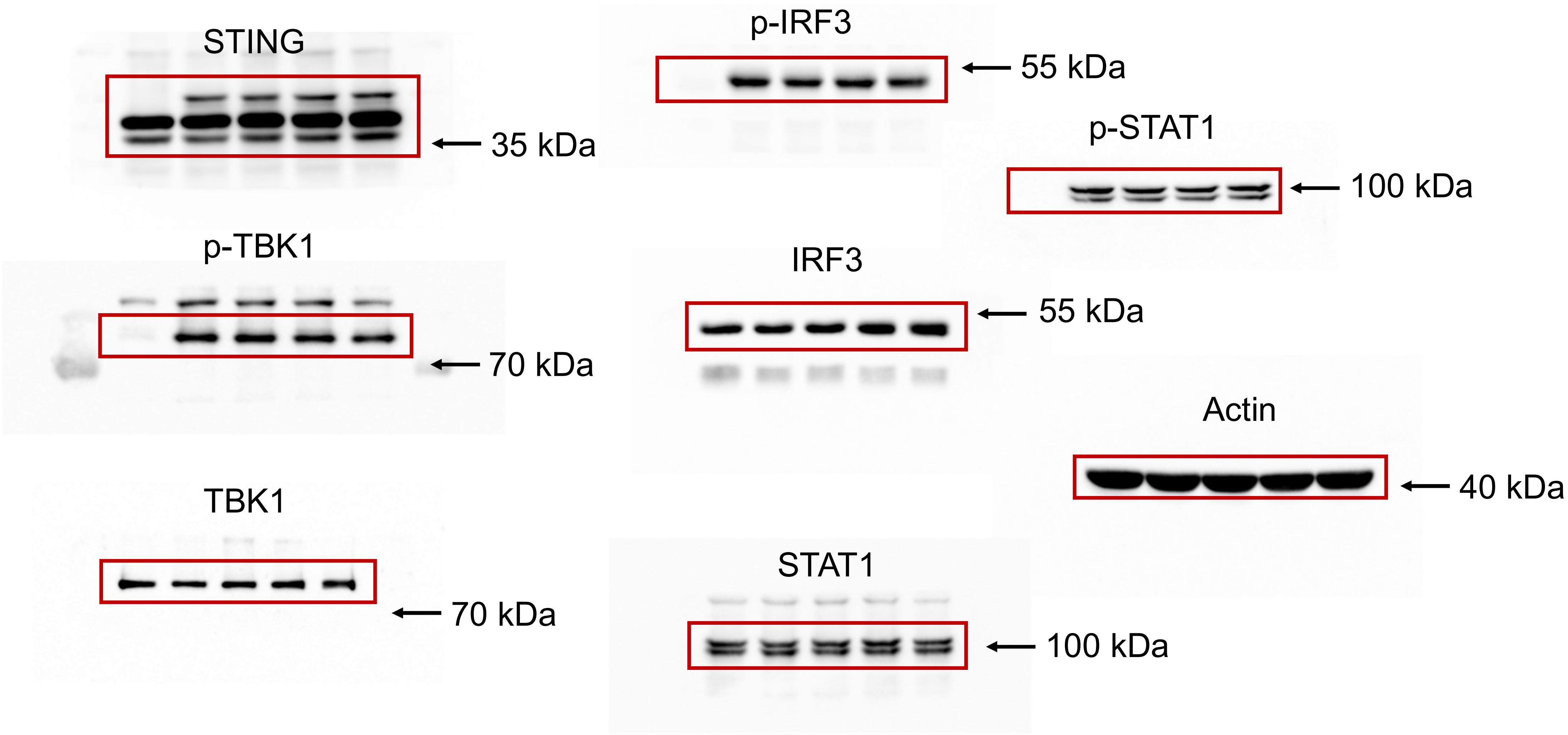

Full unedited blot/gel for Supplementary Figure 3F

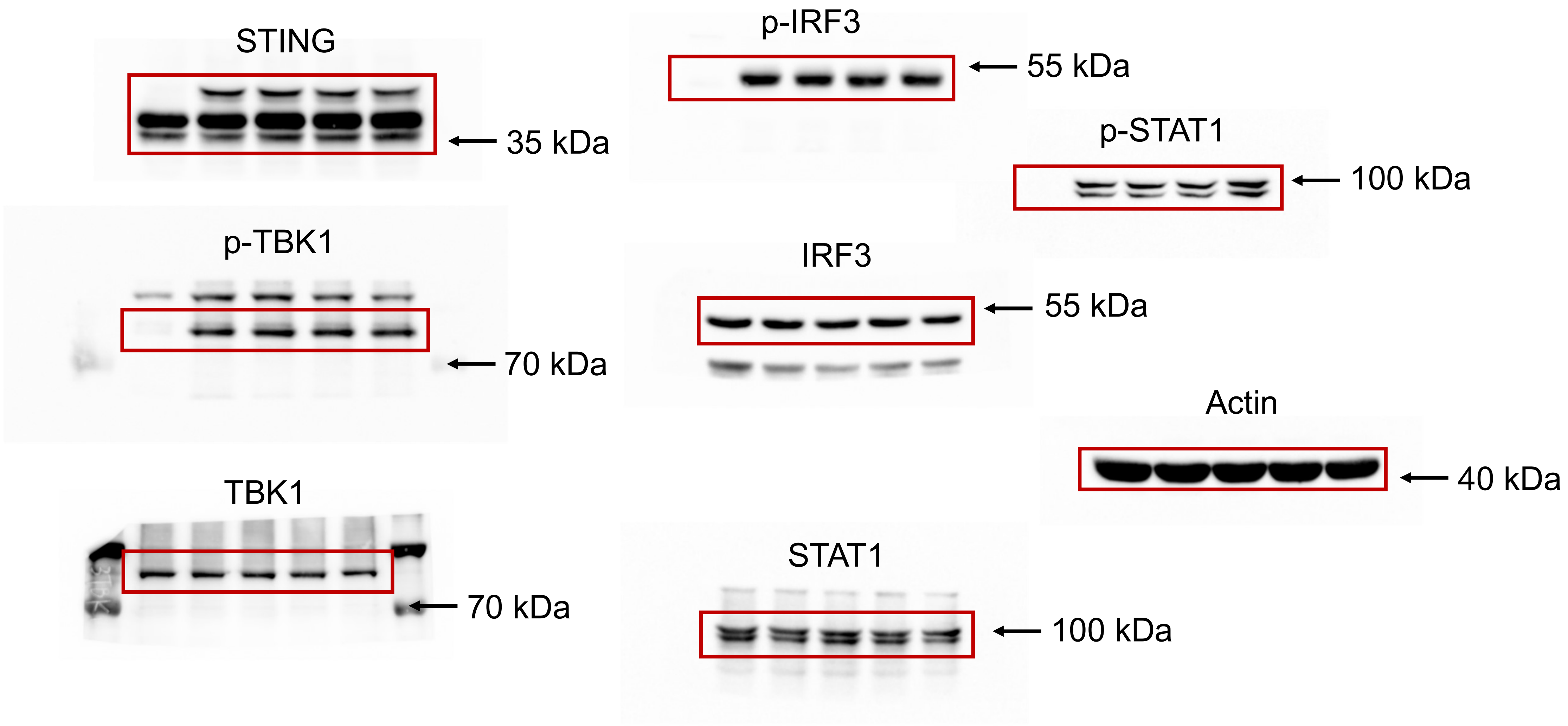

Full unedited blot/gel for Supplementary Figure 3G

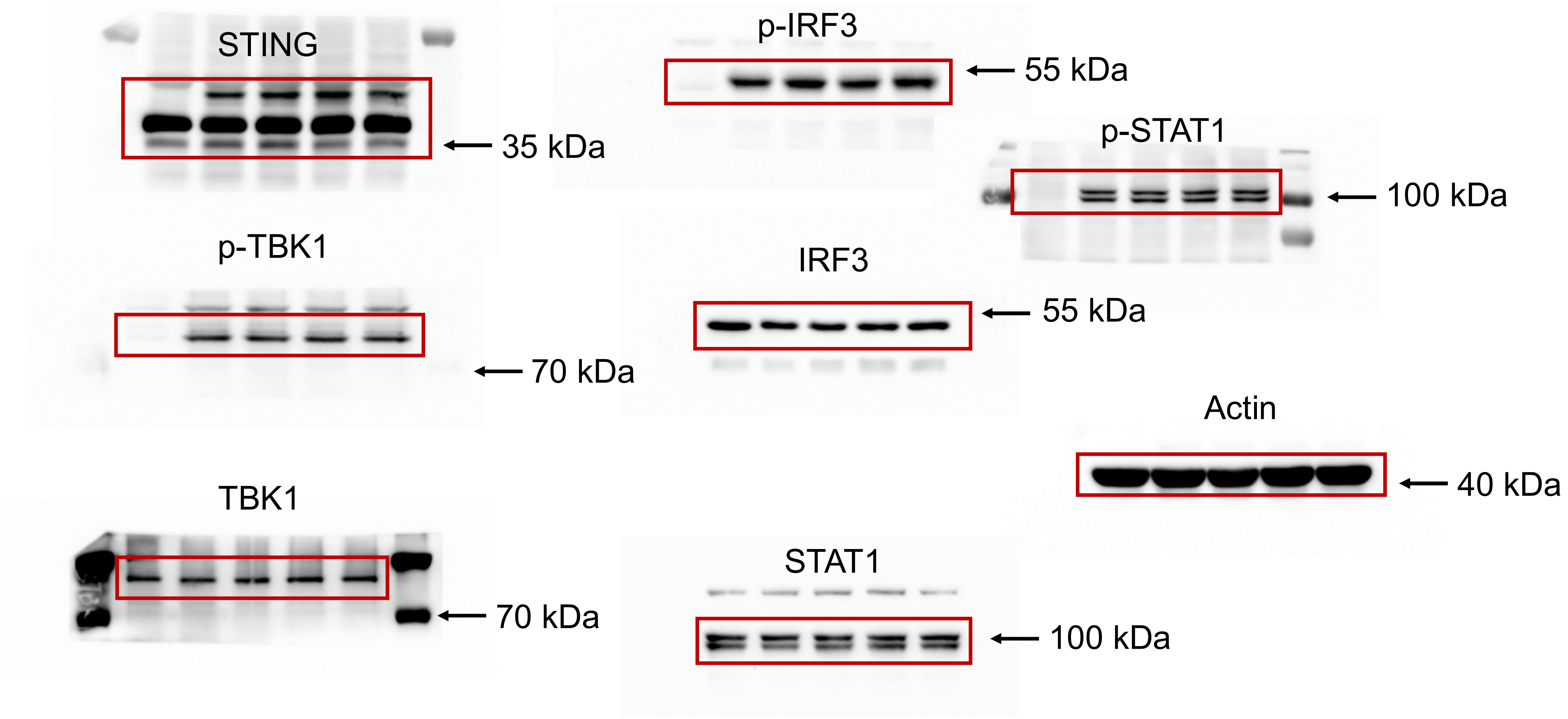

Full unedited blot/gel for Supplementary Figure 3H

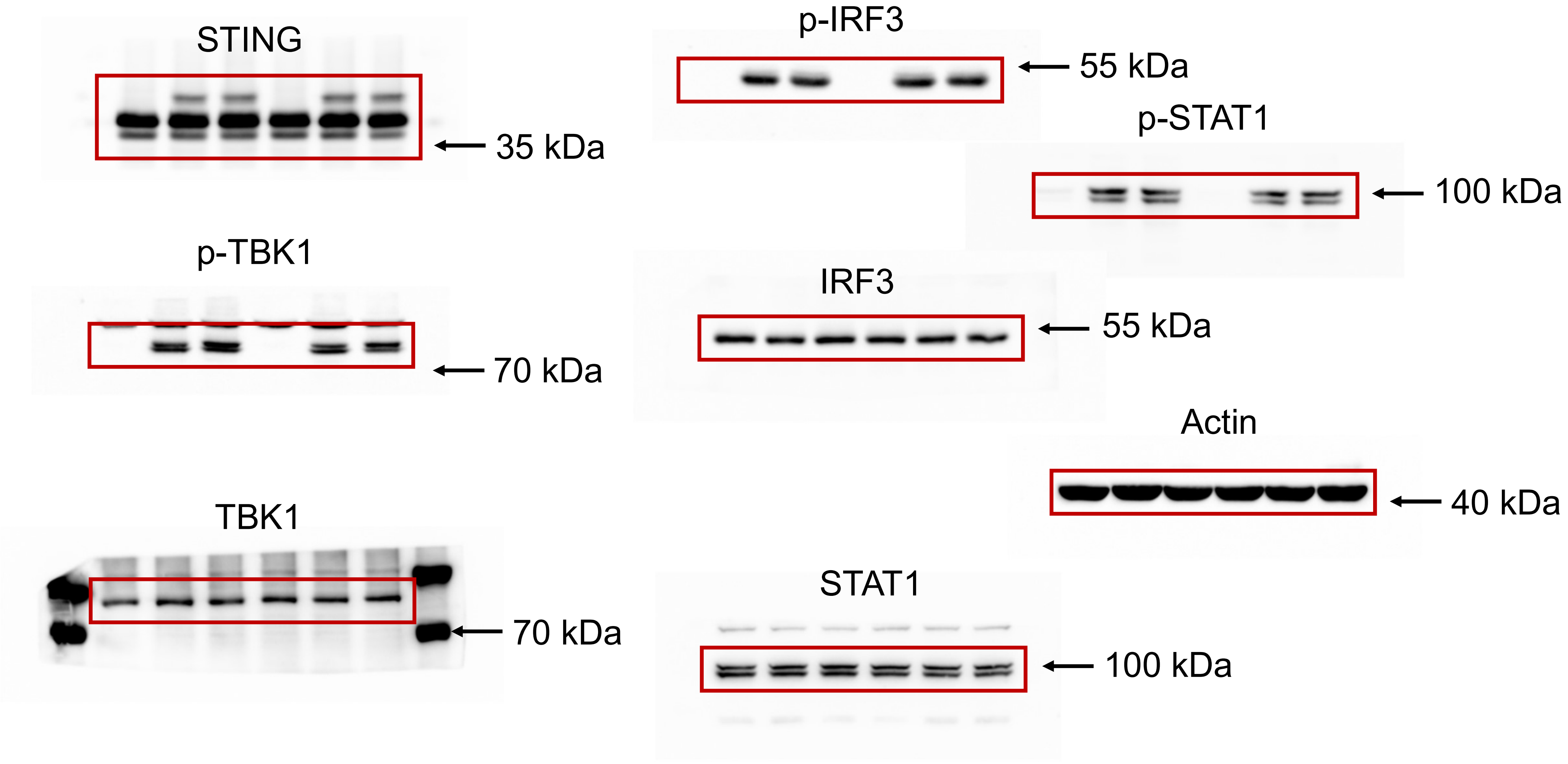

Full unedited blot/gel for Supplementary Figure 3I

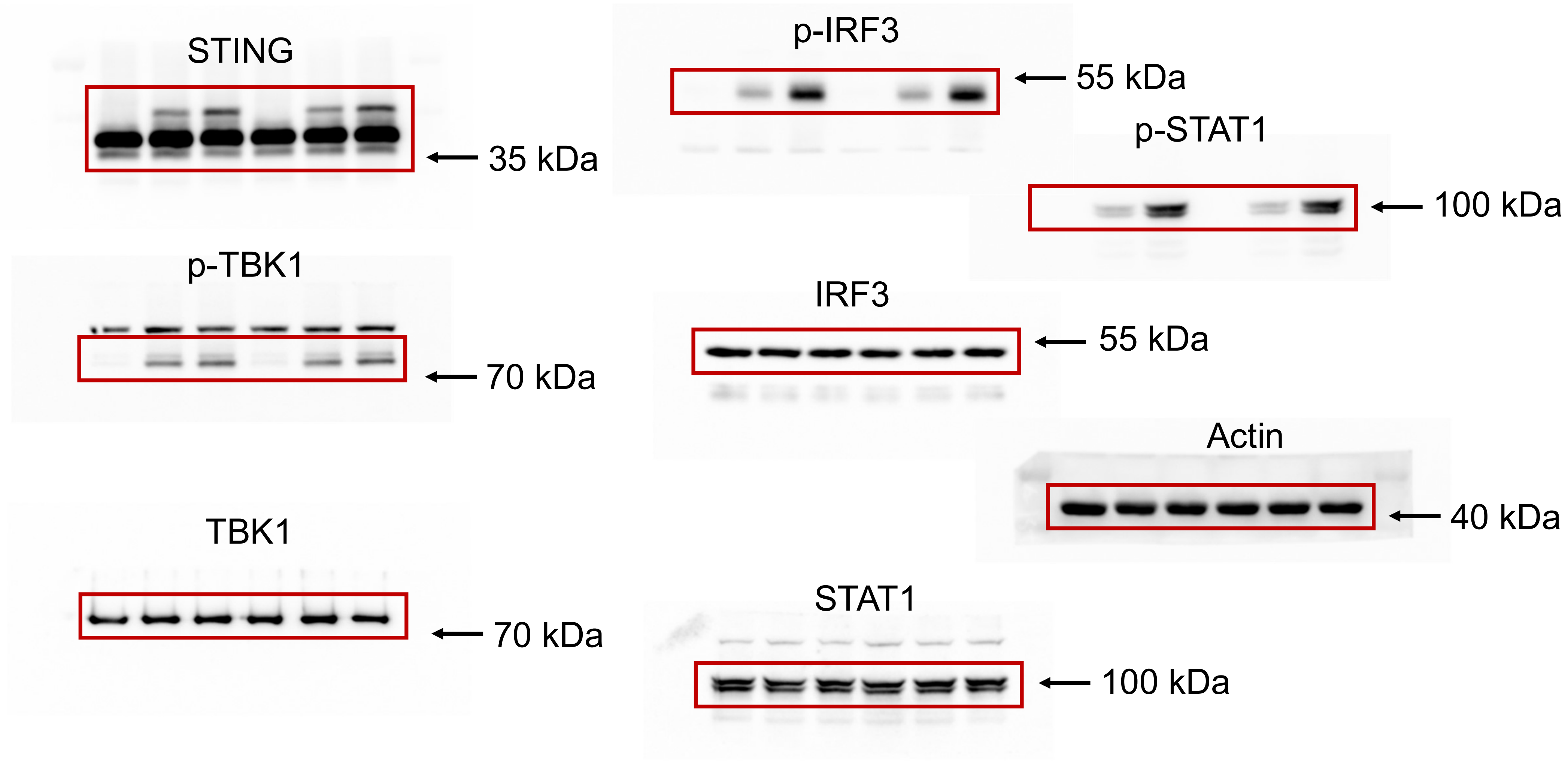

Full unedited blot/gel for Supplementary Figure 3J

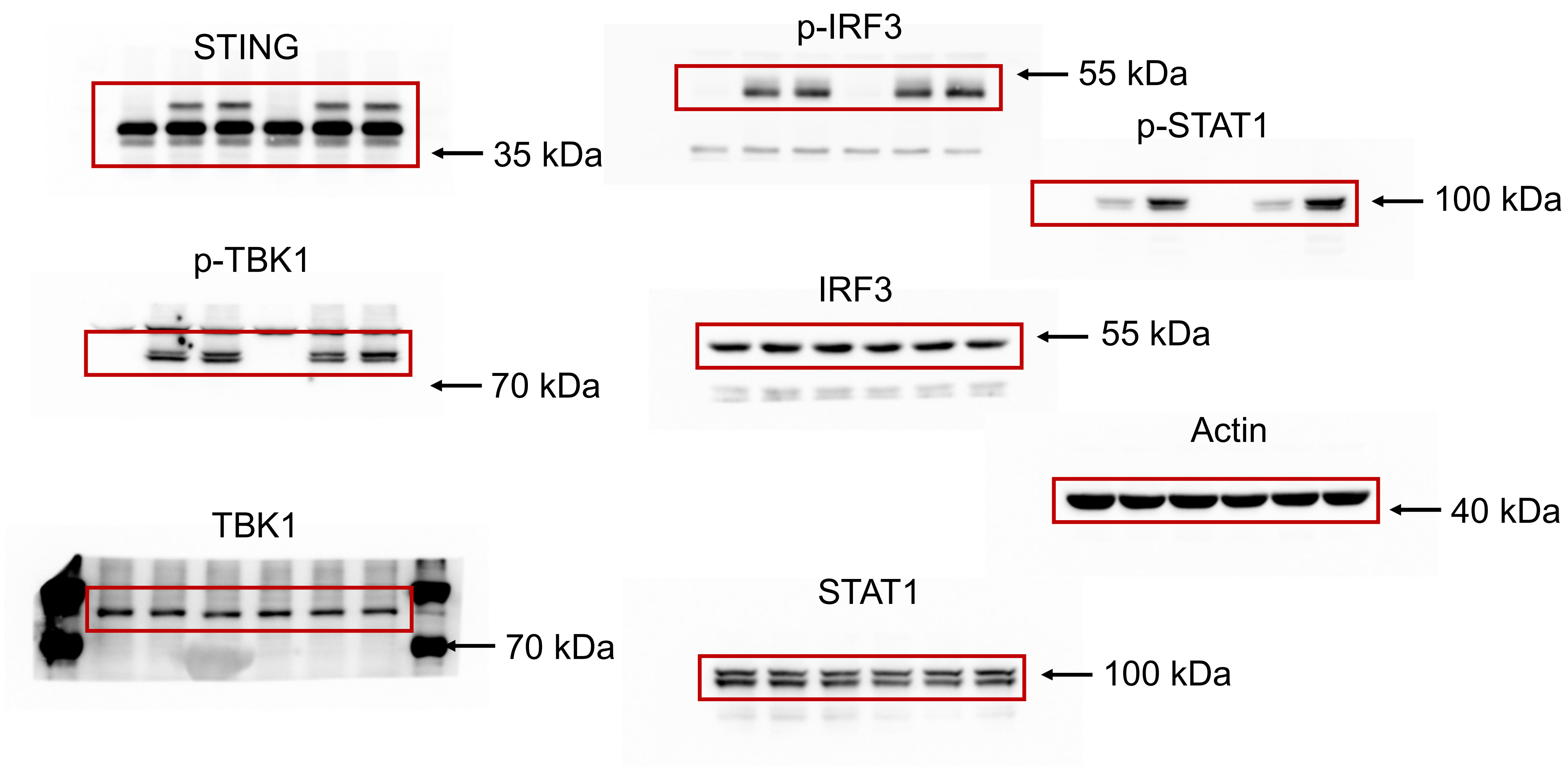

Full unedited blot/gel for Supplementary Figure 4A

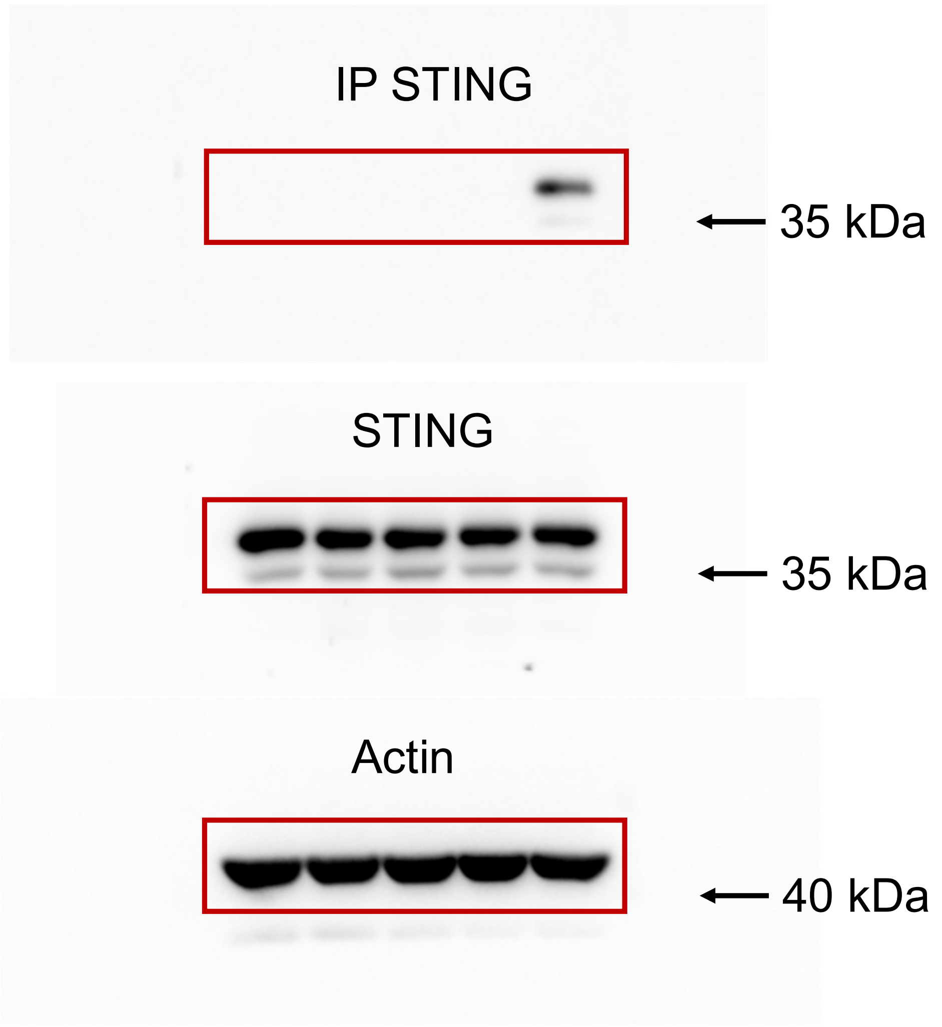

Full unedited blot/gel for Supplementary Figure 5B

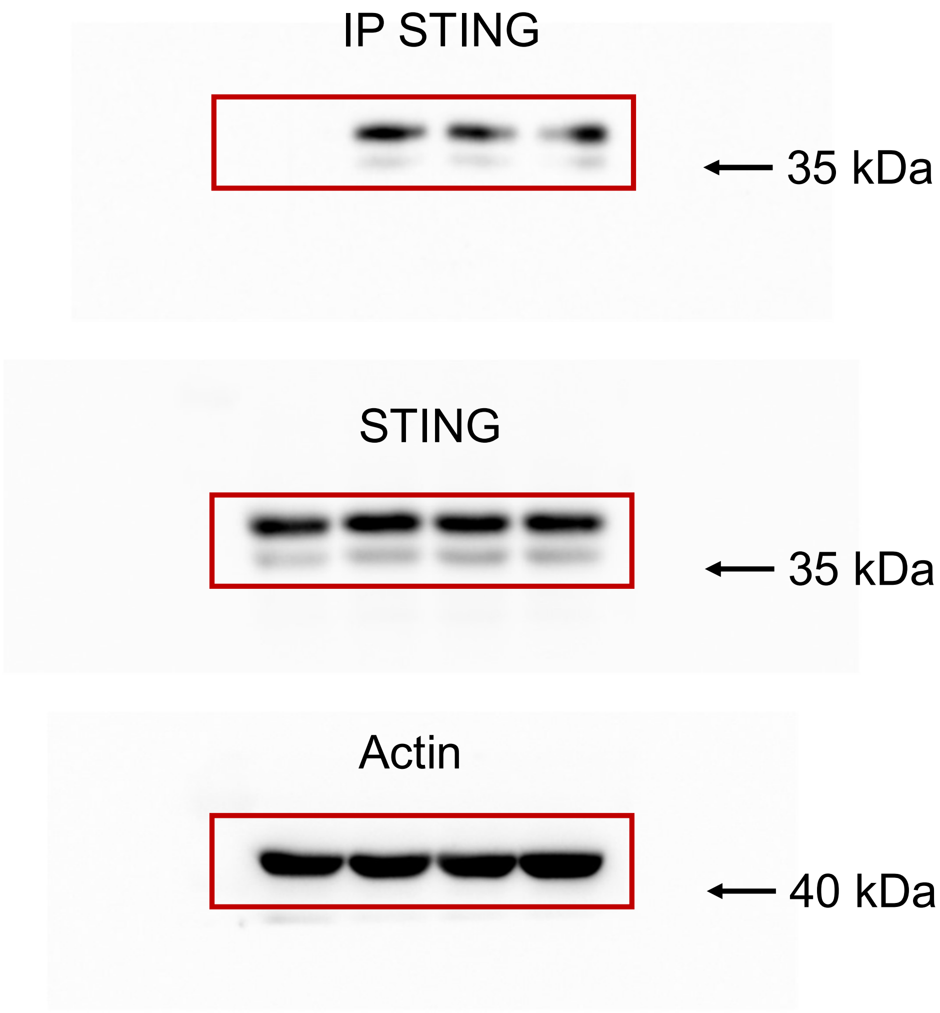

Full unedited blot/gel for Supplementary Figure 5E

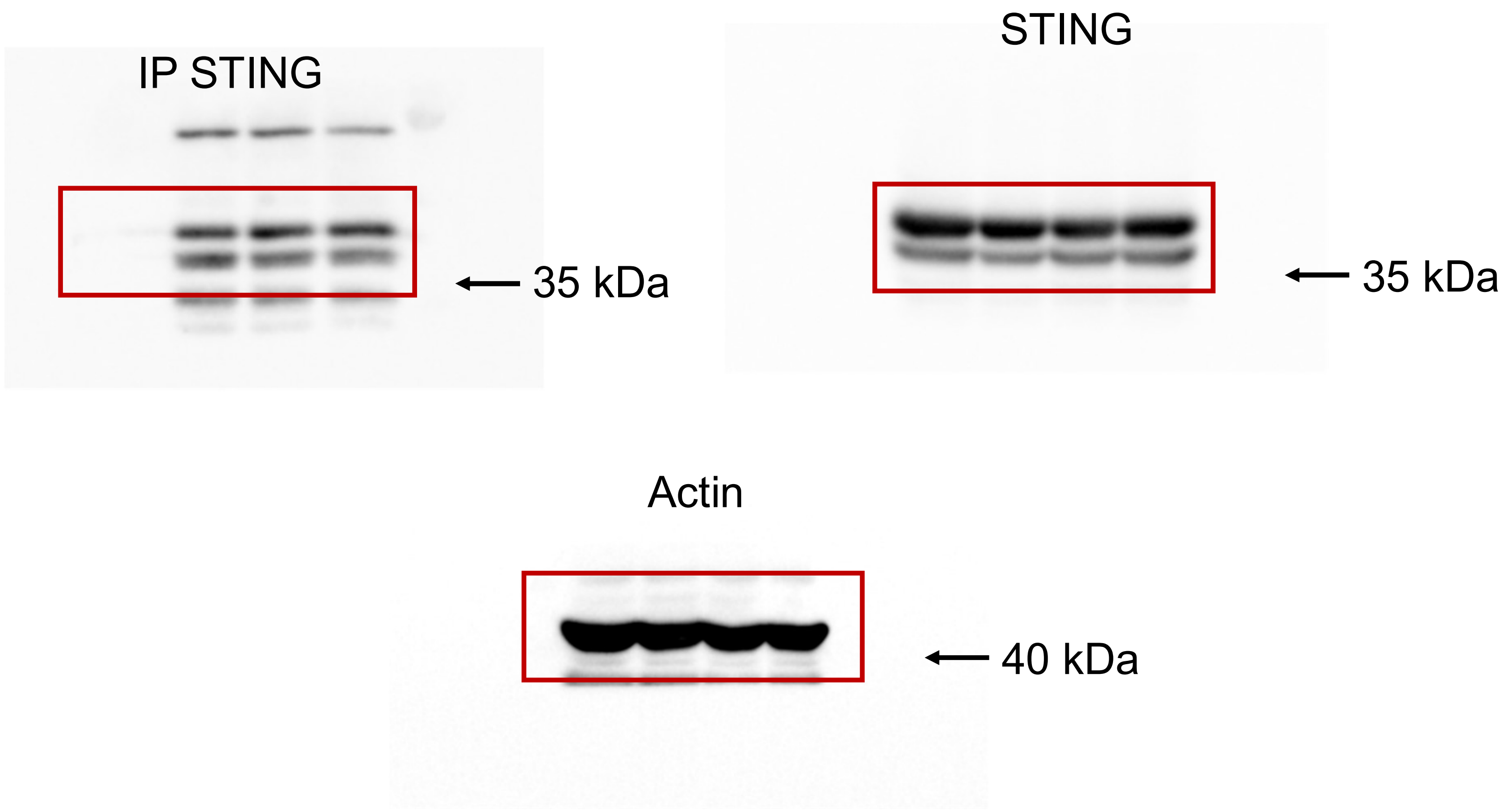

Full unedited blot/gel for Supplementary Figure 5F

IP STING

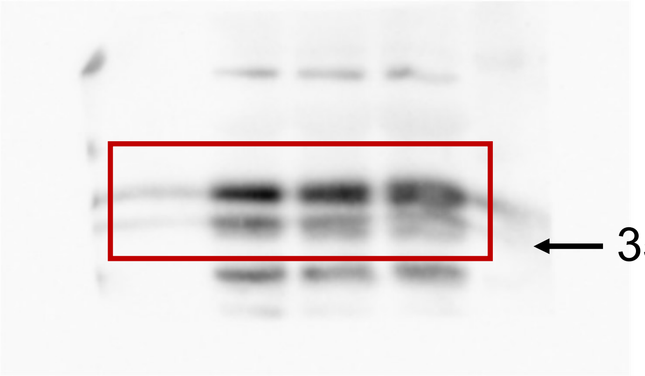

35 kDa

STING

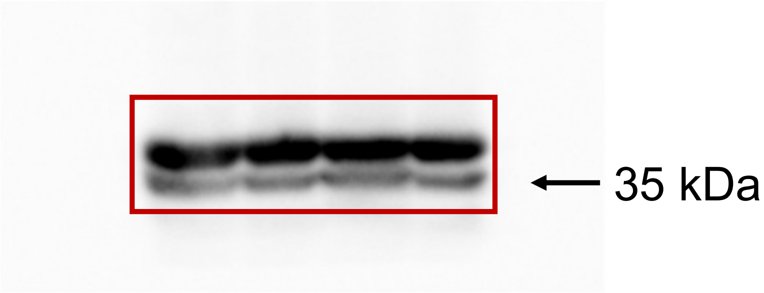

35 kDa

Actin

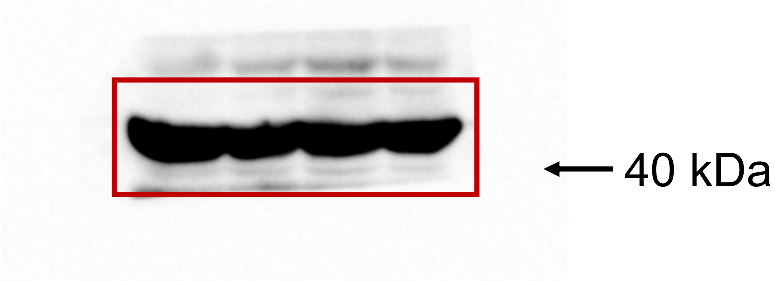

40 kDa

Full unedited blot/gel for Supplementary Figure 5G

IP STING

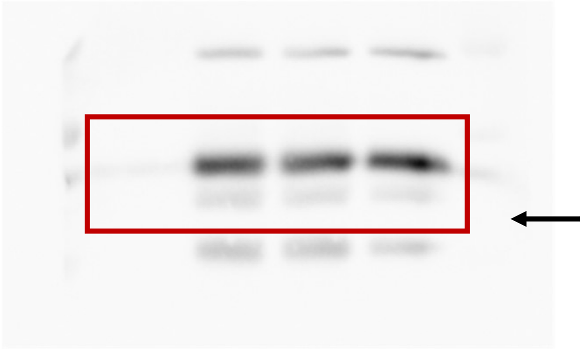

← 35 kDa

STING

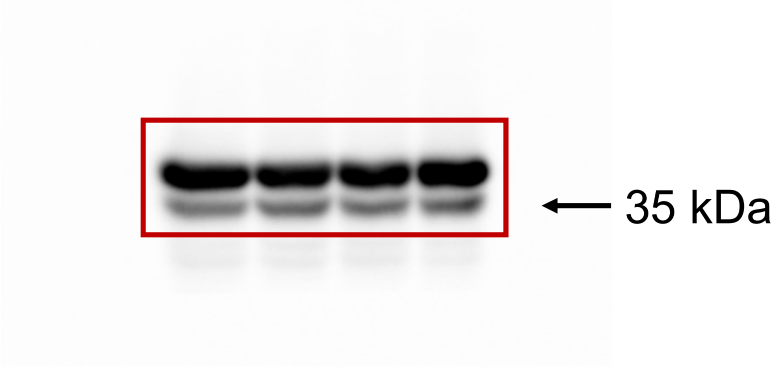

← 35 kDa

Actin

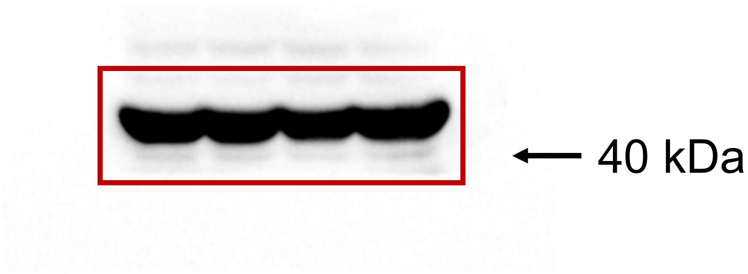

← 40 kDa

Full unedited blot/gel for Supplementary Figure 7A

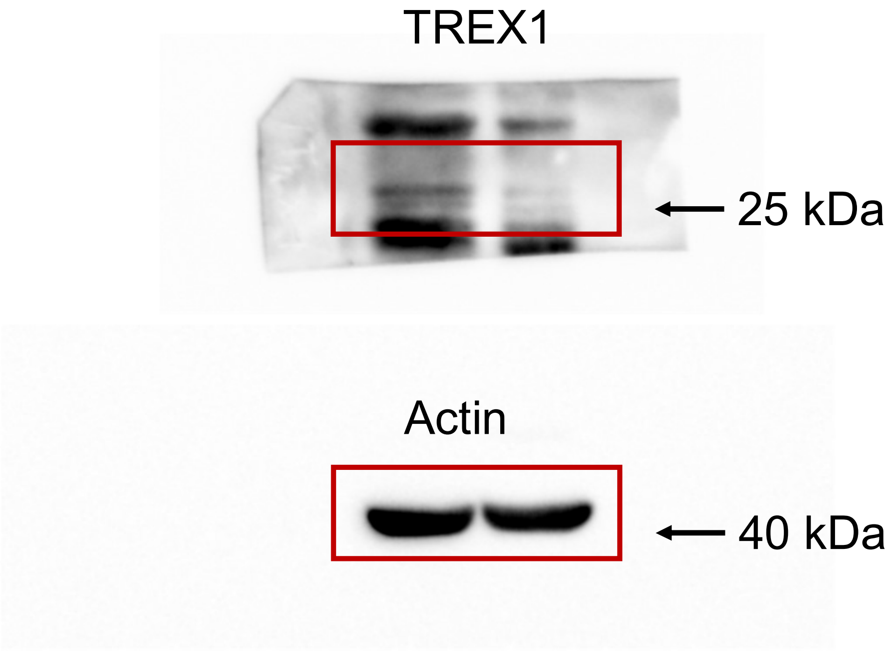

Full unedited blot/gel for Supplementary Figure 8D

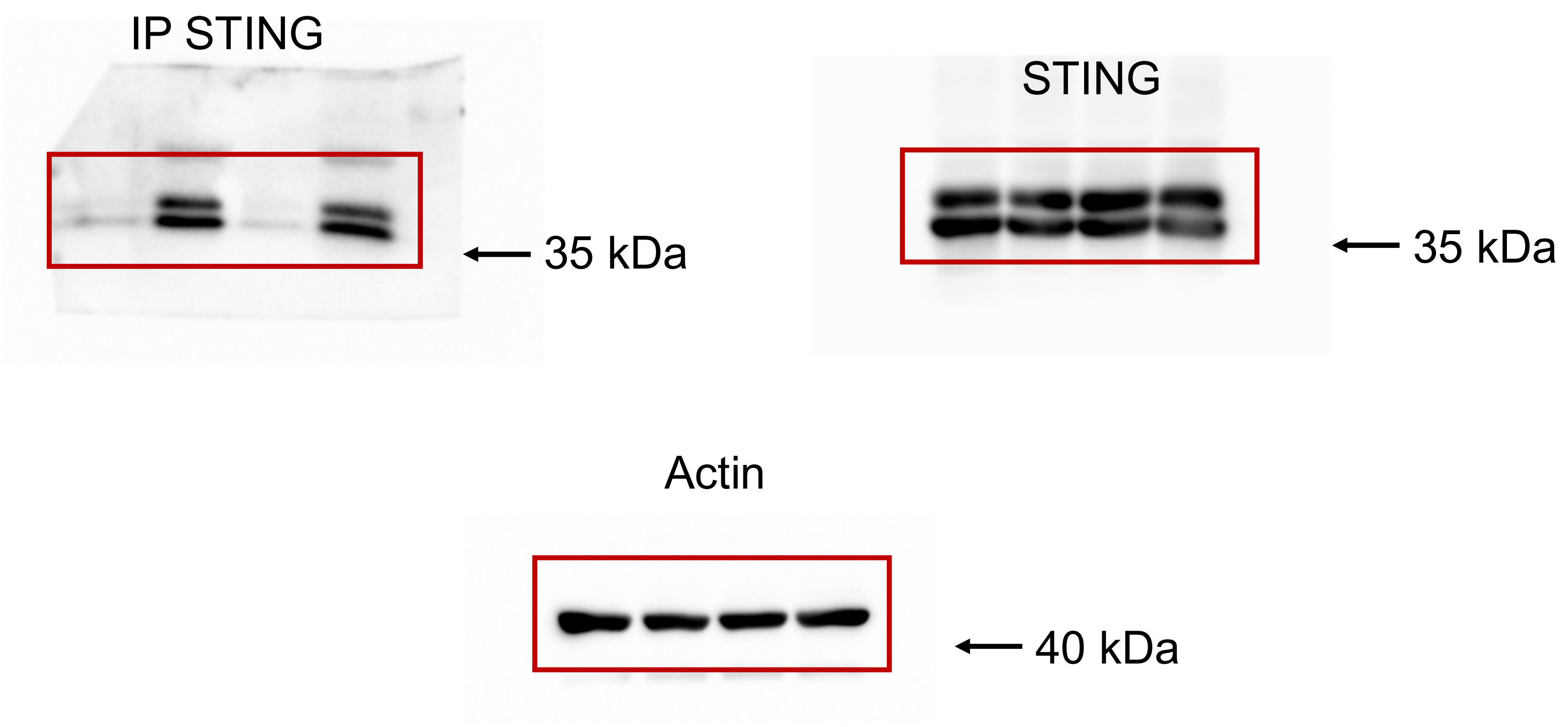

Full unedited blot/gel for Supplementary Figure 8E

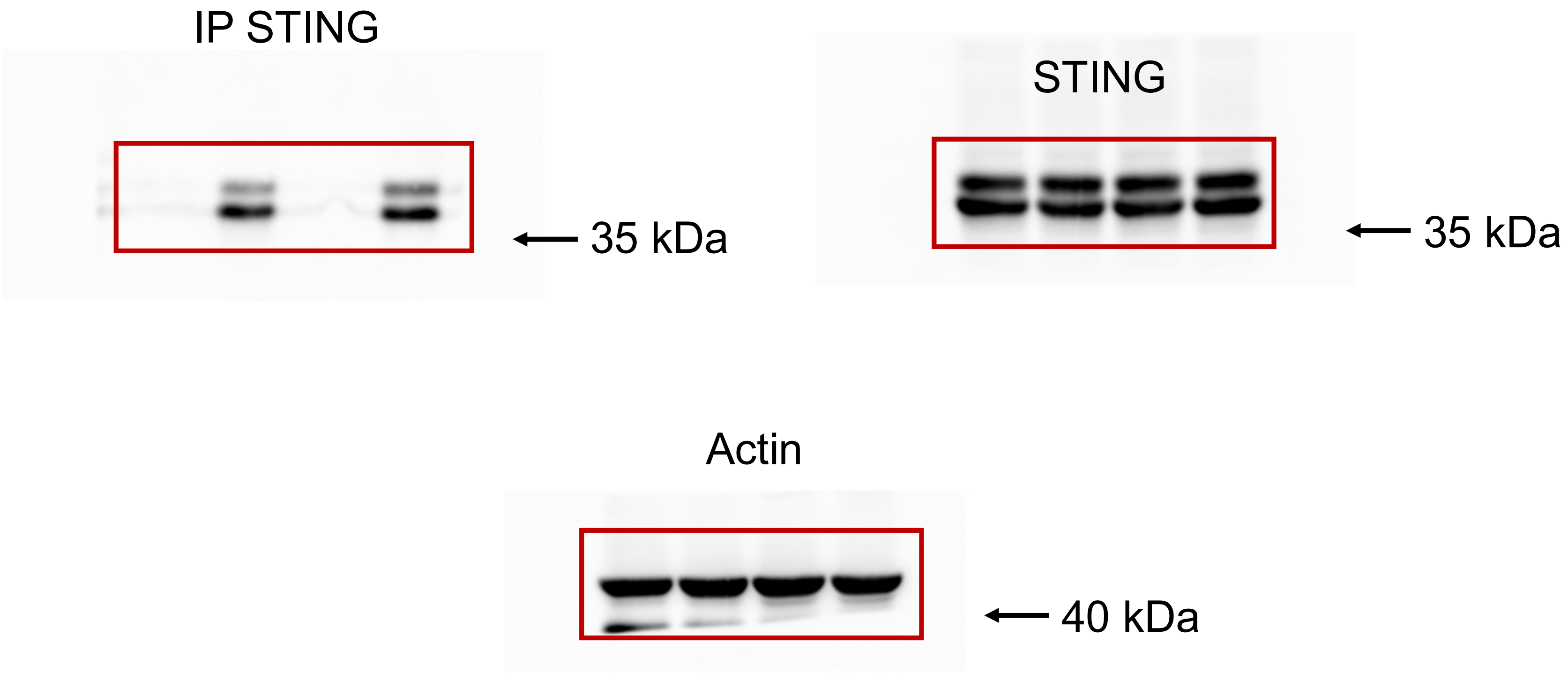

Full unedited blot/gel for Supplementary Figure 8F

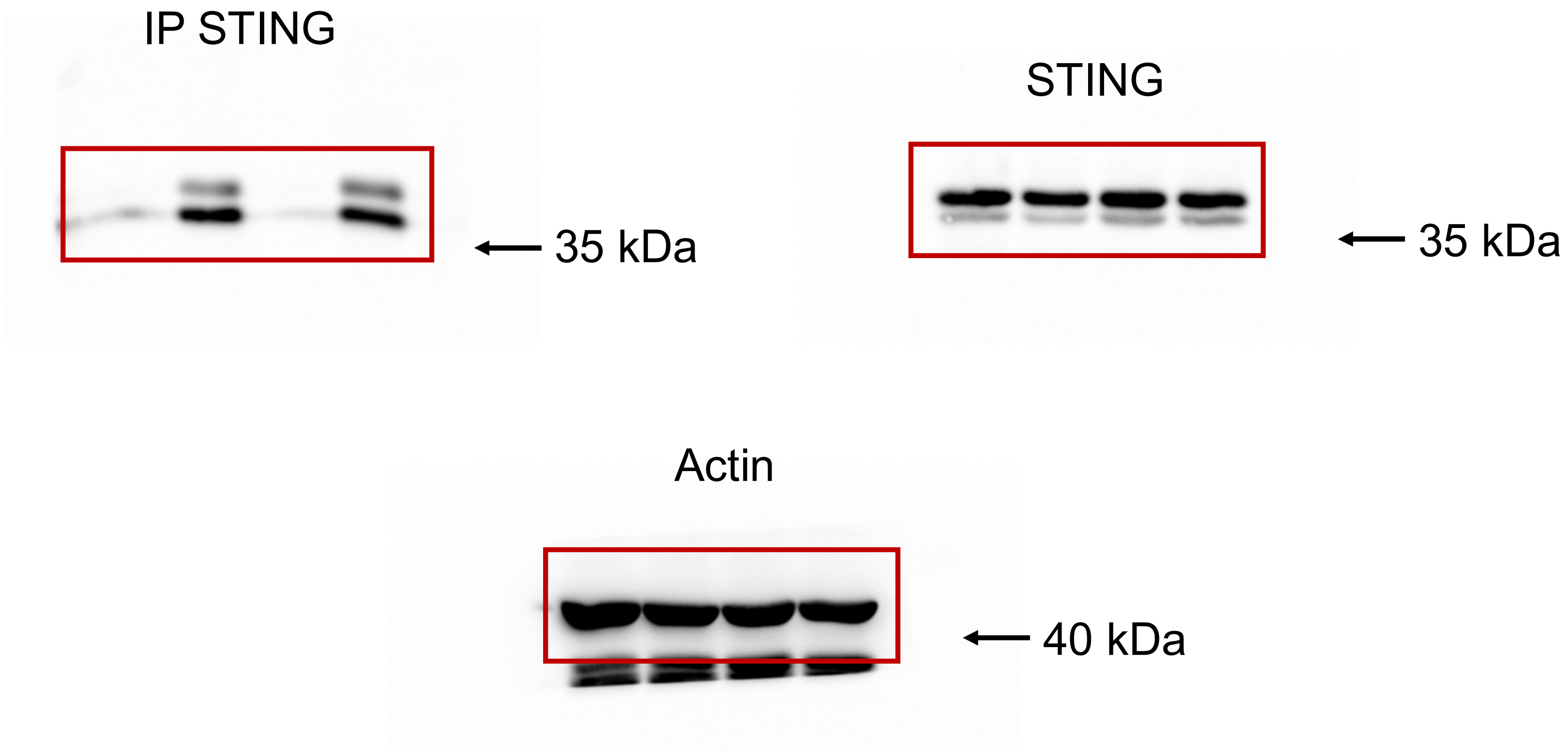

Full unedited blot/gel for Supplementary Figure 8I

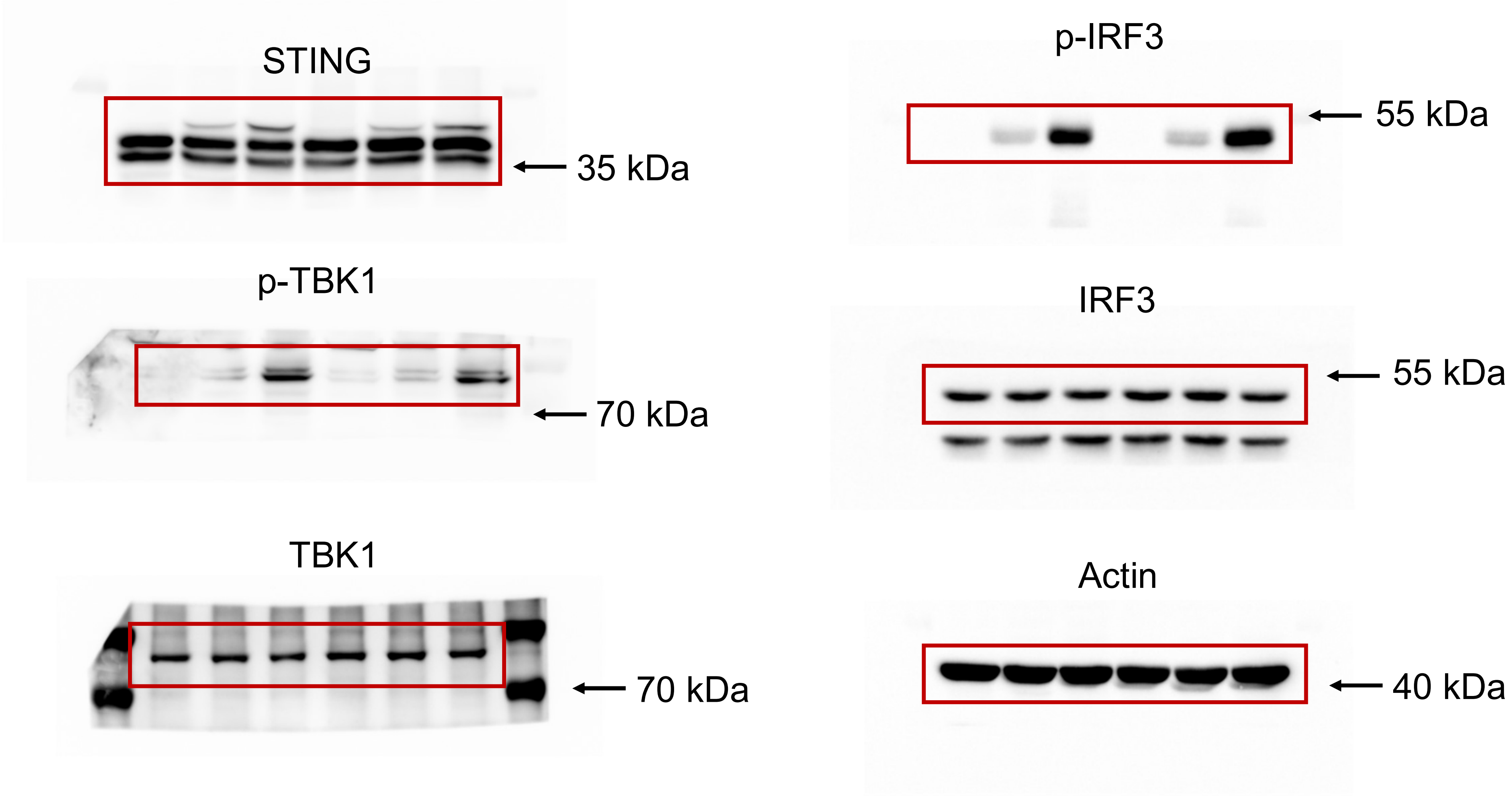

Full unedited blot/gel for Supplementary Figure 8J

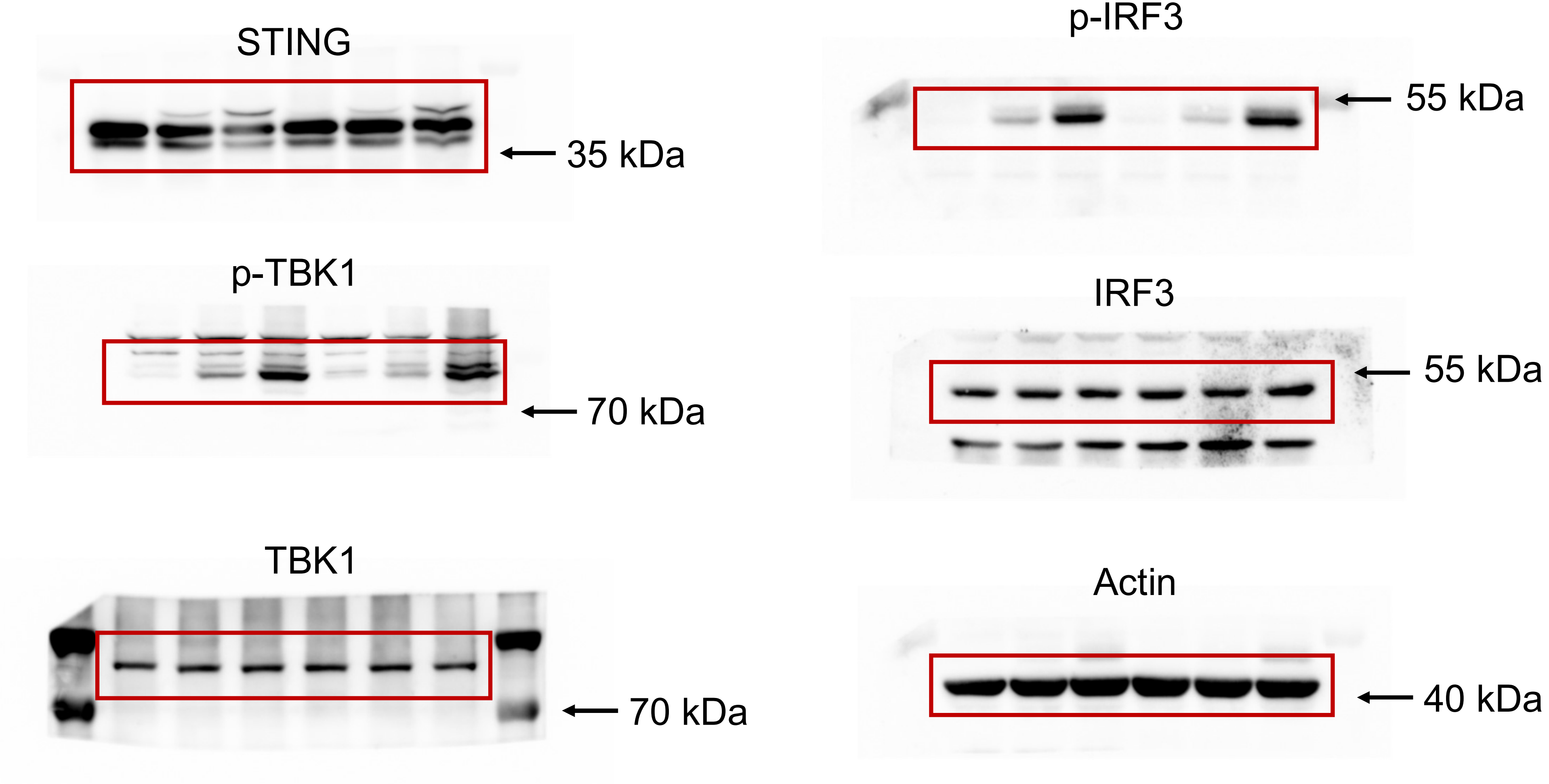

Full unedited blot/gel for Supplementary Figure 8K

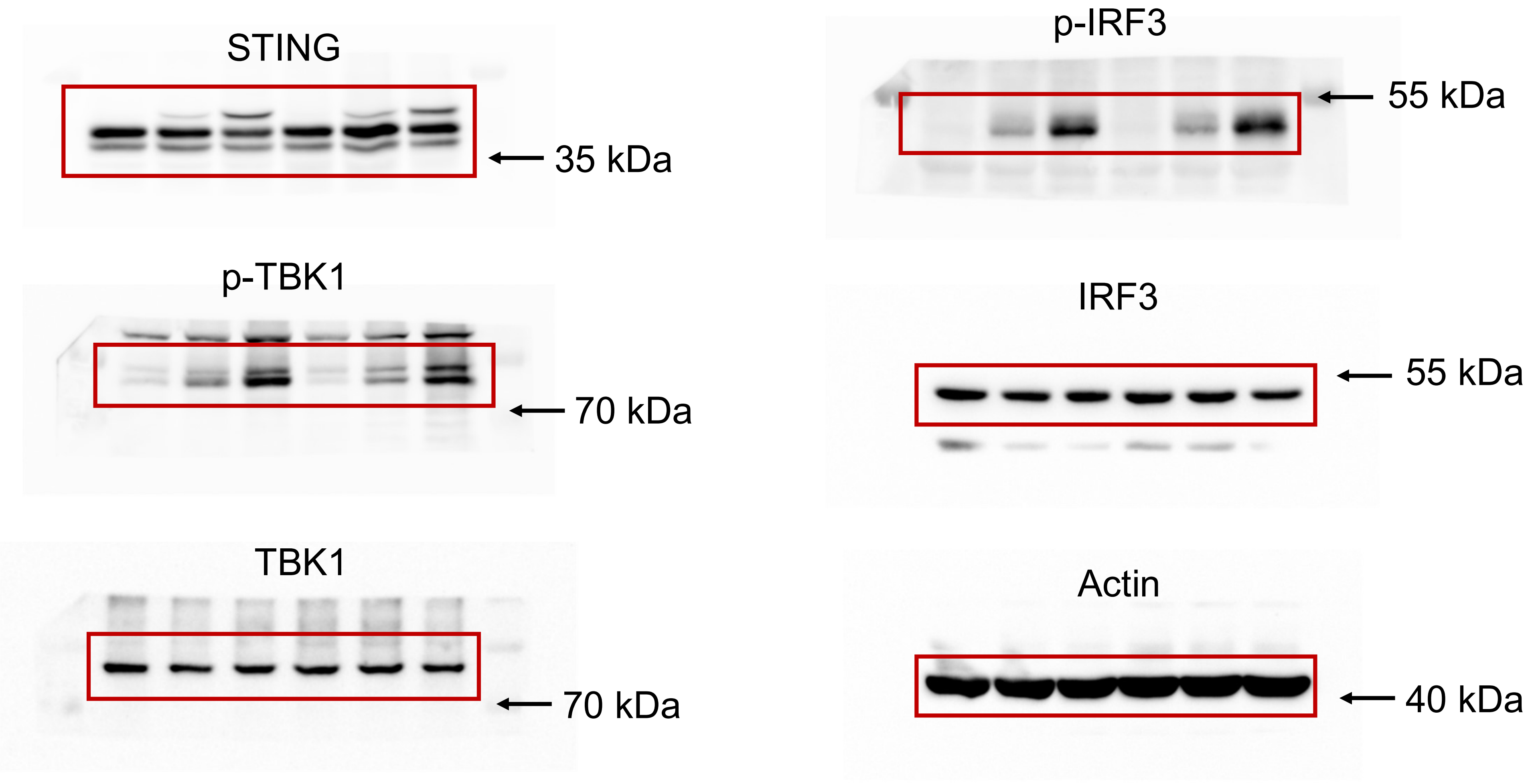

Full unedited blot/gel for Supplementary Figure 8L

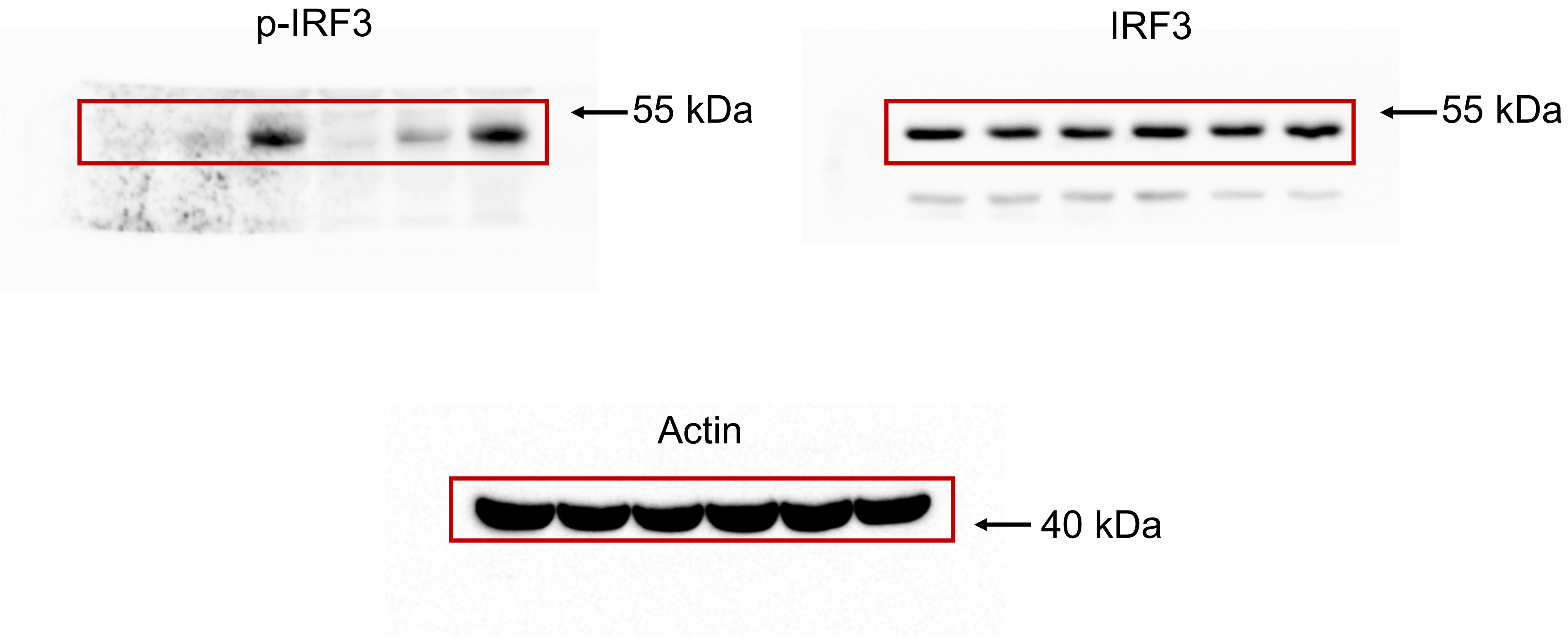

Full unedited blot/gel for Supplementary Figure 8M

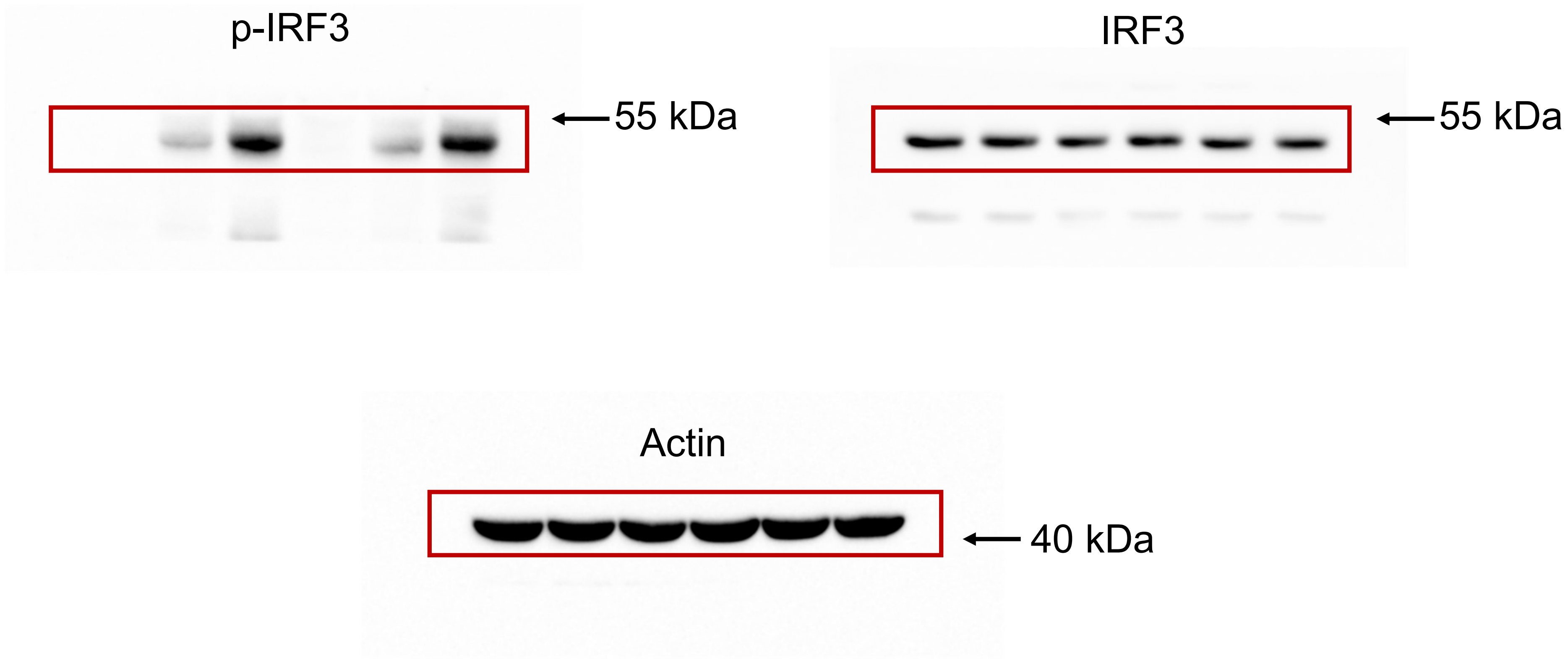

Full unedited blot/gel for Supplementary Figure 8N

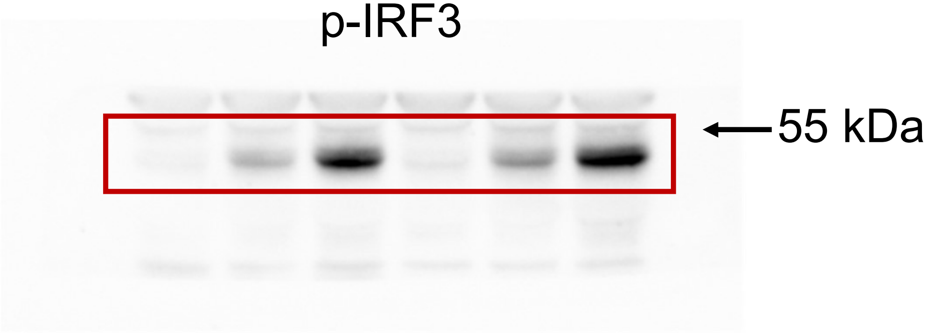

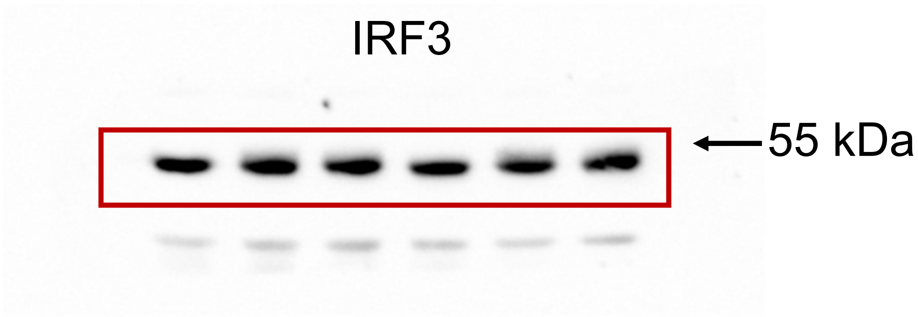

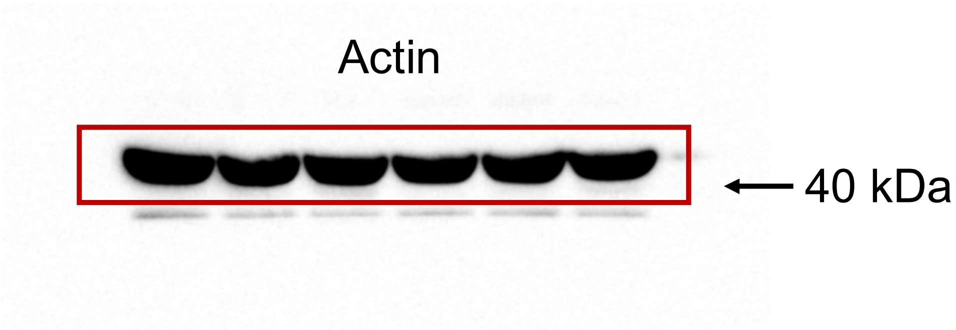

Full unedited blot/gel for Supplementary Figure 9A

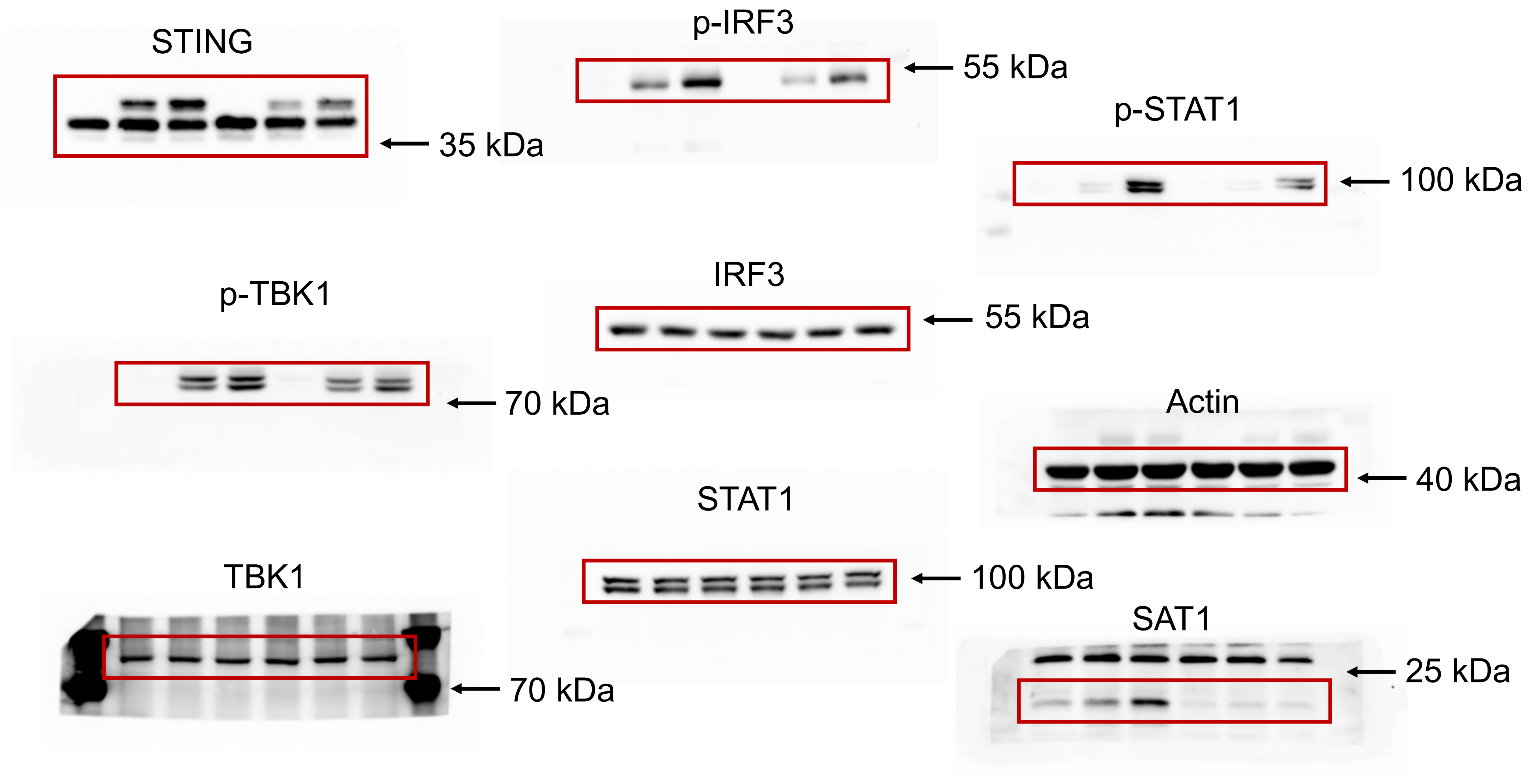

Full unedited blot/gel for Supplementary Figure 9G

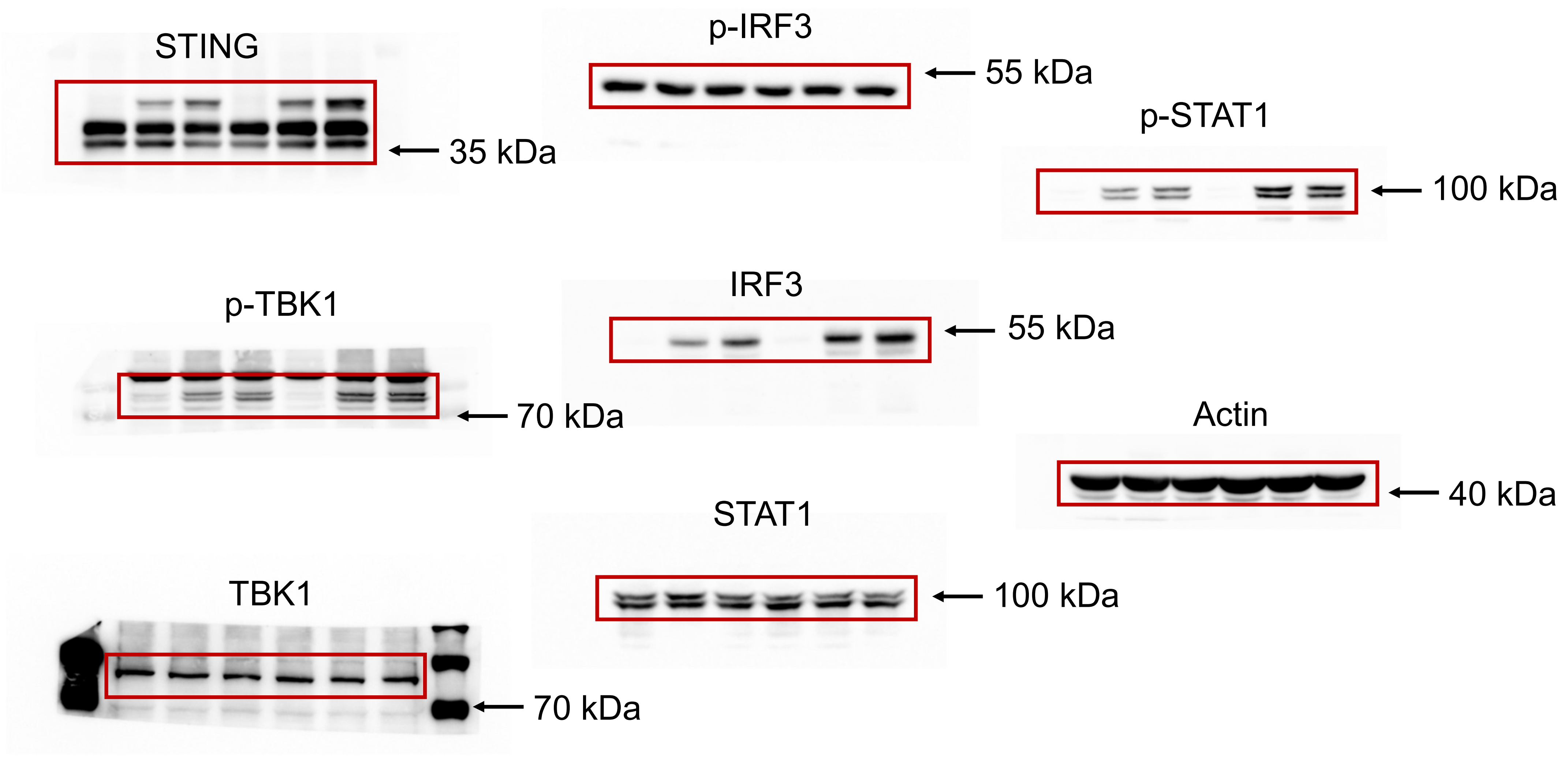

Full unedited blot/gel for Supplementary Figure 15C

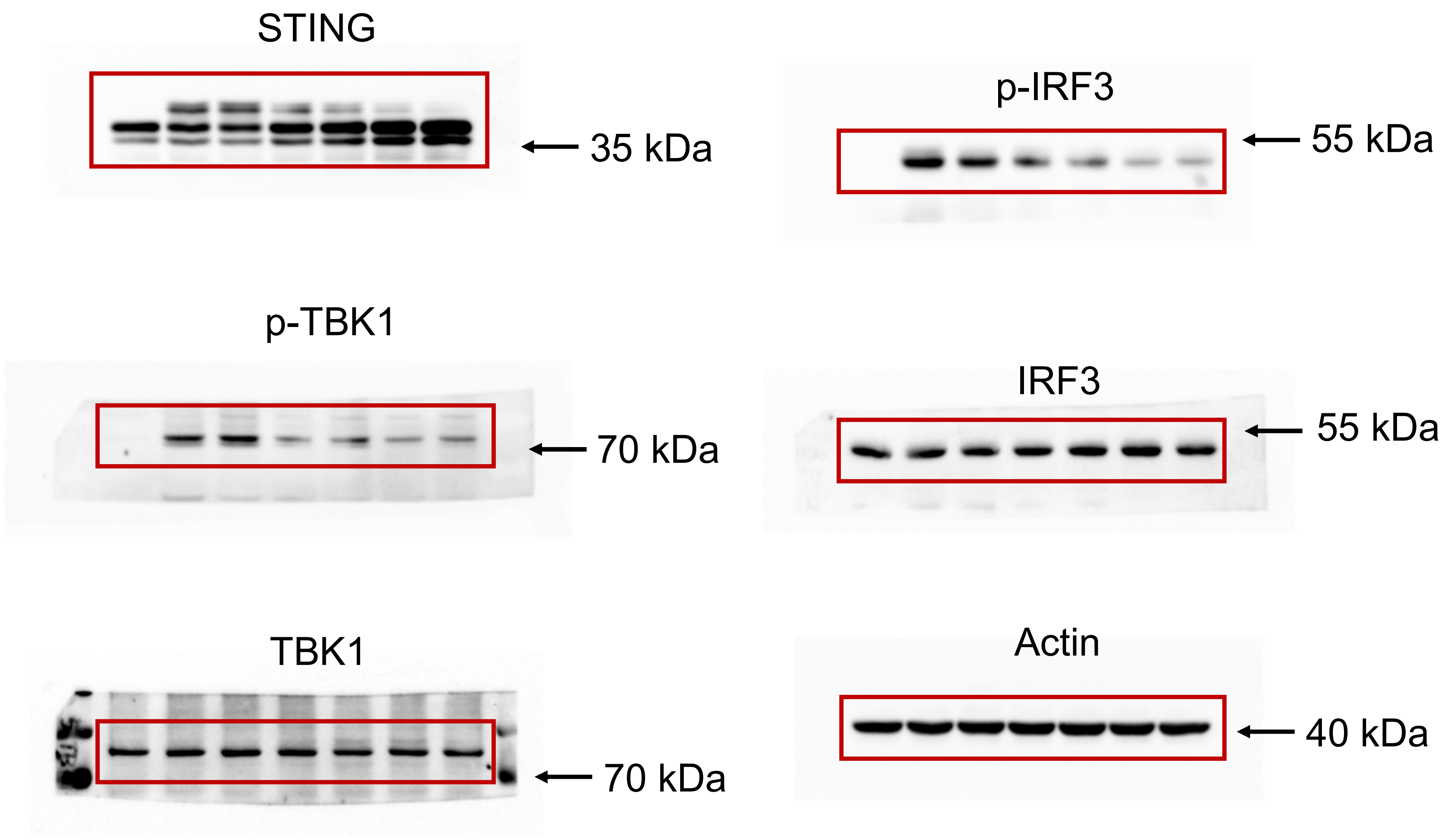

Full unedited blot/gel for Supplementary Figure 15D

STING

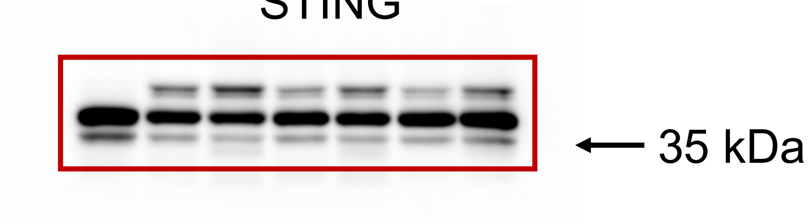

p-IRF3

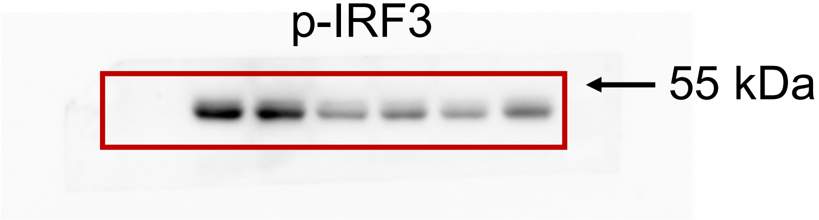

p-TBK1

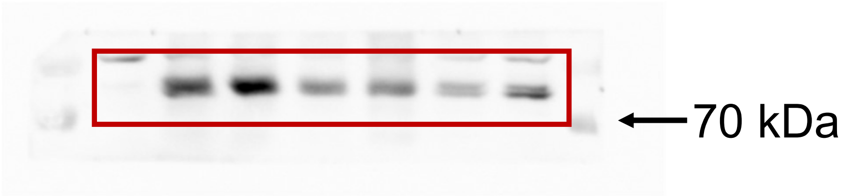

IRF3

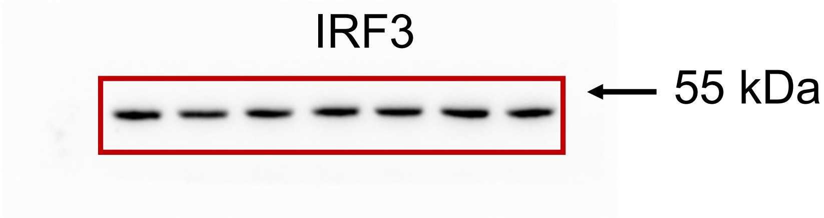

TBK1

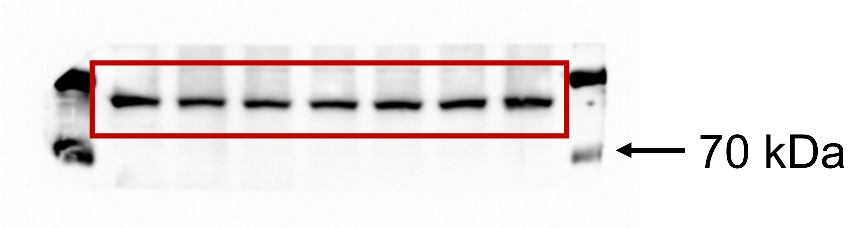

Actin

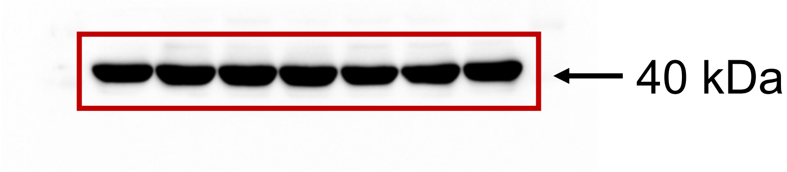

Full unedited blot/gel for Supplementary Figure 15E

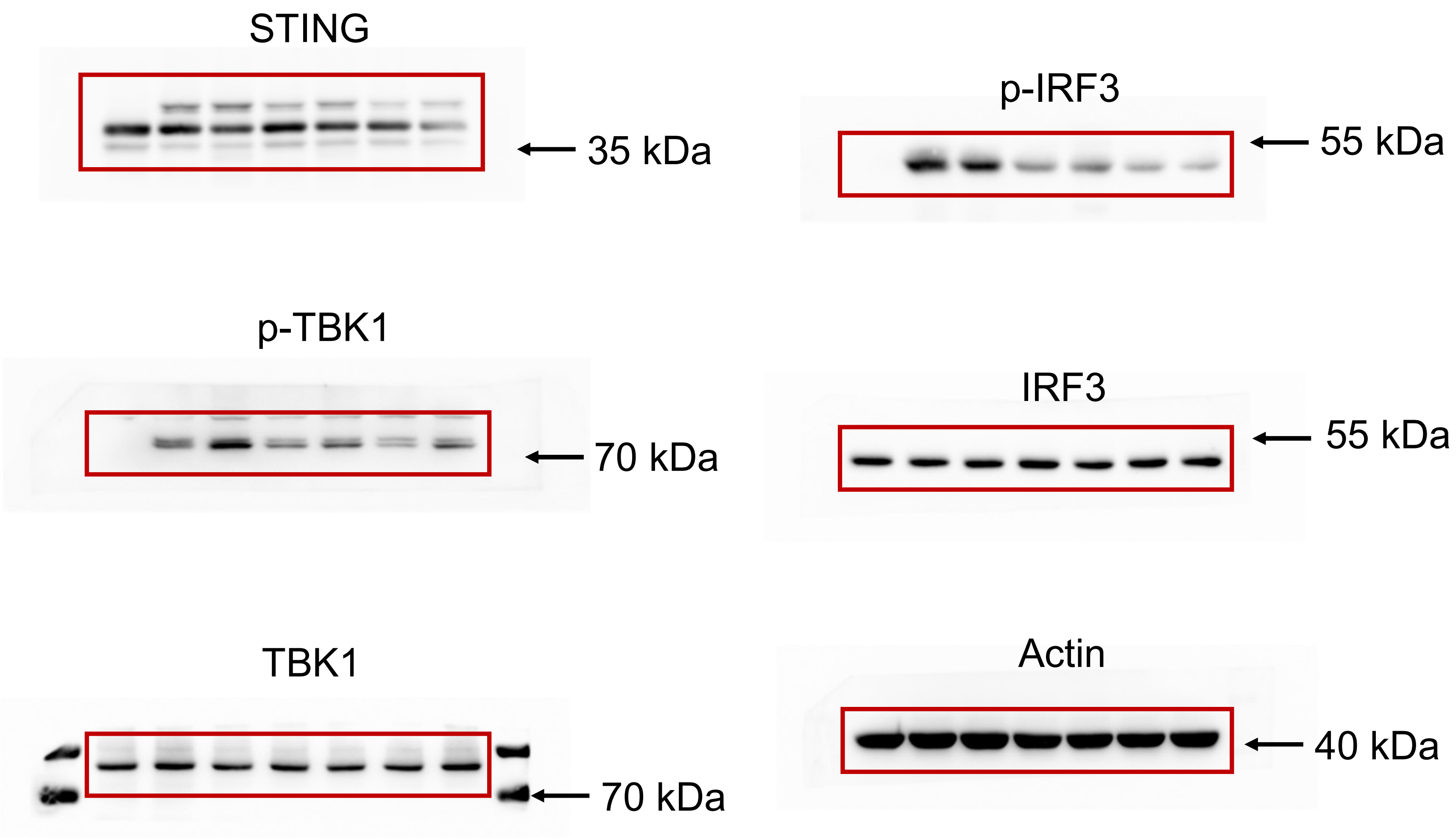

Full unedited blot/gel for Supplementary Figure 15H

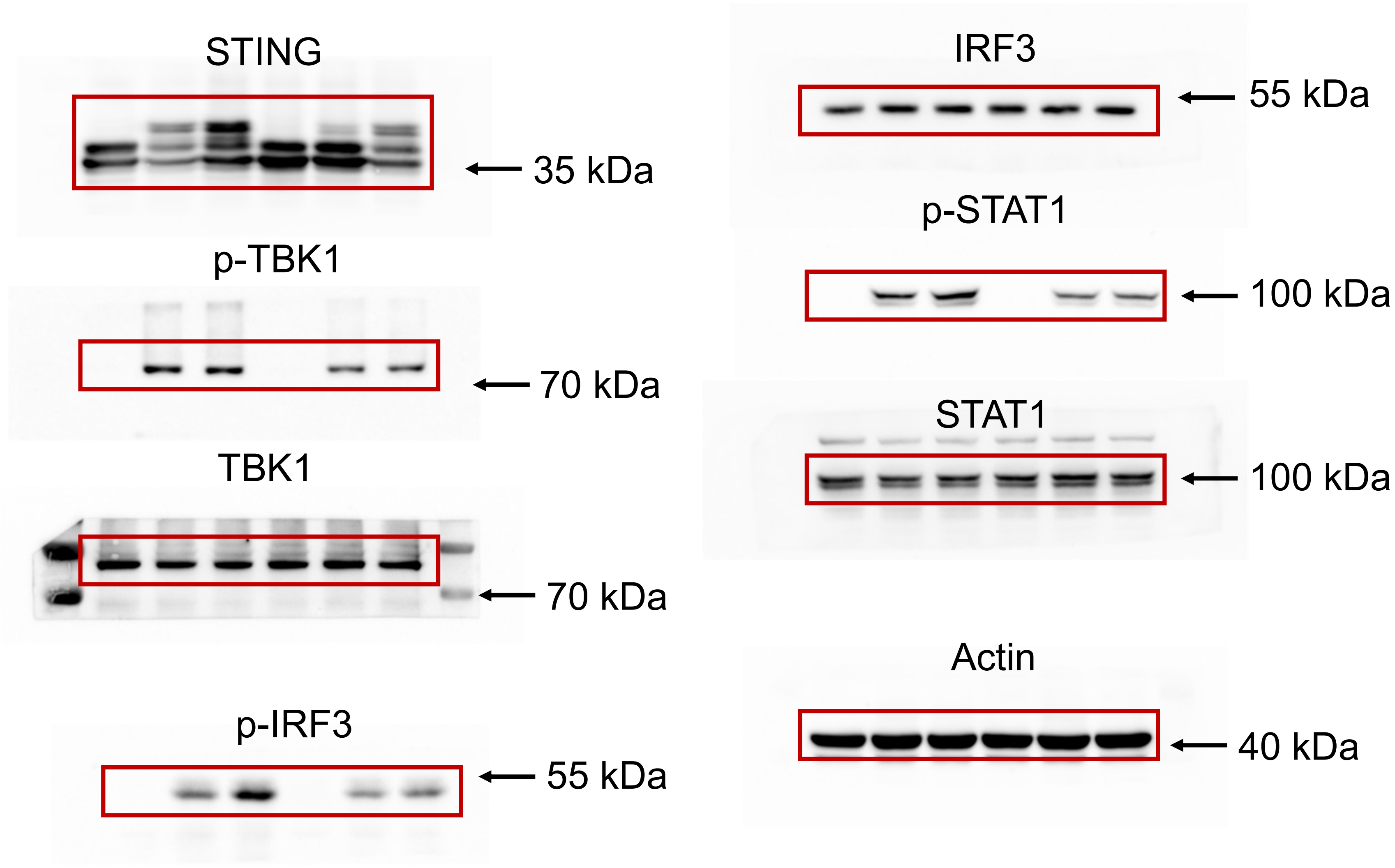

Full unedited blot/gel for Supplementary Figure 15I

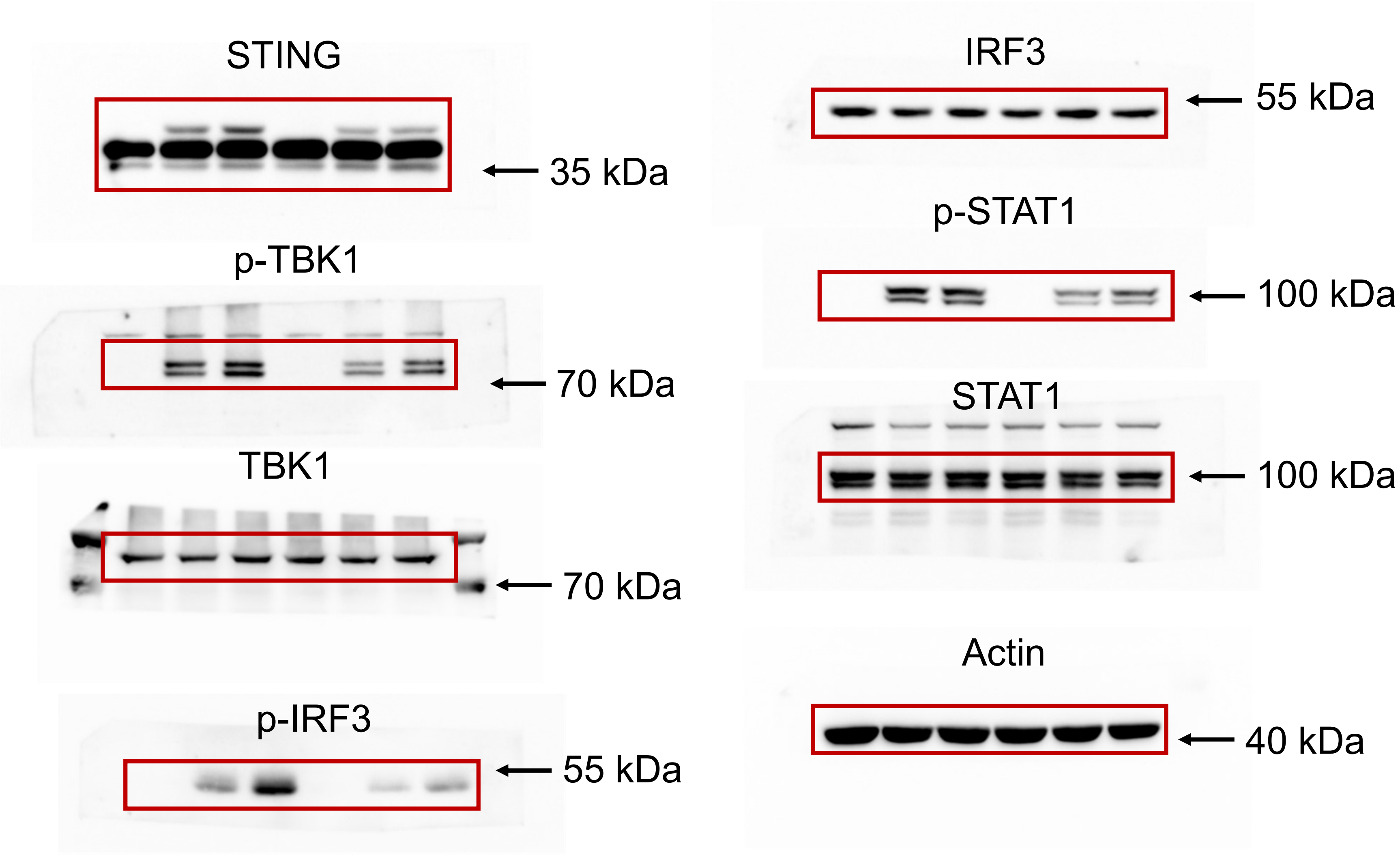

Full unedited blot/gel for Supplementary Figure 15J

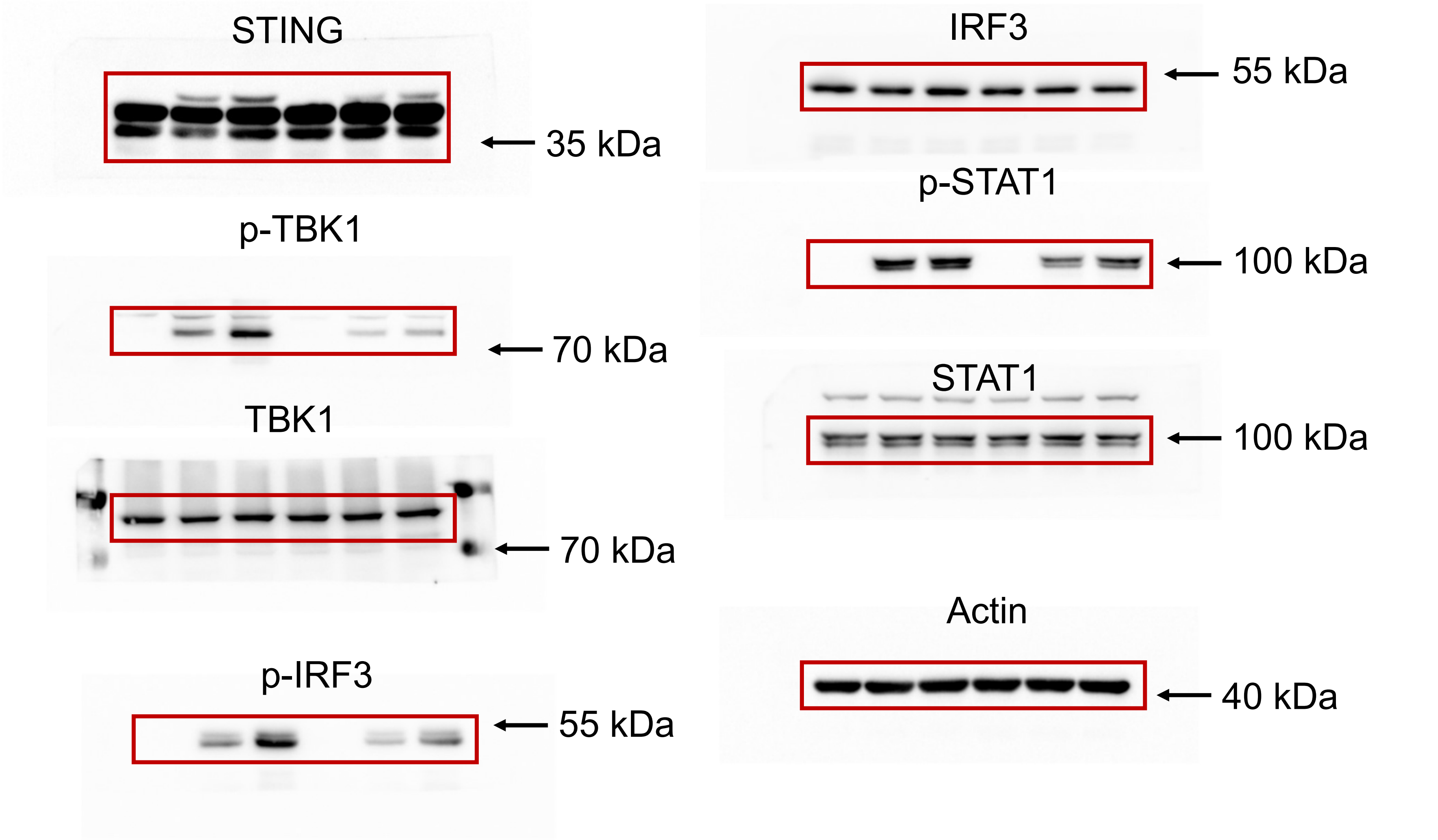

Supplement: Unedited blot and gel images [file jci-136-201460-s232.pdf]
